# Publisher Correction: Functional control of a 0.5 MDa TET aminopeptidase by a flexible loop revealed by MAS NMR

**DOI:** 10.1038/s41467-022-31243-1

**Published:** 2022-06-23

**Authors:** Diego F. Gauto, Pavel Macek, Duccio Malinverni, Hugo Fraga, Matteo Paloni, Iva Sučec, Audrey Hessel, Juan Pablo Bustamante, Alessandro Barducci, Paul Schanda

**Affiliations:** 1 Univ. Grenoble Alpes, CEA, CNRS, Institut de Biologie Structurale (IBS), 71, Avenue des Martyrs, F-38044 Grenoble, France; 2 Department of Structural Biology and Center for Data Driven Discovery, St Jude Children’s Research Hospital, Memphis, TN USA; 3 Departamento de Biomedicina, Faculdade de Medicina da Universidade do Porto, Porto, Portugal; 4 i3S, Instituto de Investigacao e Inovacao em Saude, Universidade do Porto, Porto, Portugal; 5 CBS (Centre de Biologie Structurale), Univ Montpellier, CNRS, INSERM, Montpellier, France; 6 Instituto de Bioingenieria y Bioinformatica, IBB (CONICET-UNER), Oro Verde, Entre Rios Argentina; 7 Institute of Science and Technology Austria, Am Campus 1, A-3400 Klosterneuburg, Austria; 8 Present Address: ICSN, CNRS UPR2301, Univ. Paris-Saclay, Gif-sur-Yvette, France; 9 Present Address: Celonic AG, Eulerstrasse 55, 4051 Basel, Switzerland

**Keywords:** Solid-state NMR, Molecular modelling

Correction to: *Nature Communications* 10.1038/s41467-022-29423-0, published online 08 April 2022.

In this article the affiliation details for Alessandro Barducci were incorrectly given as ‘Departamento de Biomedicina, Faculdade de Medicina da Universidade do Porto, Porto, Portugal’ but should have been ‘CBS (Centre de Biologie Structurale), Univ Montpellier, CNRS, INSERM, Montpellier, France’. The original article has been corrected.

