# Supplementary figures and images for: Functional control of a 0.5 MDa TET aminopeptidase by a flexible loop revealed by MAS NMR
Source: Nat Commun. 2022 Apr 8;13:1927. doi: 10.1038/s41467-022-29423-0 (PMC8993905; doi:10.1038/s41467-022-29423-0)

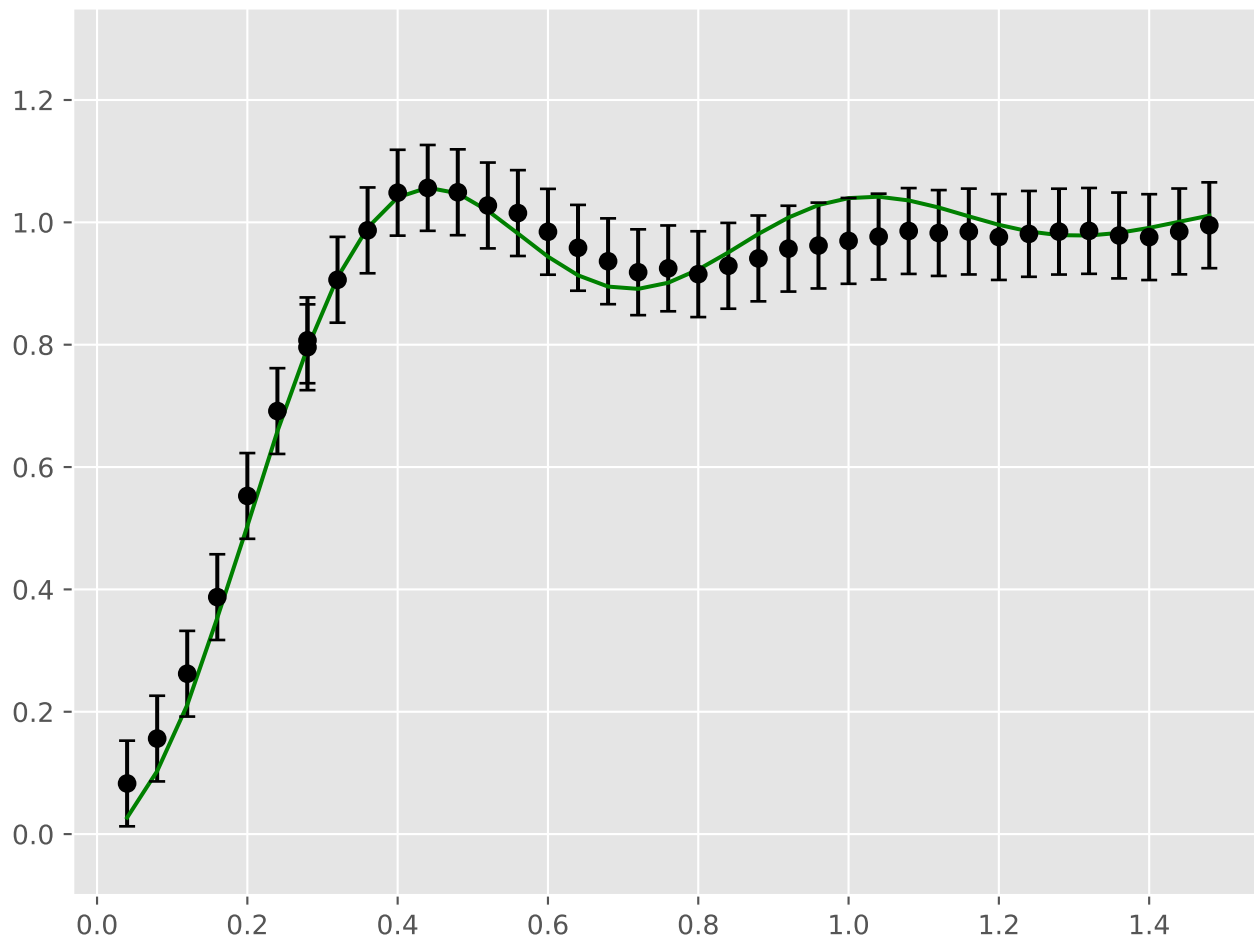

Supplement: Supplementary file 4 — Source data [file 41467_2022_29423_MOESM4_ESM.gz › source_data_2022/Figures_2B-E_SI5_SI6_SI7/Methyl dipolar-coupling measurements. Exptl data, simulations and analysis scripts/fit-figures/44.pdf]

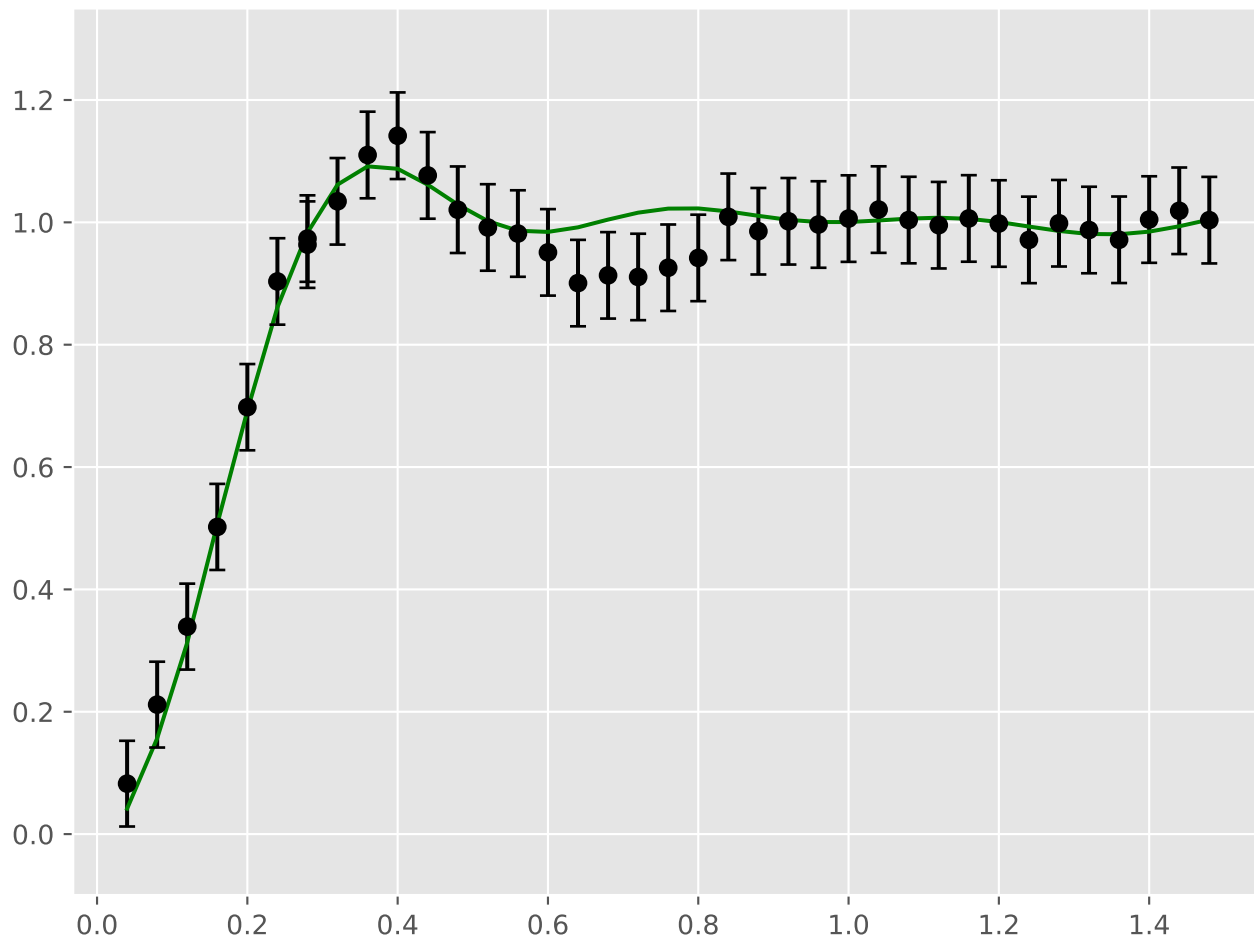

Supplement: Supplementary file 4 — Source data [file 41467_2022_29423_MOESM4_ESM.gz › source_data_2022/Figures_2B-E_SI5_SI6_SI7/Methyl dipolar-coupling measurements. Exptl data, simulations and analysis scripts/fit-figures/41.pdf]

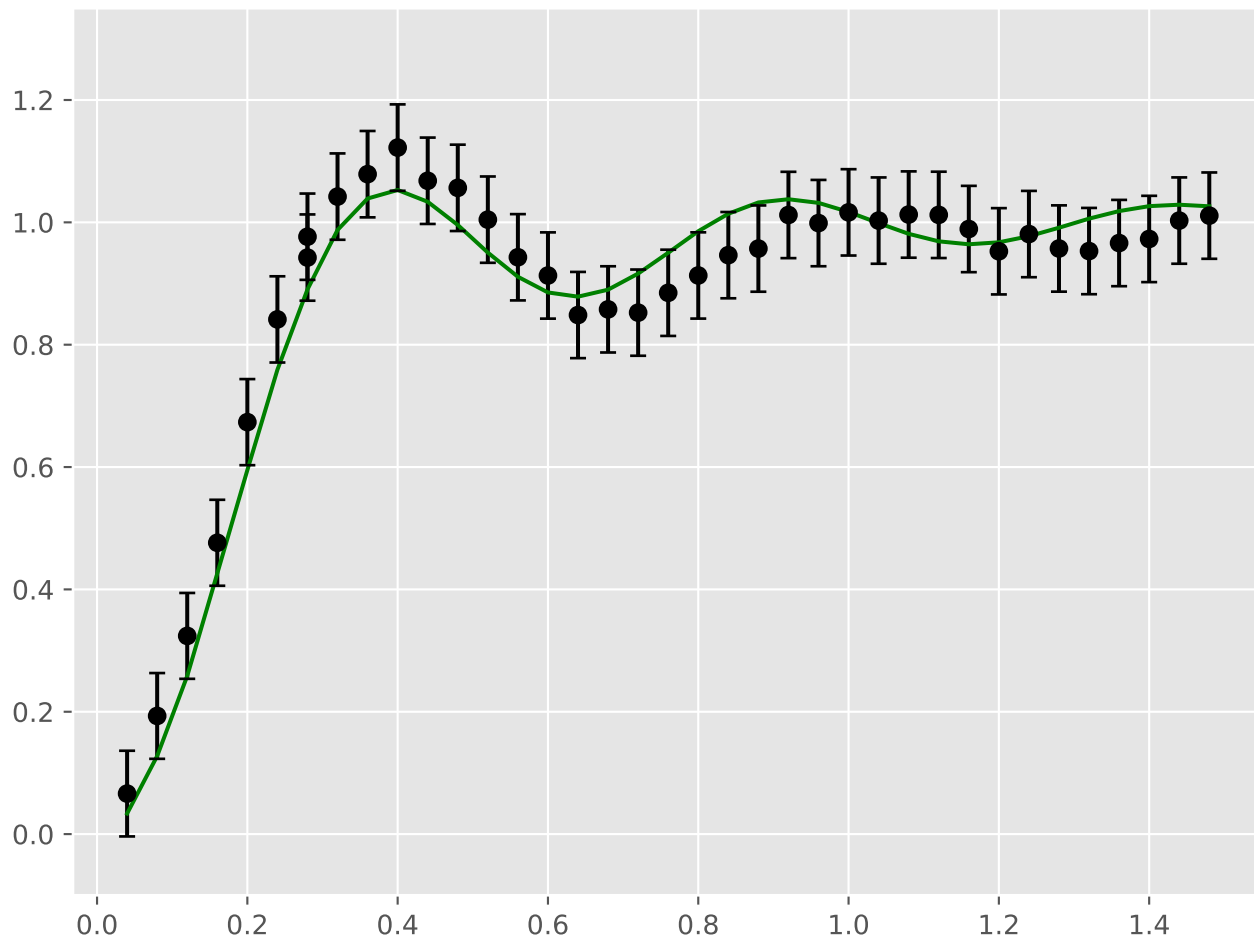

Supplement: Supplementary file 4 — Source data [file 41467_2022_29423_MOESM4_ESM.gz › source_data_2022/Figures_2B-E_SI5_SI6_SI7/Methyl dipolar-coupling measurements. Exptl data, simulations and analysis scripts/fit-figures/29.pdf]

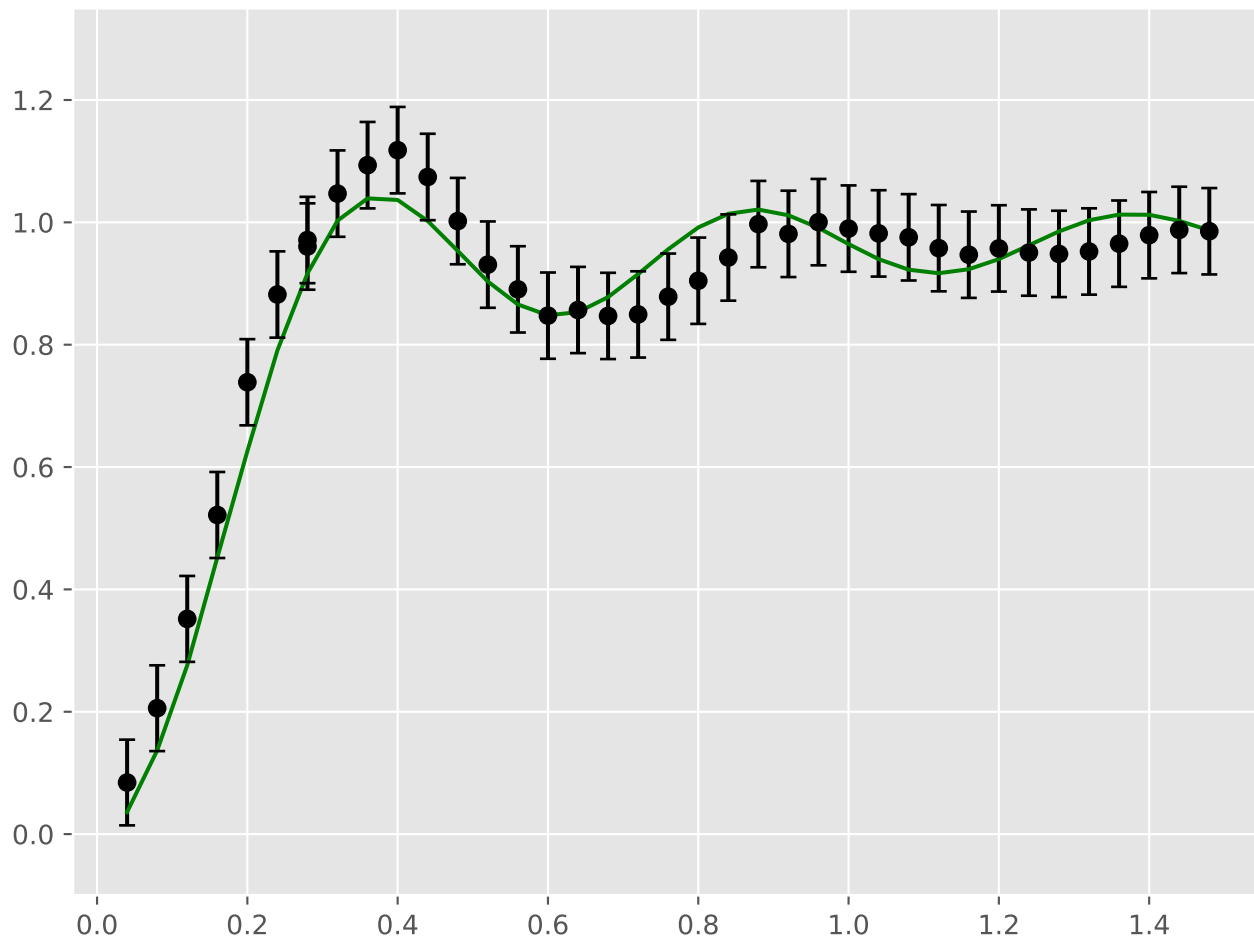

Supplement: Supplementary file 4 — Source data [file 41467_2022_29423_MOESM4_ESM.gz › source_data_2022/Figures_2B-E_SI5_SI6_SI7/Methyl dipolar-coupling measurements. Exptl data, simulations and analysis scripts/fit-figures/328.pdf]

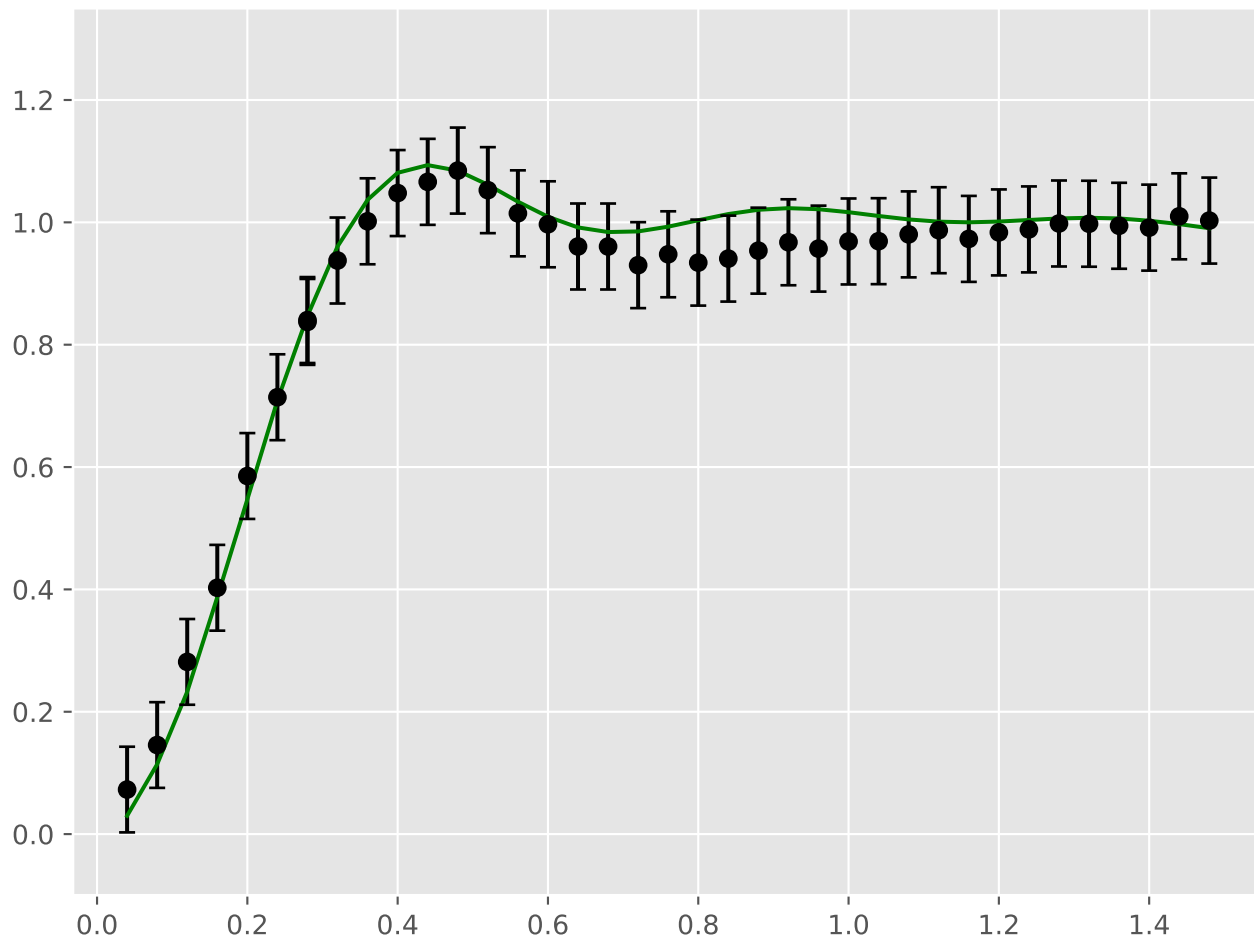

Supplement: Supplementary file 4 — Source data [file 41467_2022_29423_MOESM4_ESM.gz › source_data_2022/Figures_2B-E_SI5_SI6_SI7/Methyl dipolar-coupling measurements. Exptl data, simulations and analysis scripts/fit-figures/7.pdf]

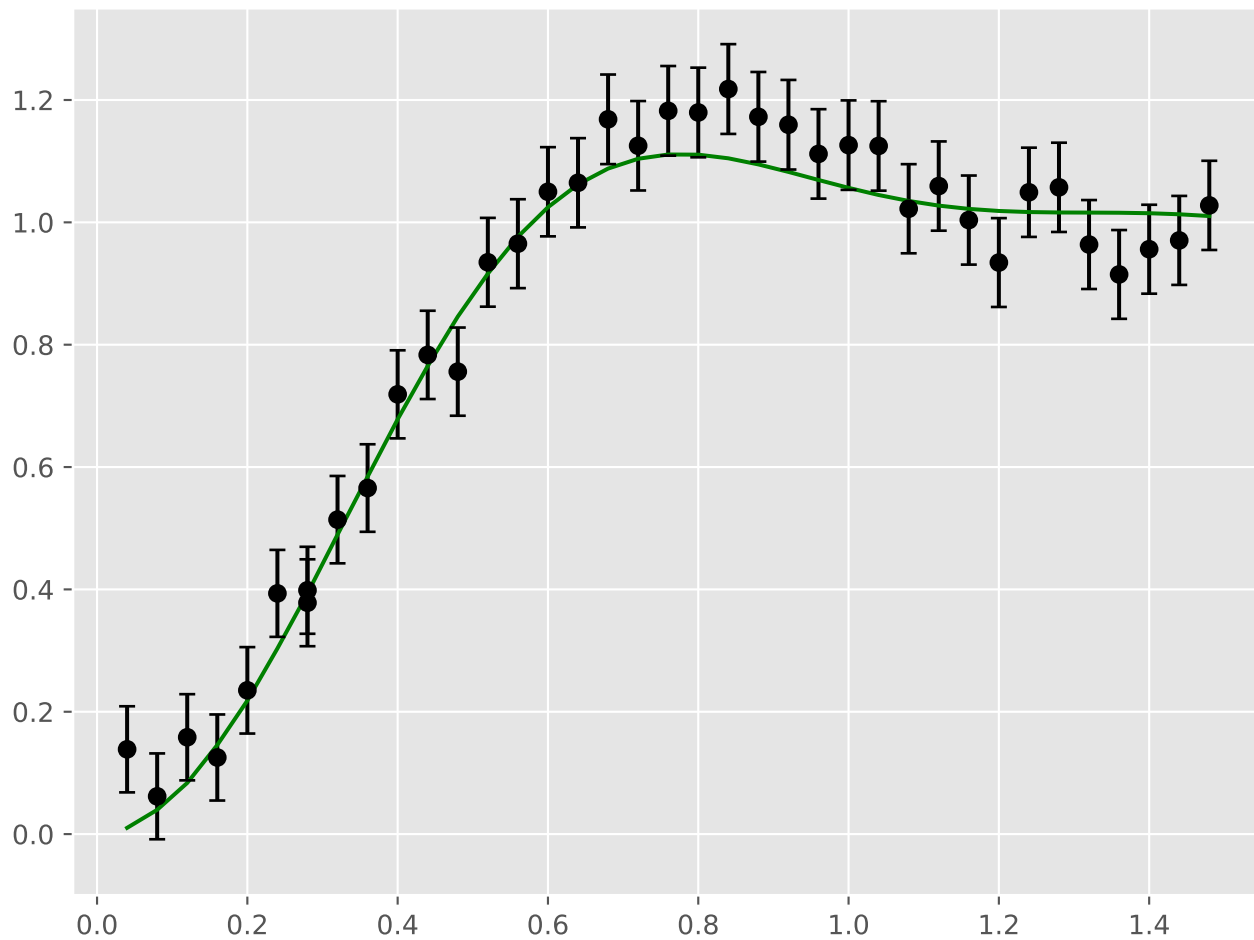

Supplement: Supplementary file 4 — Source data [file 41467_2022_29423_MOESM4_ESM.gz › source_data_2022/Figures_2B-E_SI5_SI6_SI7/Methyl dipolar-coupling measurements. Exptl data, simulations and analysis scripts/fit-figures/65.pdf]

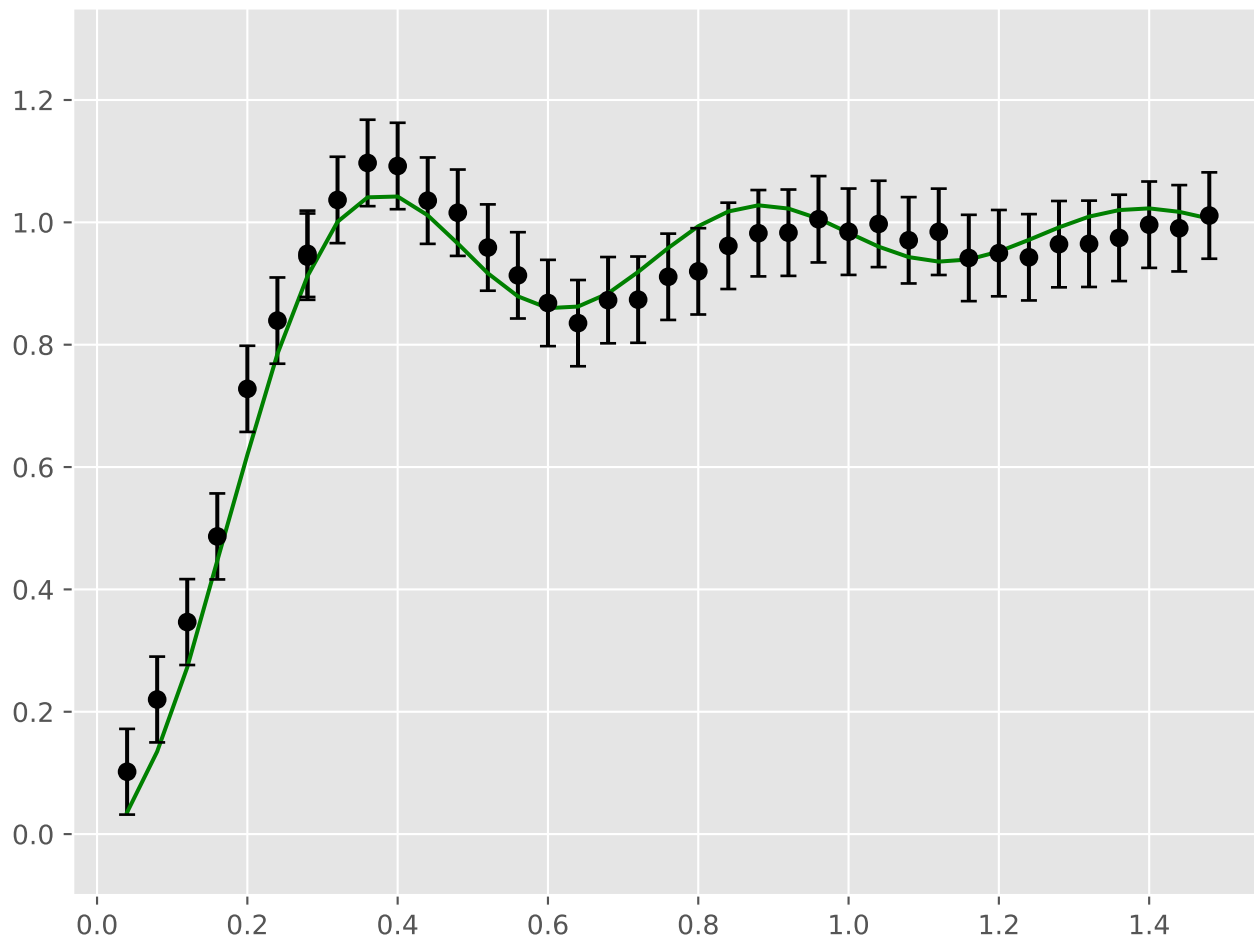

Supplement: Supplementary file 4 — Source data [file 41467_2022_29423_MOESM4_ESM.gz › source_data_2022/Figures_2B-E_SI5_SI6_SI7/Methyl dipolar-coupling measurements. Exptl data, simulations and analysis scripts/fit-figures/76.pdf]

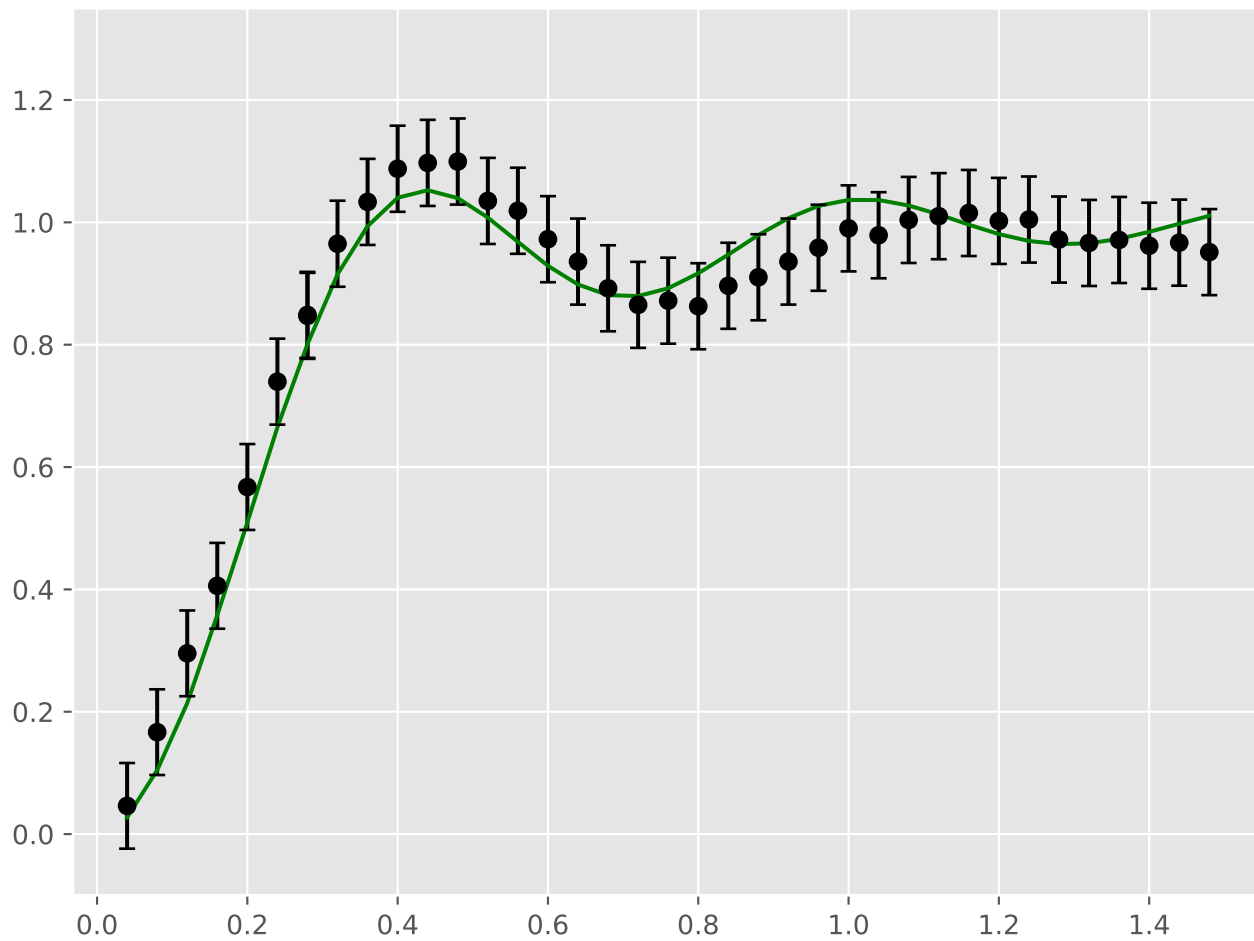

Supplement: Supplementary file 4 — Source data [file 41467_2022_29423_MOESM4_ESM.gz › source_data_2022/Figures_2B-E_SI5_SI6_SI7/Methyl dipolar-coupling measurements. Exptl data, simulations and analysis scripts/fit-figures/74.pdf]

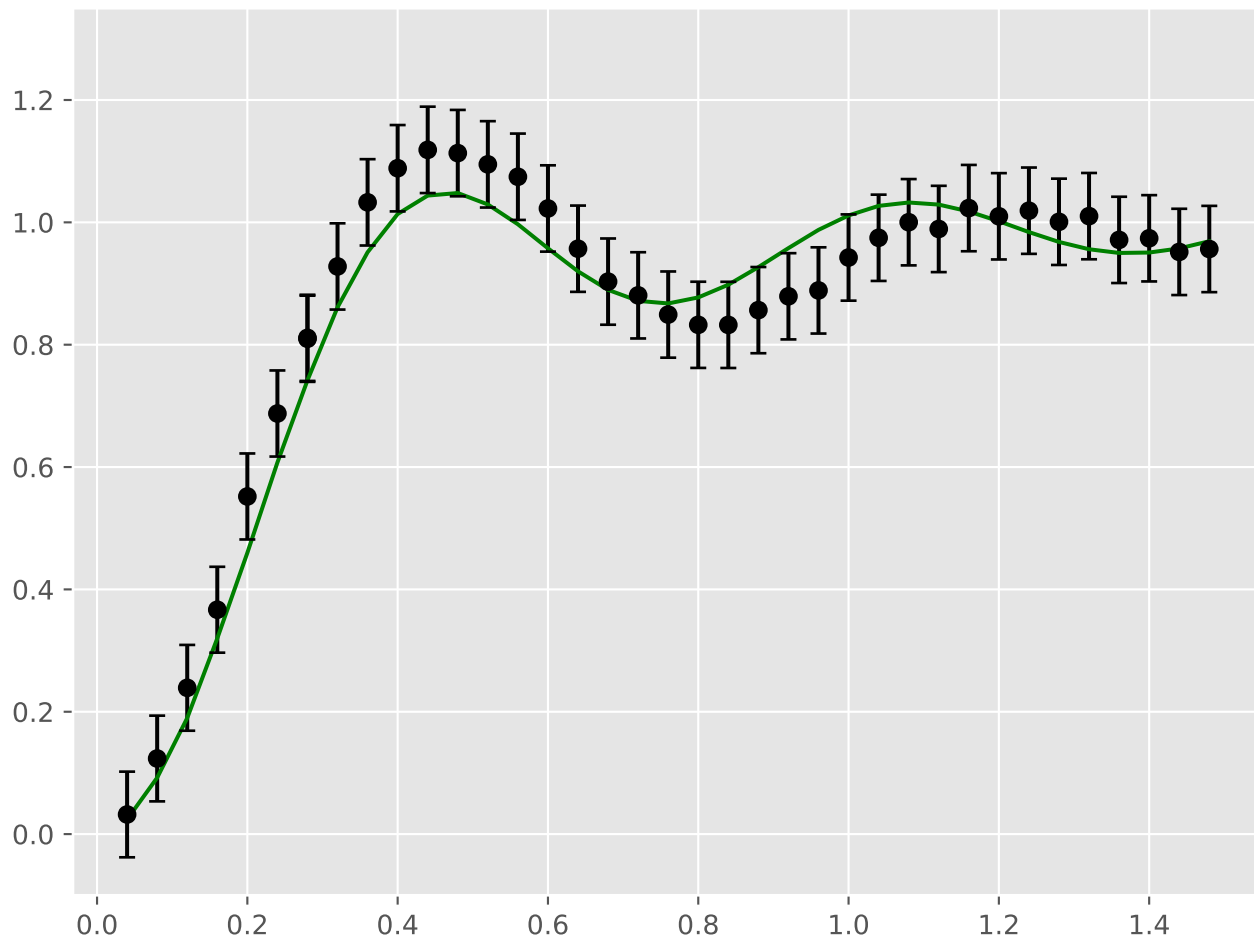

Supplement: Supplementary file 4 — Source data [file 41467_2022_29423_MOESM4_ESM.gz › source_data_2022/Figures_2B-E_SI5_SI6_SI7/Methyl dipolar-coupling measurements. Exptl data, simulations and analysis scripts/fit-figures/79.pdf]

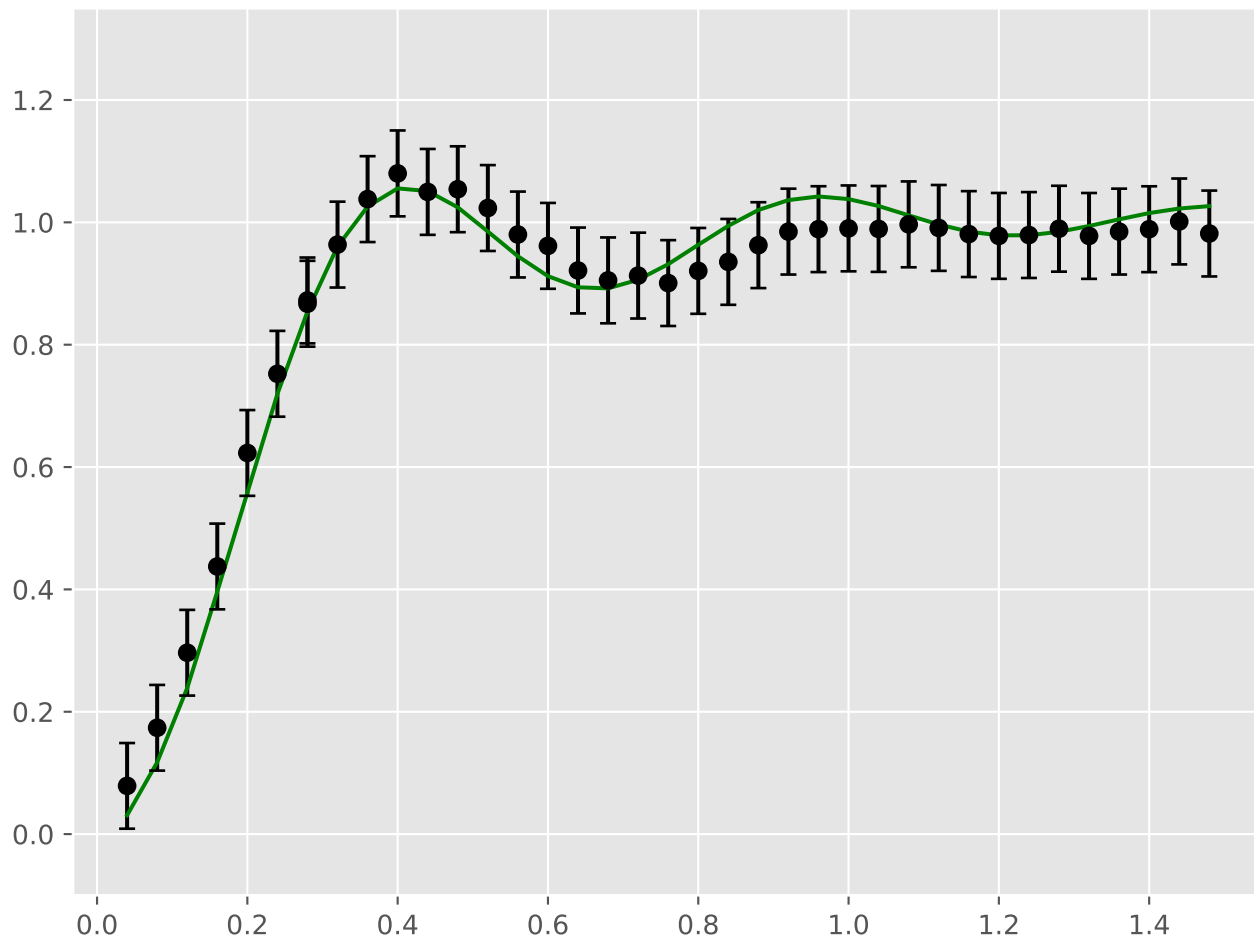

Supplement: Supplementary file 4 — Source data [file 41467_2022_29423_MOESM4_ESM.gz › source_data_2022/Figures_2B-E_SI5_SI6_SI7/Methyl dipolar-coupling measurements. Exptl data, simulations and analysis scripts/fit-figures/93.pdf]

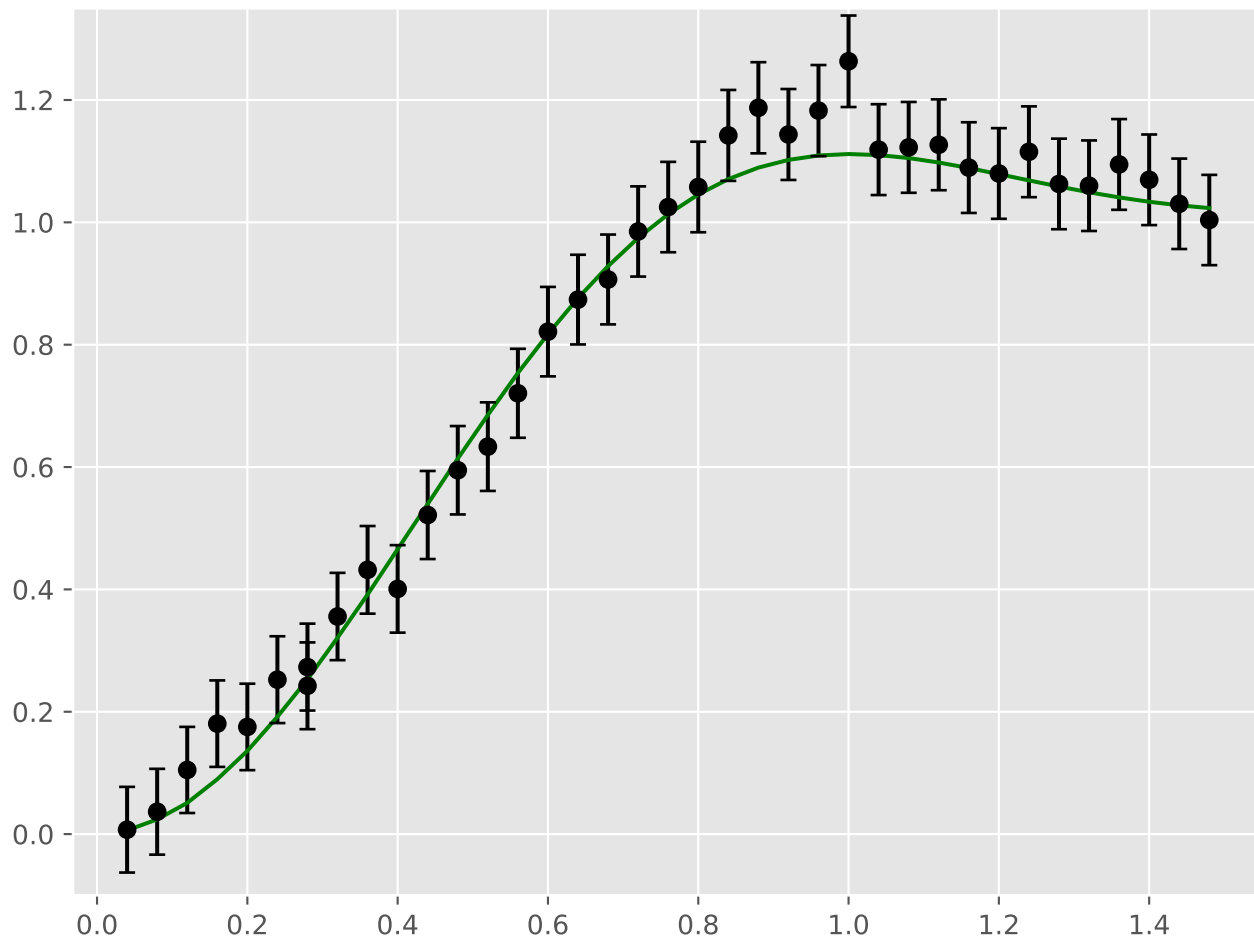

Supplement: Supplementary file 4 — Source data [file 41467_2022_29423_MOESM4_ESM.gz › source_data_2022/Figures_2B-E_SI5_SI6_SI7/Methyl dipolar-coupling measurements. Exptl data, simulations and analysis scripts/fit-figures/99.pdf]

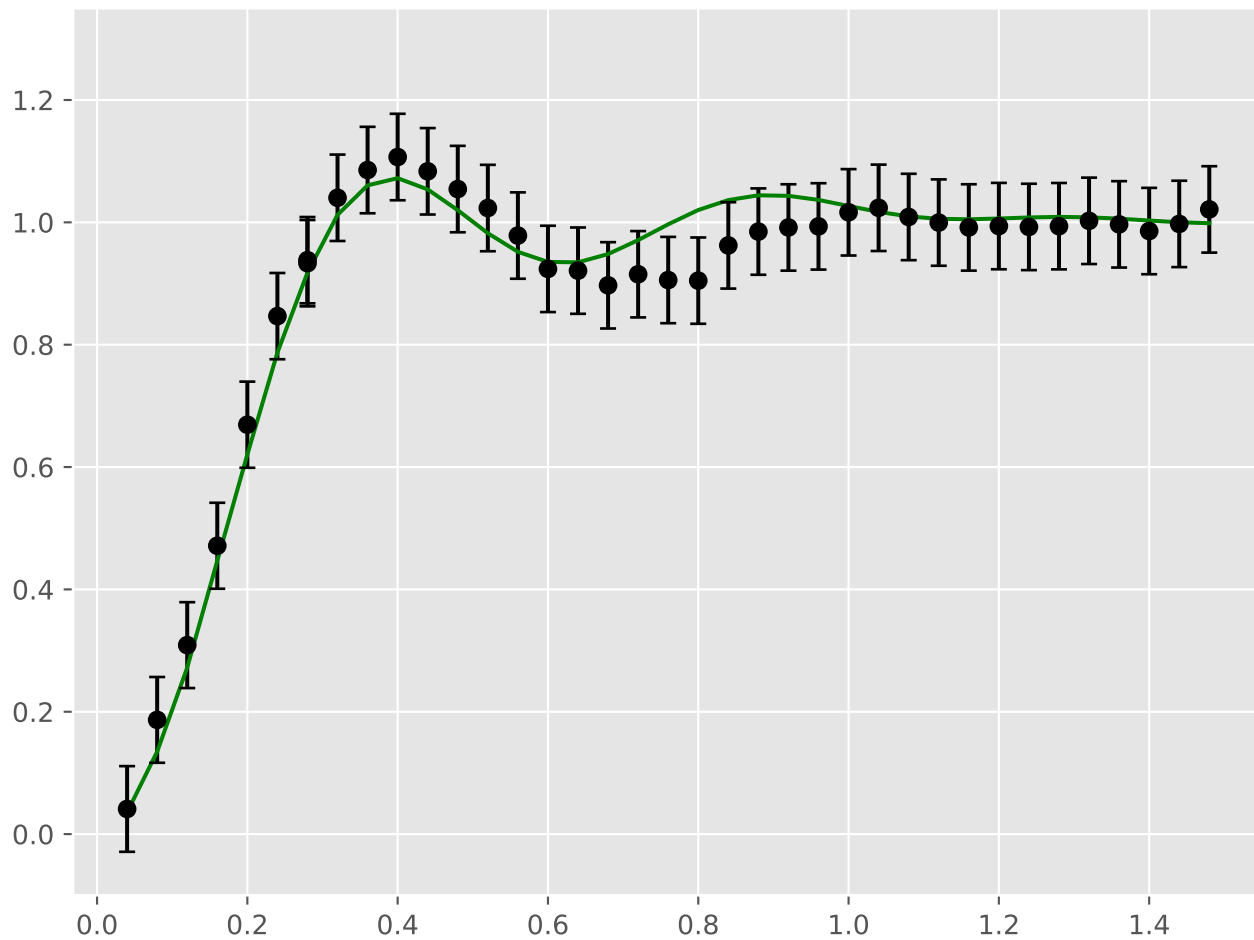

Supplement: Supplementary file 4 — Source data [file 41467_2022_29423_MOESM4_ESM.gz › source_data_2022/Figures_2B-E_SI5_SI6_SI7/Methyl dipolar-coupling measurements. Exptl data, simulations and analysis scripts/fit-figures/87.pdf]

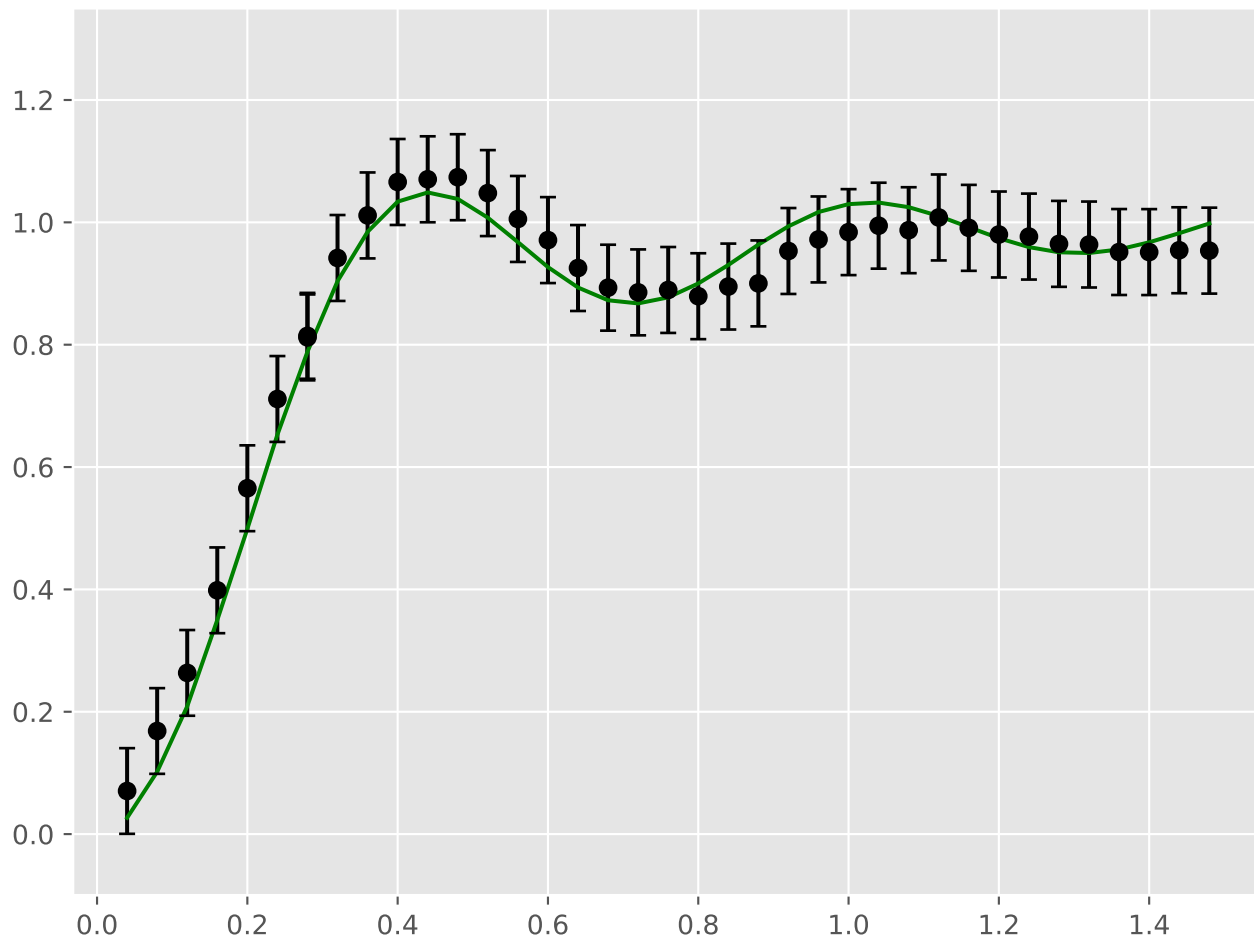

Supplement: Supplementary file 4 — Source data [file 41467_2022_29423_MOESM4_ESM.gz › source_data_2022/Figures_2B-E_SI5_SI6_SI7/Methyl dipolar-coupling measurements. Exptl data, simulations and analysis scripts/fit-figures/204.pdf]

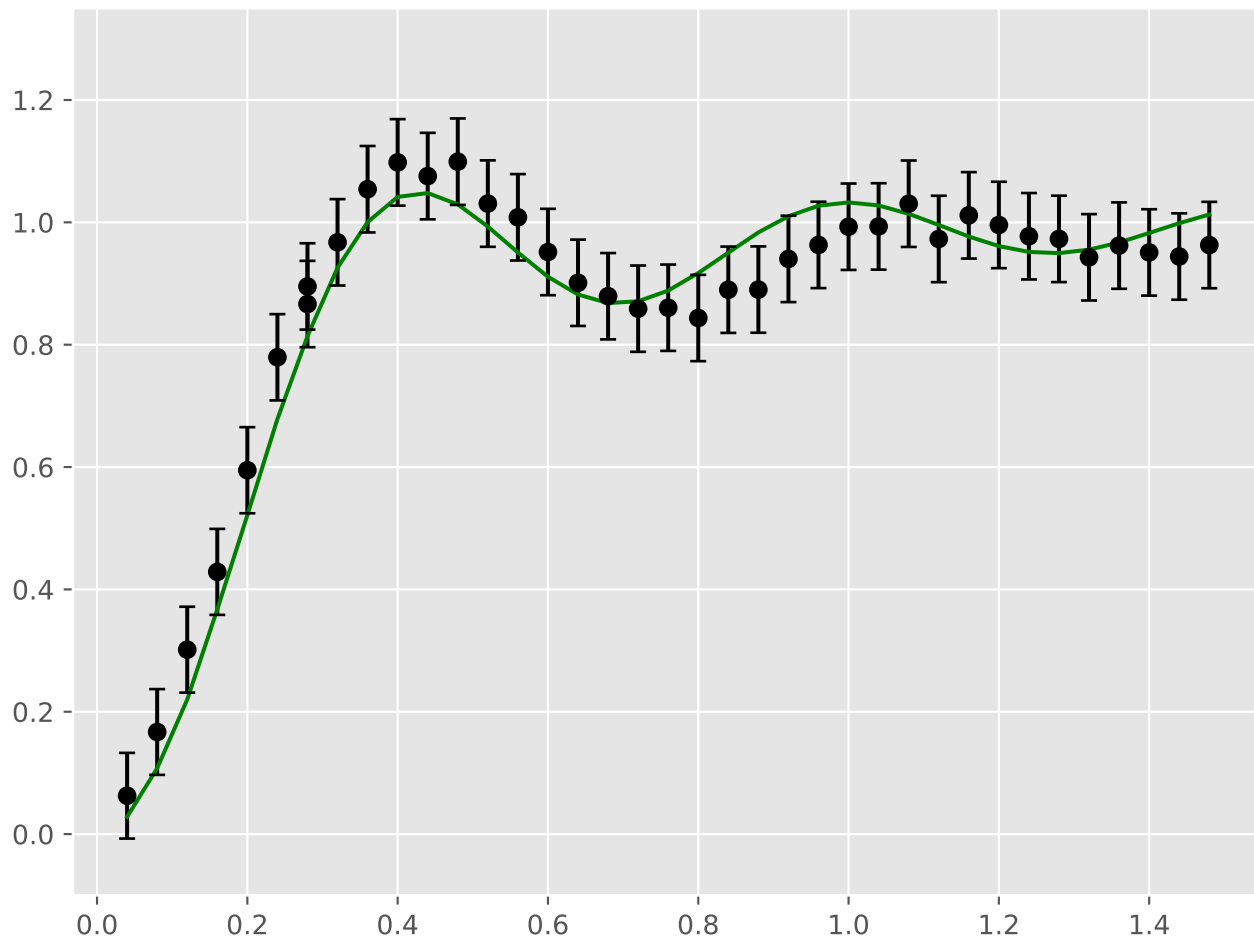

Supplement: Supplementary file 4 — Source data [file 41467_2022_29423_MOESM4_ESM.gz › source_data_2022/Figures_2B-E_SI5_SI6_SI7/Methyl dipolar-coupling measurements. Exptl data, simulations and analysis scripts/fit-figures/236.pdf]

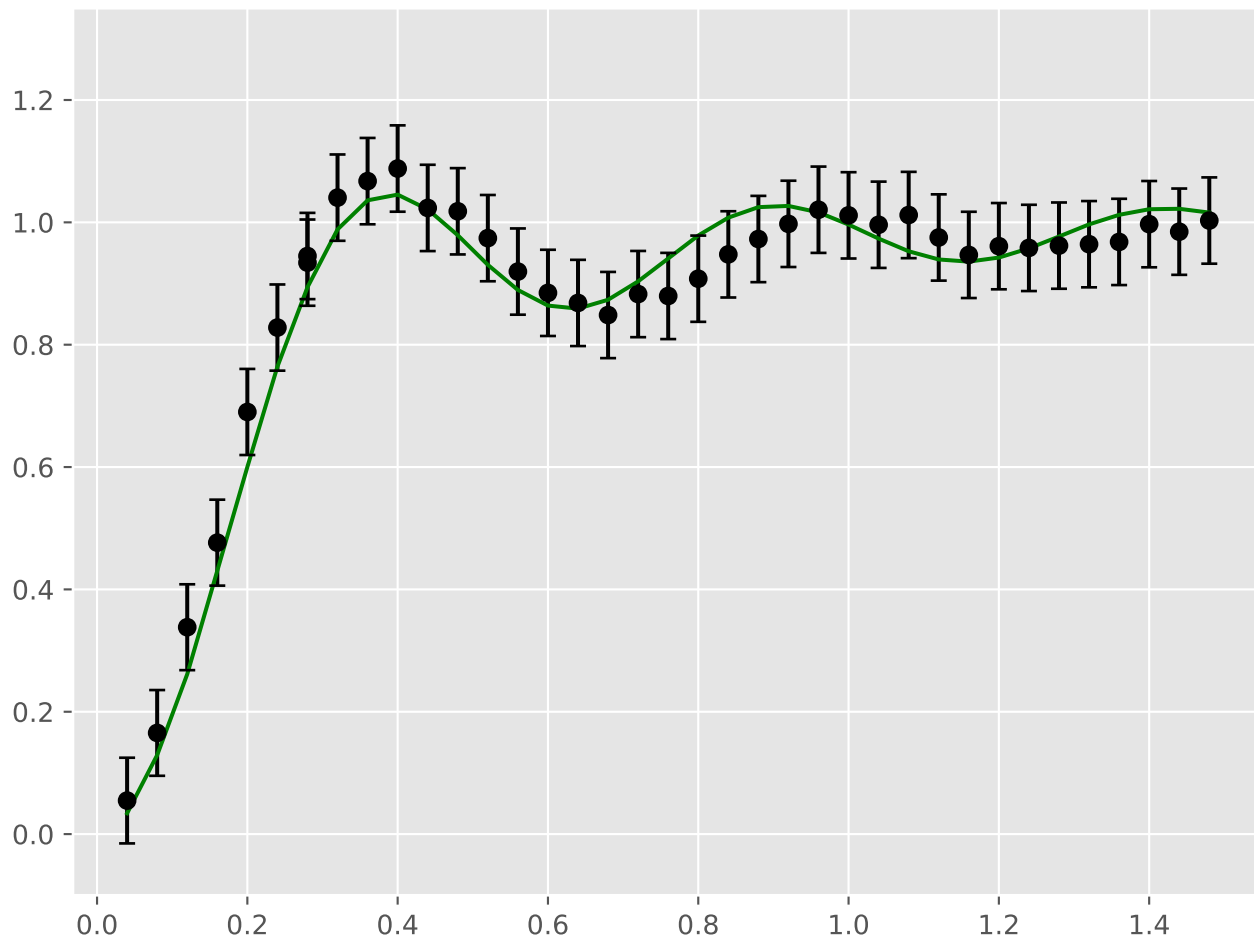

Supplement: Supplementary file 4 — Source data [file 41467_2022_29423_MOESM4_ESM.gz › source_data_2022/Figures_2B-E_SI5_SI6_SI7/Methyl dipolar-coupling measurements. Exptl data, simulations and analysis scripts/fit-figures/251.pdf]

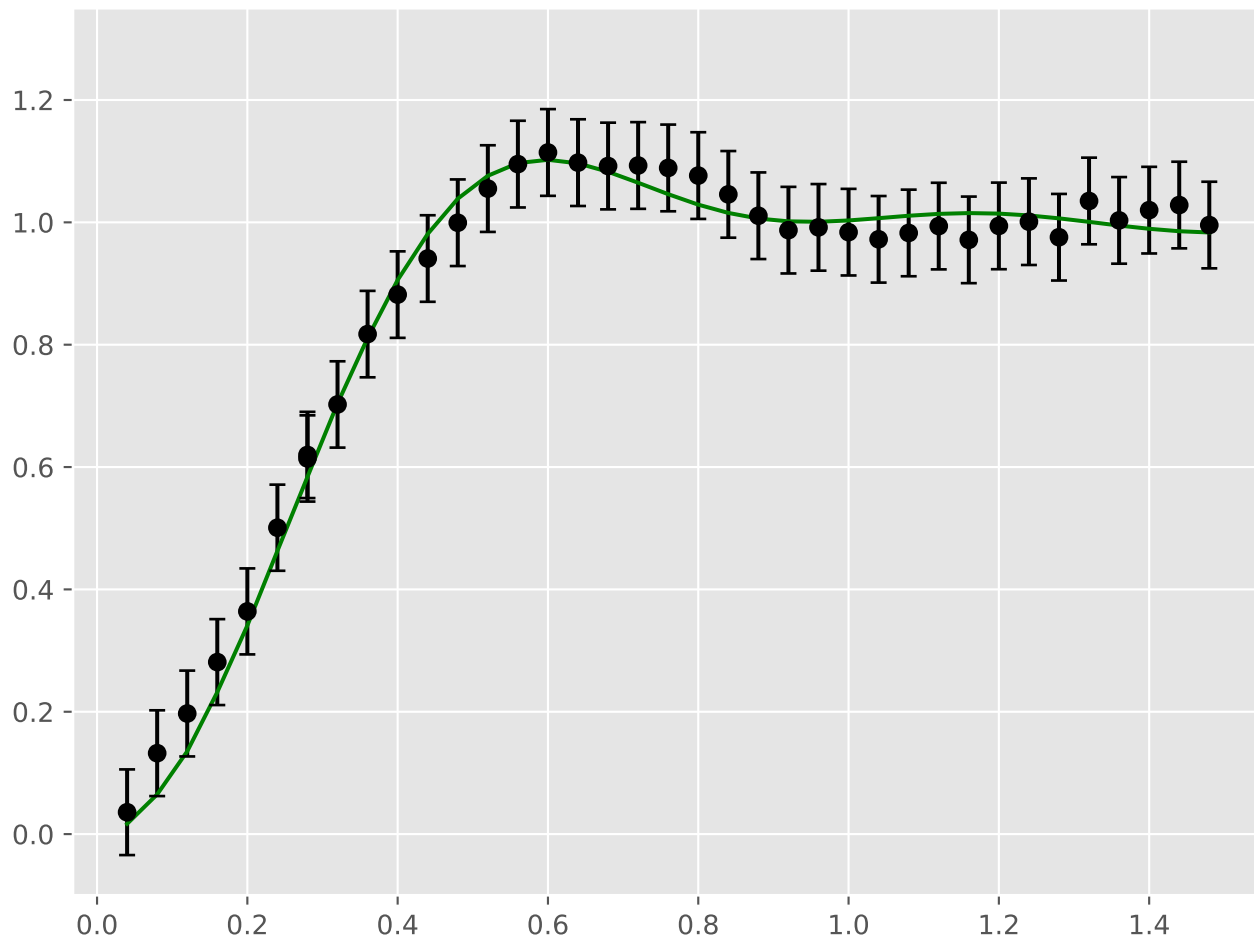

Supplement: Supplementary file 4 — Source data [file 41467_2022_29423_MOESM4_ESM.gz › source_data_2022/Figures_2B-E_SI5_SI6_SI7/Methyl dipolar-coupling measurements. Exptl data, simulations and analysis scripts/fit-figures/238.pdf]

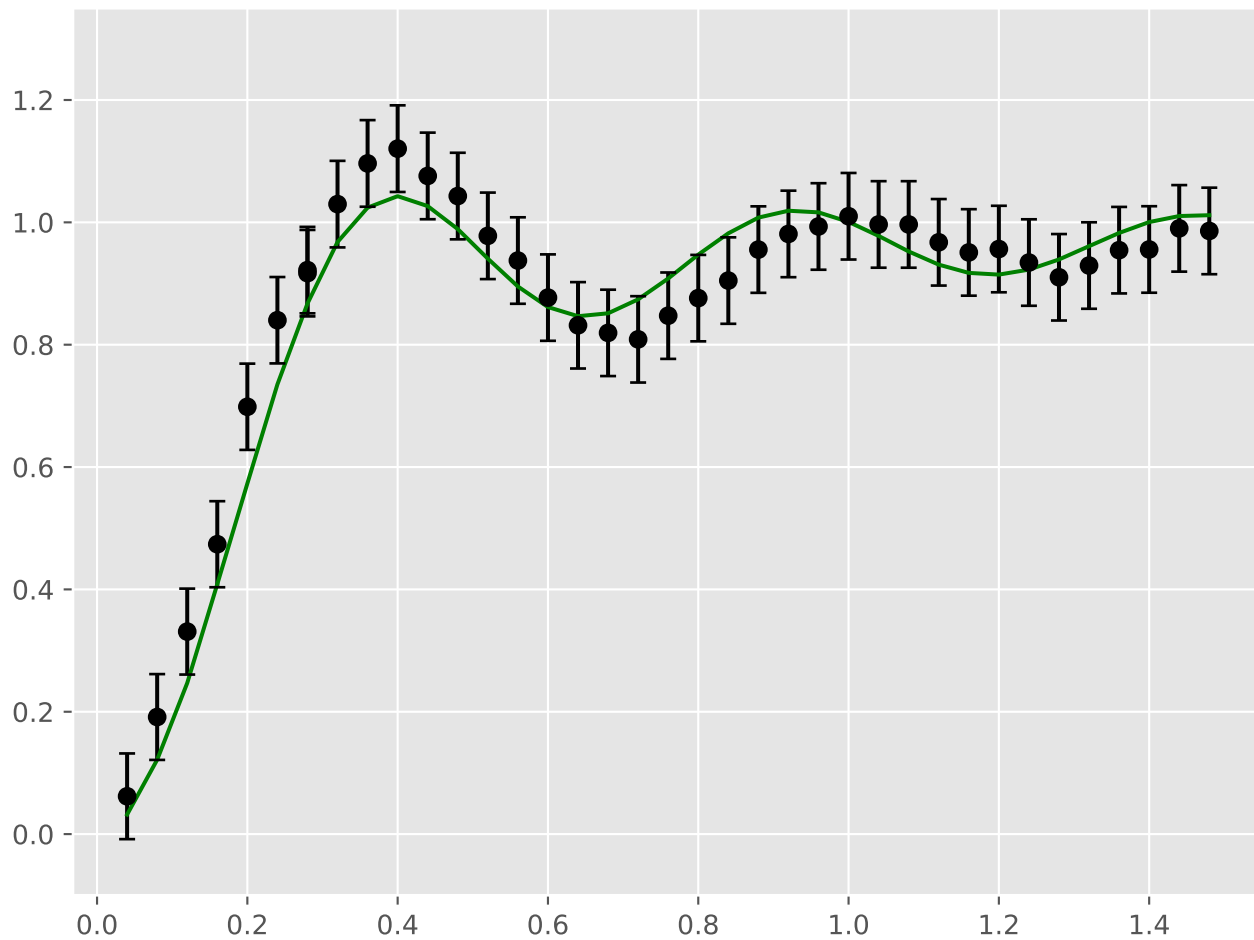

Supplement: Supplementary file 4 — Source data [file 41467_2022_29423_MOESM4_ESM.gz › source_data_2022/Figures_2B-E_SI5_SI6_SI7/Methyl dipolar-coupling measurements. Exptl data, simulations and analysis scripts/fit-figures/340.pdf]

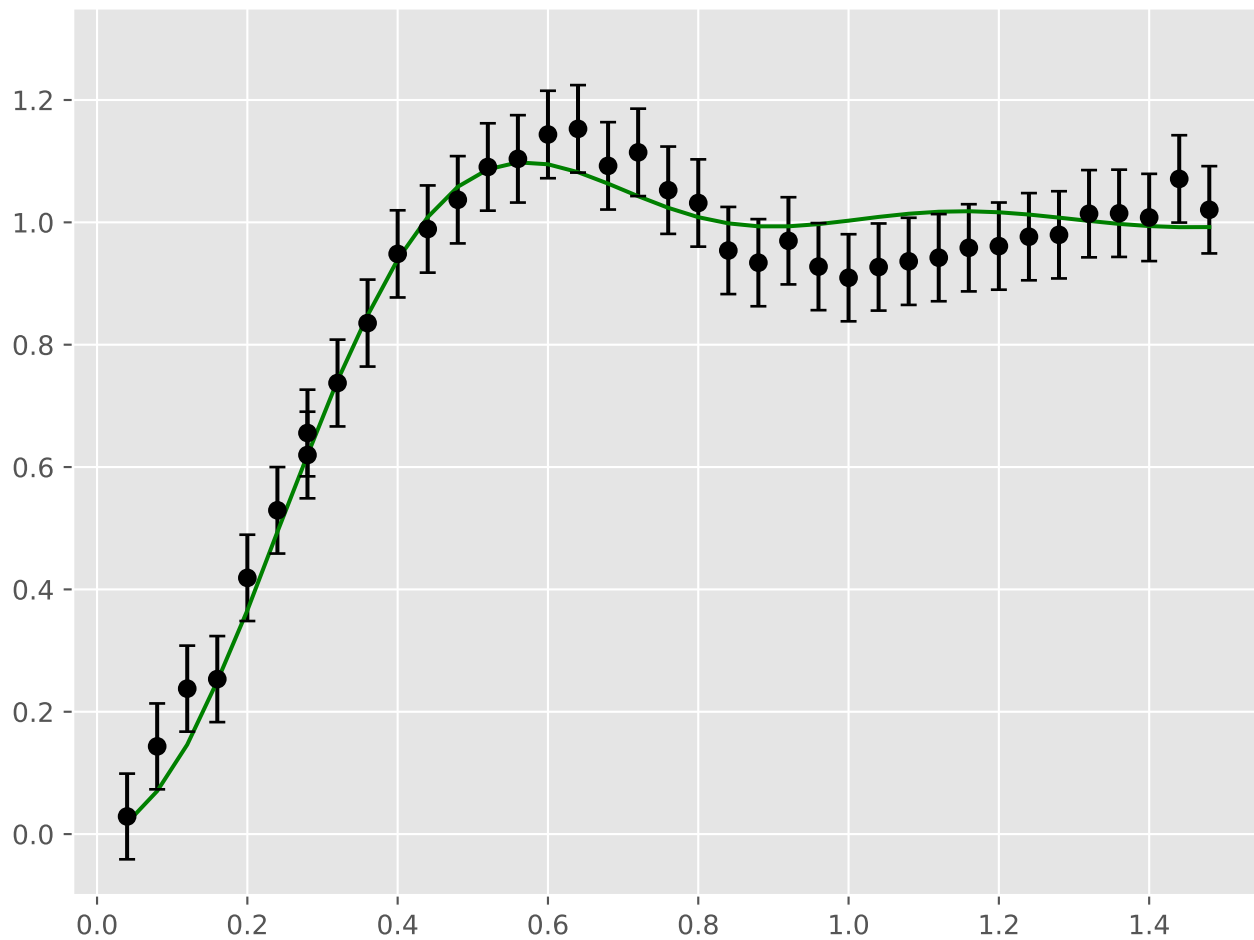

Supplement: Supplementary file 4 — Source data [file 41467_2022_29423_MOESM4_ESM.gz › source_data_2022/Figures_2B-E_SI5_SI6_SI7/Methyl dipolar-coupling measurements. Exptl data, simulations and analysis scripts/fit-figures/345.pdf]

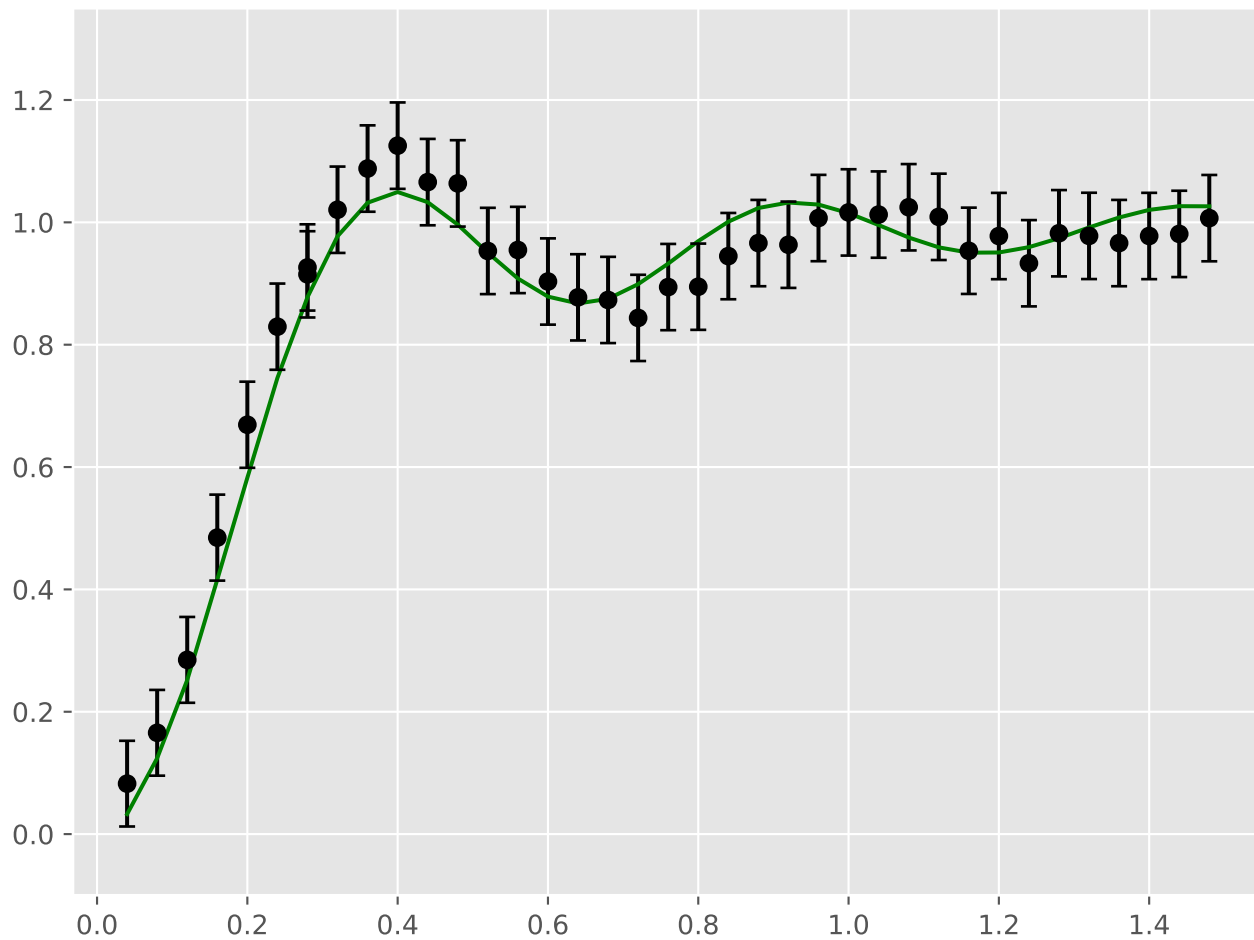

Supplement: Supplementary file 4 — Source data [file 41467_2022_29423_MOESM4_ESM.gz › source_data_2022/Figures_2B-E_SI5_SI6_SI7/Methyl dipolar-coupling measurements. Exptl data, simulations and analysis scripts/fit-figures/274.pdf]

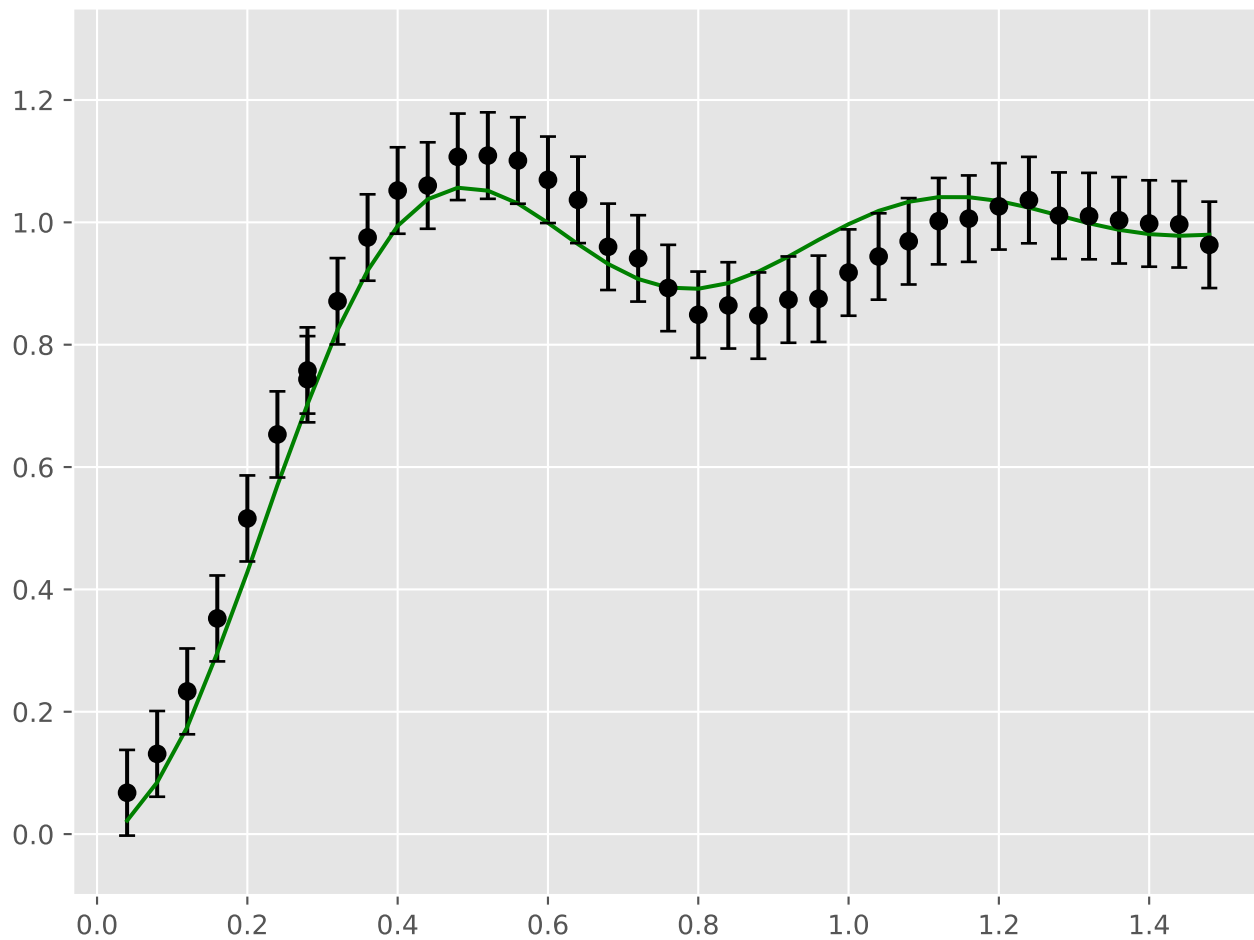

Supplement: Supplementary file 4 — Source data [file 41467_2022_29423_MOESM4_ESM.gz › source_data_2022/Figures_2B-E_SI5_SI6_SI7/Methyl dipolar-coupling measurements. Exptl data, simulations and analysis scripts/fit-figures/277.pdf]

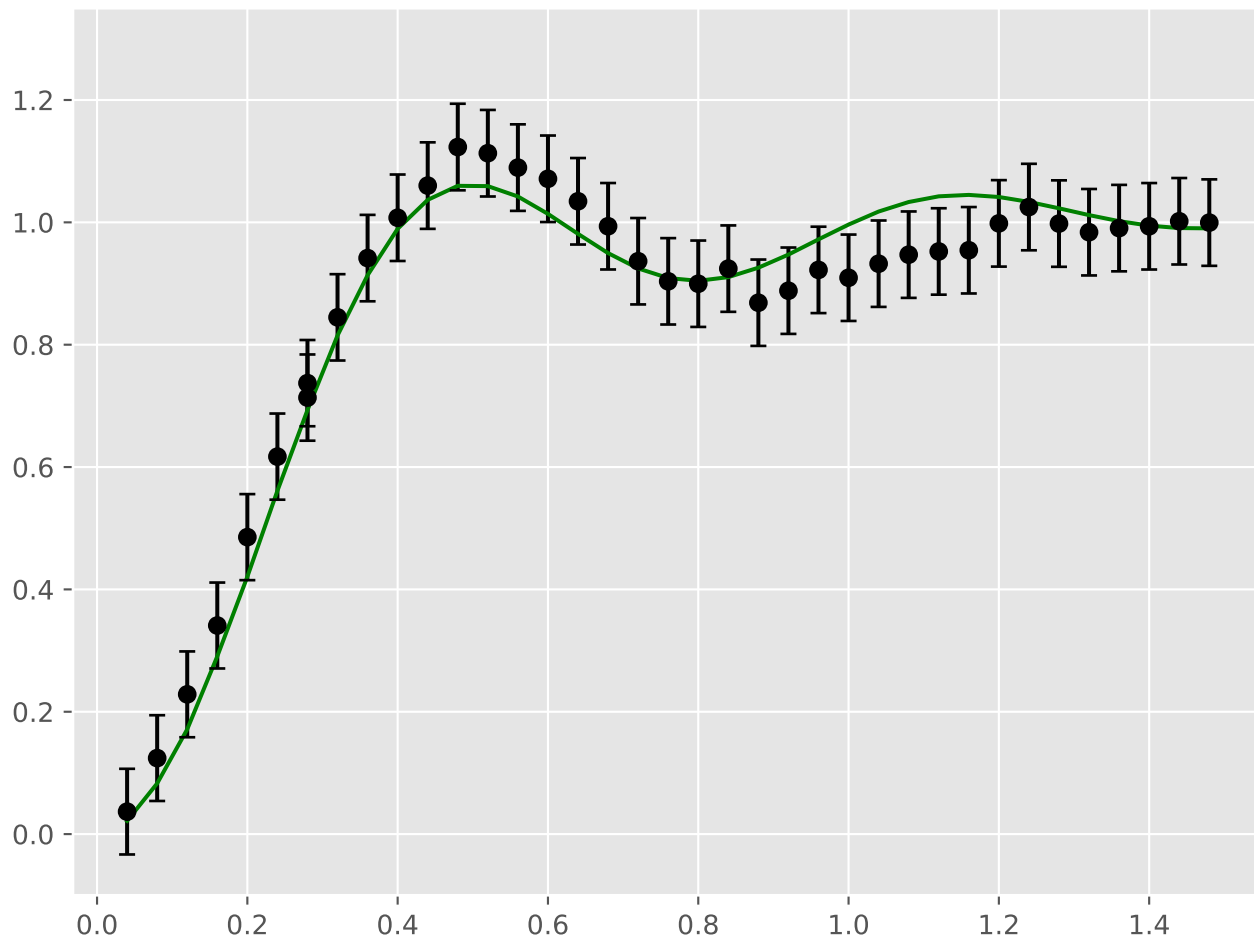

Supplement: Supplementary file 4 — Source data [file 41467_2022_29423_MOESM4_ESM.gz › source_data_2022/Figures_2B-E_SI5_SI6_SI7/Methyl dipolar-coupling measurements. Exptl data, simulations and analysis scripts/fit-figures/242.pdf]

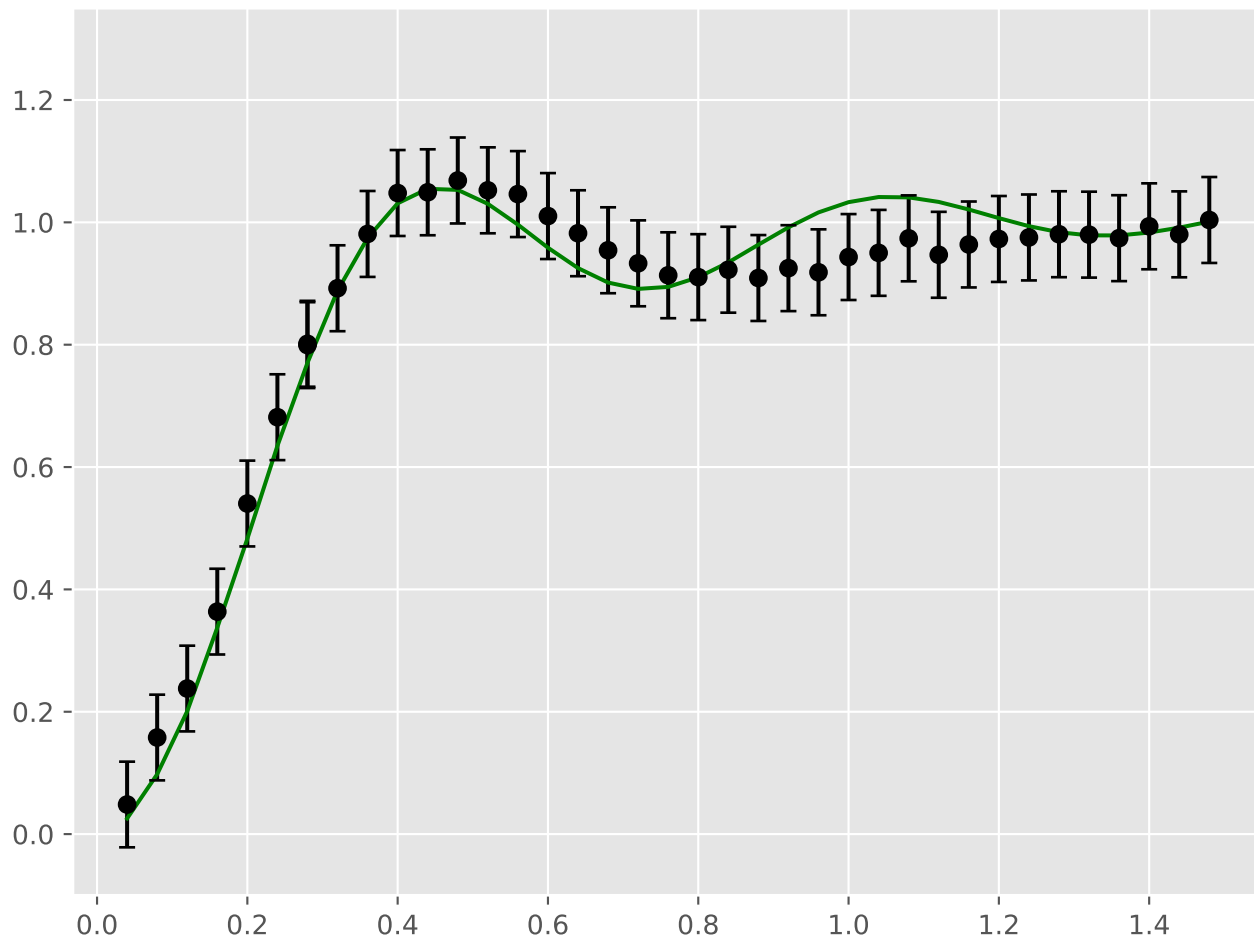

Supplement: Supplementary file 4 — Source data [file 41467_2022_29423_MOESM4_ESM.gz › source_data_2022/Figures_2B-E_SI5_SI6_SI7/Methyl dipolar-coupling measurements. Exptl data, simulations and analysis scripts/fit-figures/53.pdf]

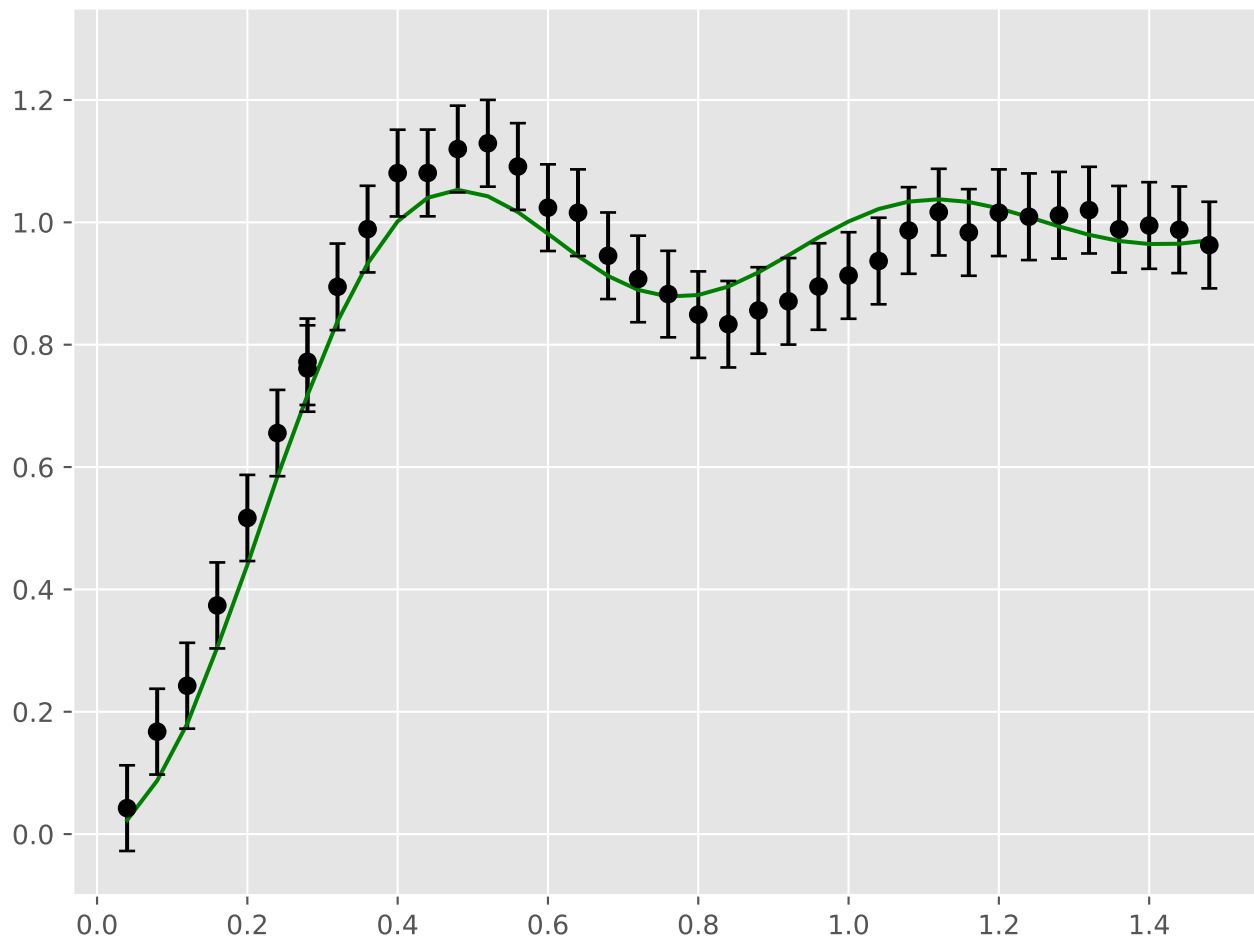

Supplement: Supplementary file 4 — Source data [file 41467_2022_29423_MOESM4_ESM.gz › source_data_2022/Figures_2B-E_SI5_SI6_SI7/Methyl dipolar-coupling measurements. Exptl data, simulations and analysis scripts/fit-figures/273.pdf]

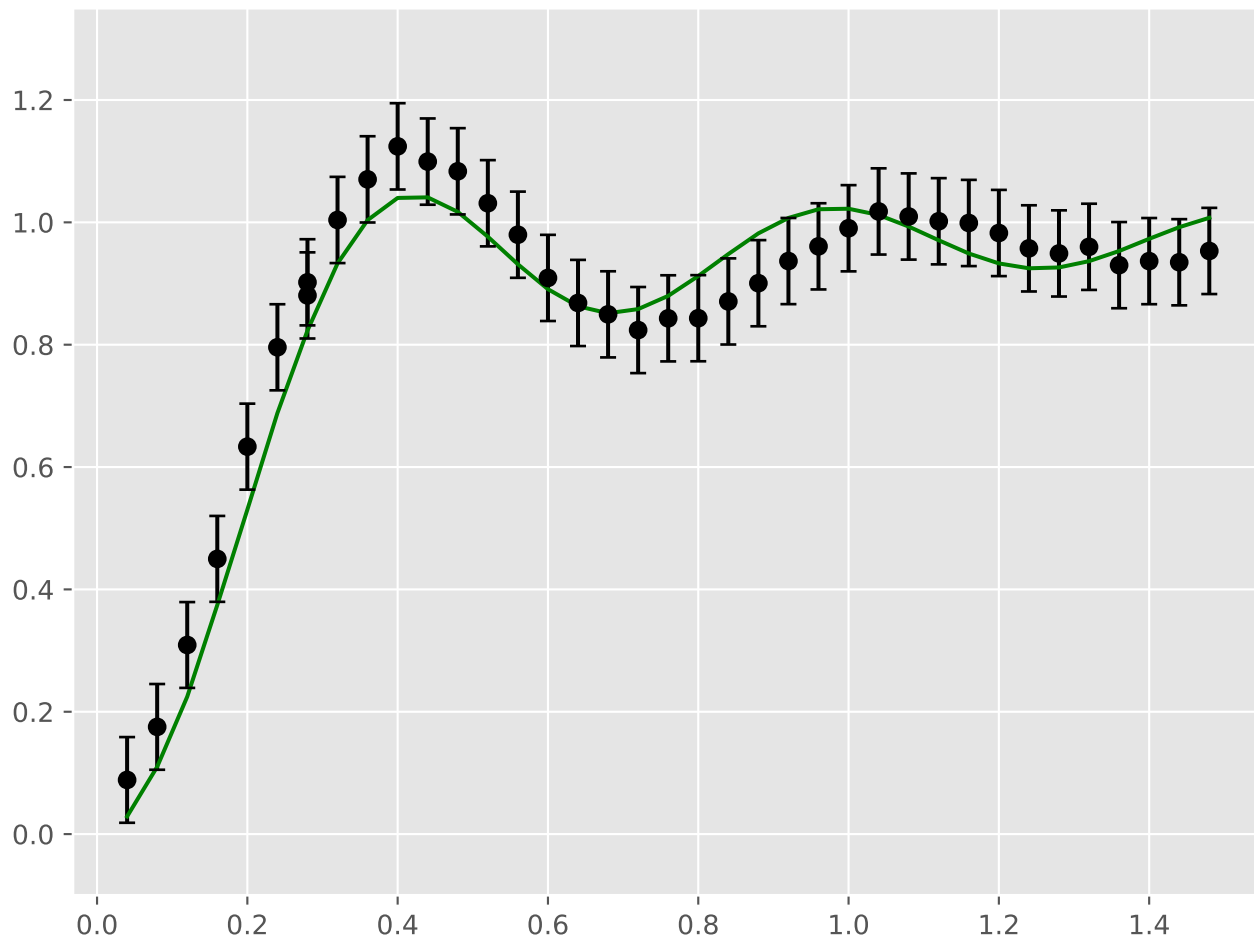

Supplement: Supplementary file 4 — Source data [file 41467_2022_29423_MOESM4_ESM.gz › source_data_2022/Figures_2B-E_SI5_SI6_SI7/Methyl dipolar-coupling measurements. Exptl data, simulations and analysis scripts/fit-figures/286.pdf]

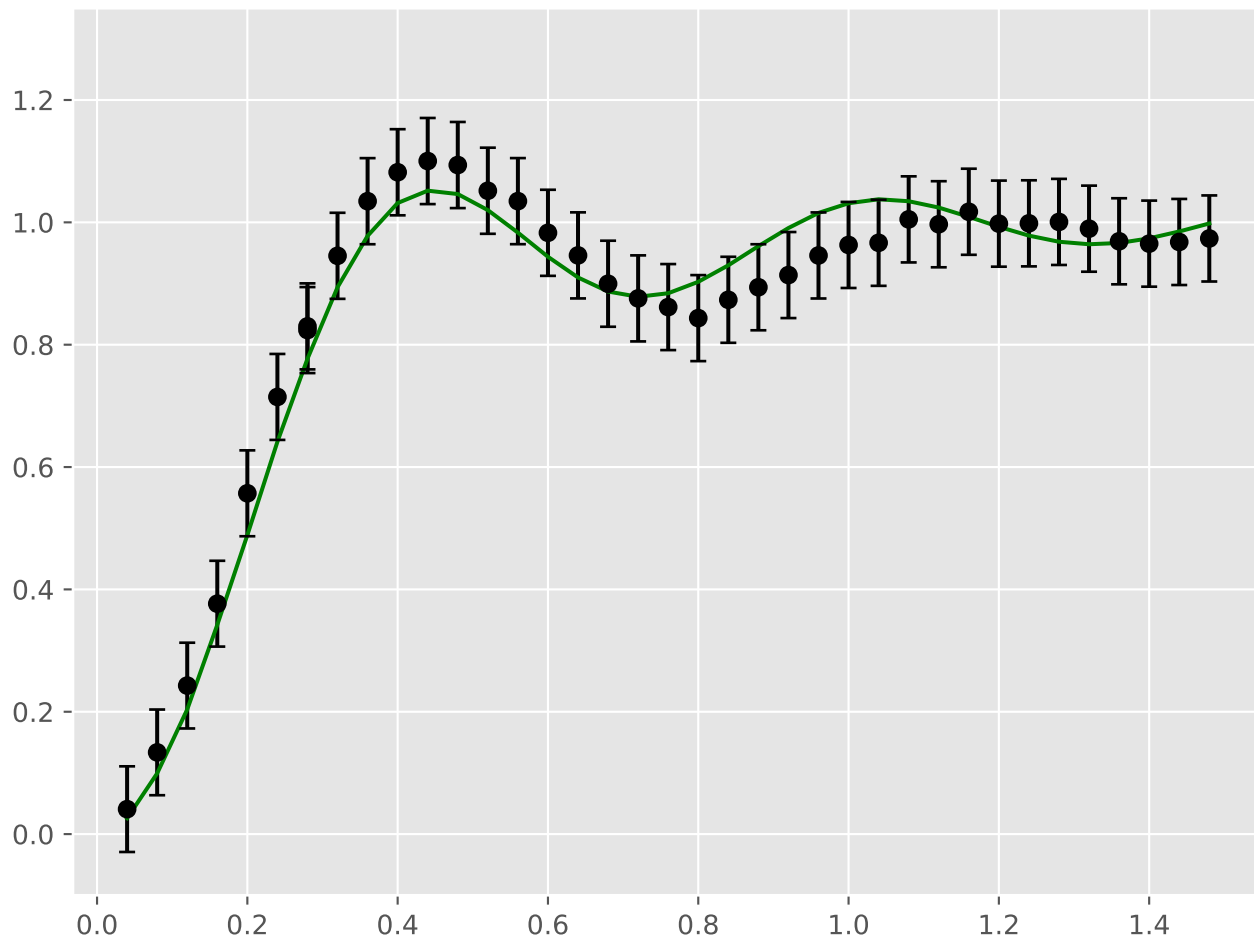

Supplement: Supplementary file 4 — Source data [file 41467_2022_29423_MOESM4_ESM.gz › source_data_2022/Figures_2B-E_SI5_SI6_SI7/Methyl dipolar-coupling measurements. Exptl data, simulations and analysis scripts/fit-figures/290.pdf]

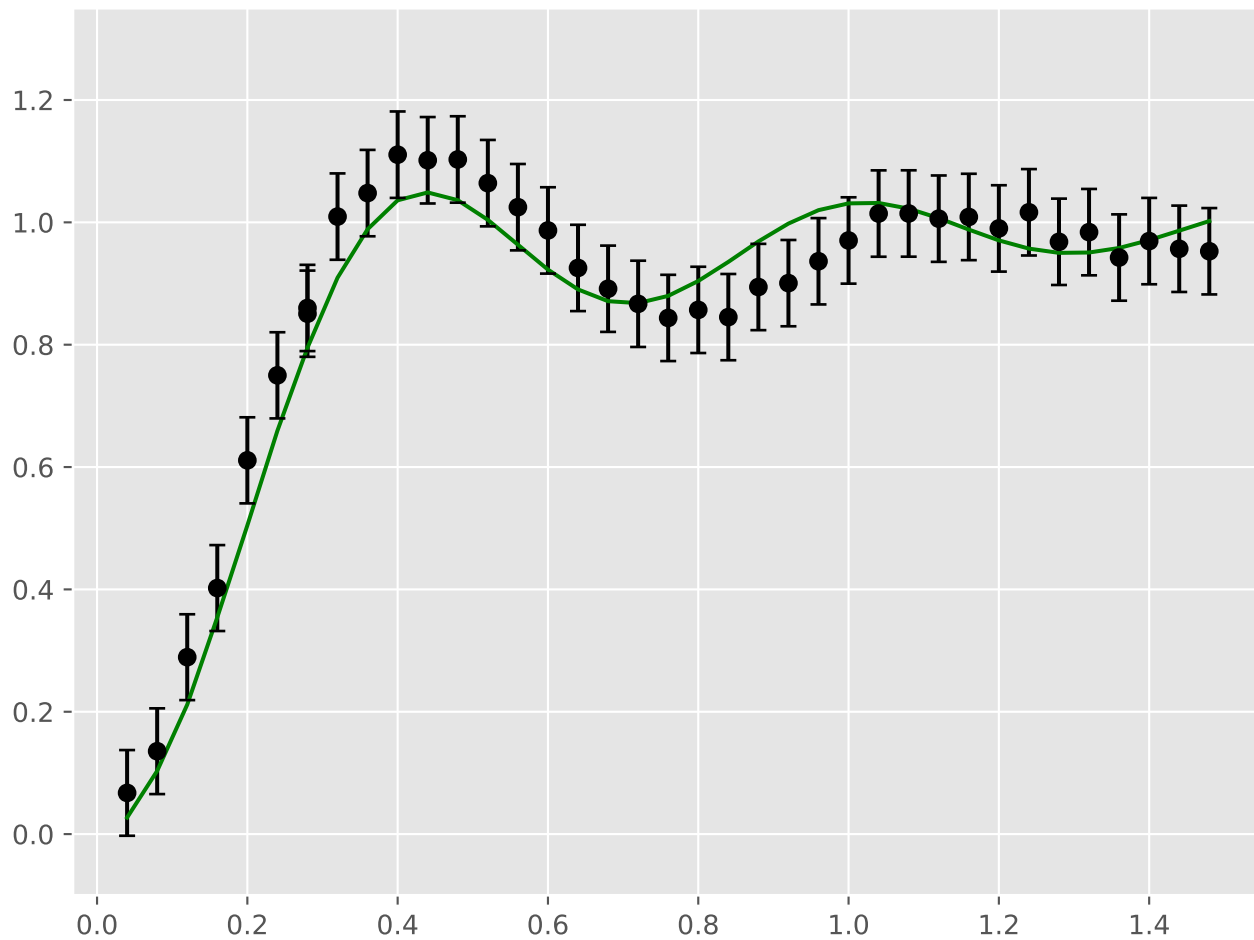

Supplement: Supplementary file 4 — Source data [file 41467_2022_29423_MOESM4_ESM.gz › source_data_2022/Figures_2B-E_SI5_SI6_SI7/Methyl dipolar-coupling measurements. Exptl data, simulations and analysis scripts/fit-figures/292.pdf]

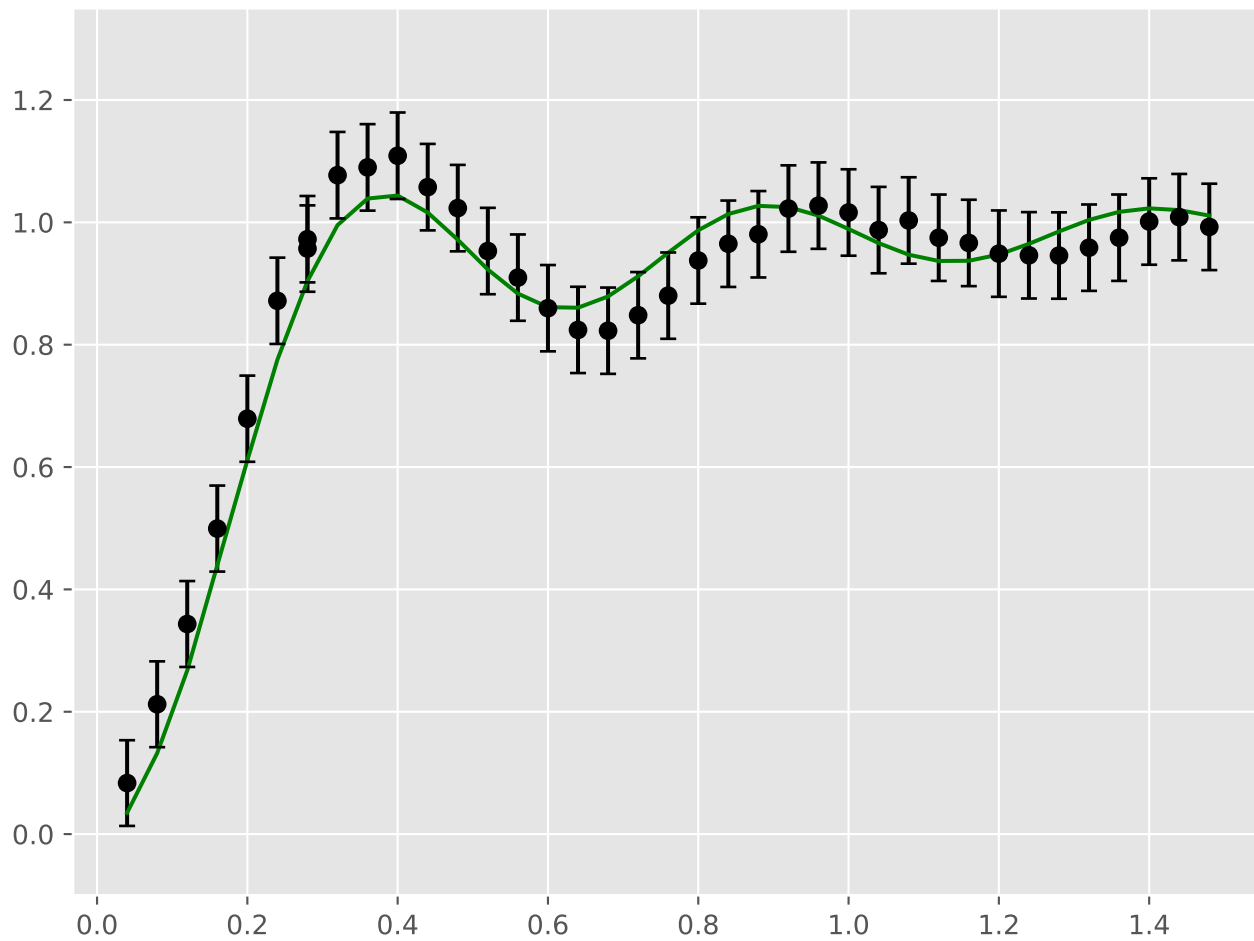

Supplement: Supplementary file 4 — Source data [file 41467_2022_29423_MOESM4_ESM.gz › source_data_2022/Figures_2B-E_SI5_SI6_SI7/Methyl dipolar-coupling measurements. Exptl data, simulations and analysis scripts/fit-figures/310.pdf]

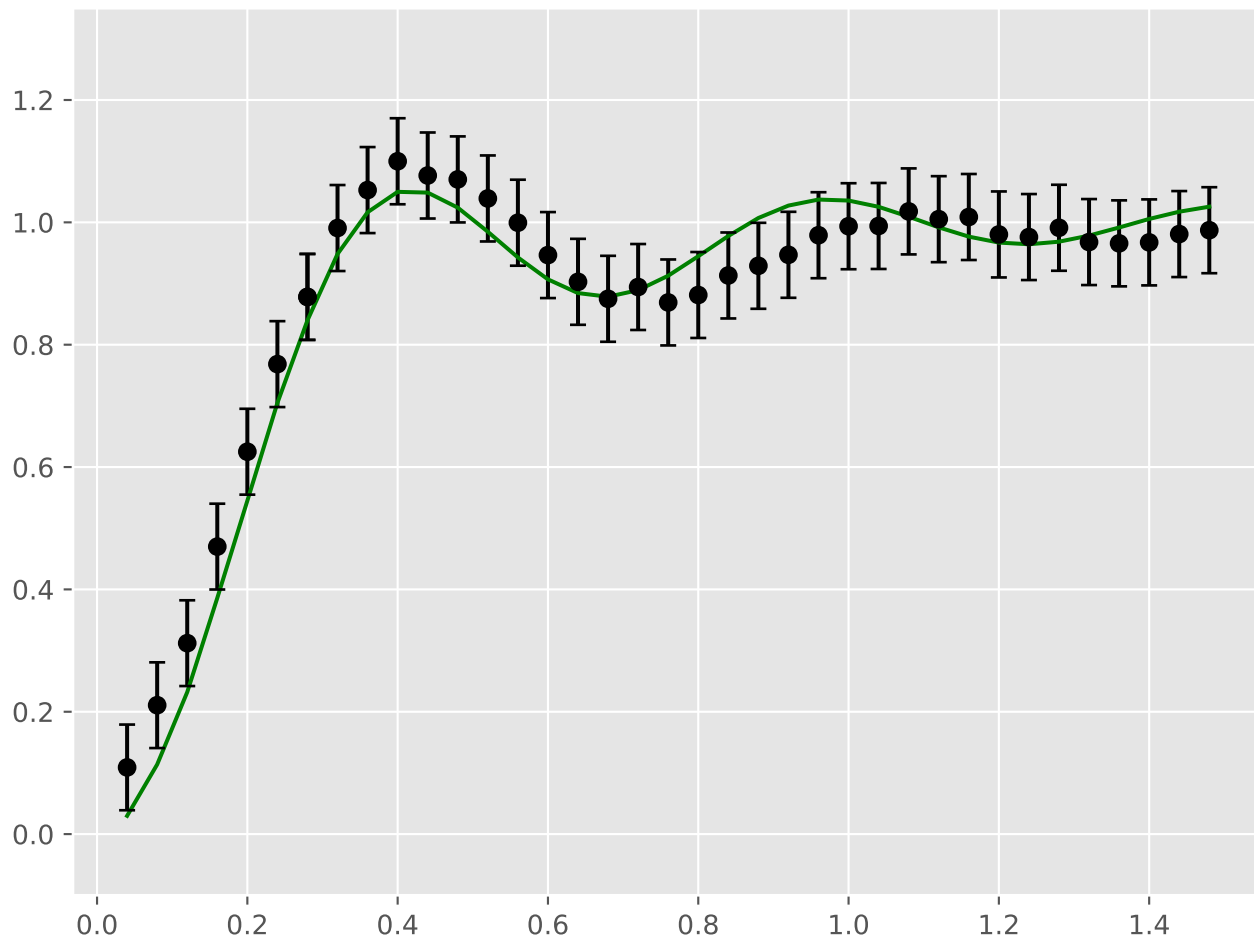

Supplement: Supplementary file 4 — Source data [file 41467_2022_29423_MOESM4_ESM.gz › source_data_2022/Figures_2B-E_SI5_SI6_SI7/Methyl dipolar-coupling measurements. Exptl data, simulations and analysis scripts/fit-figures/32.pdf]

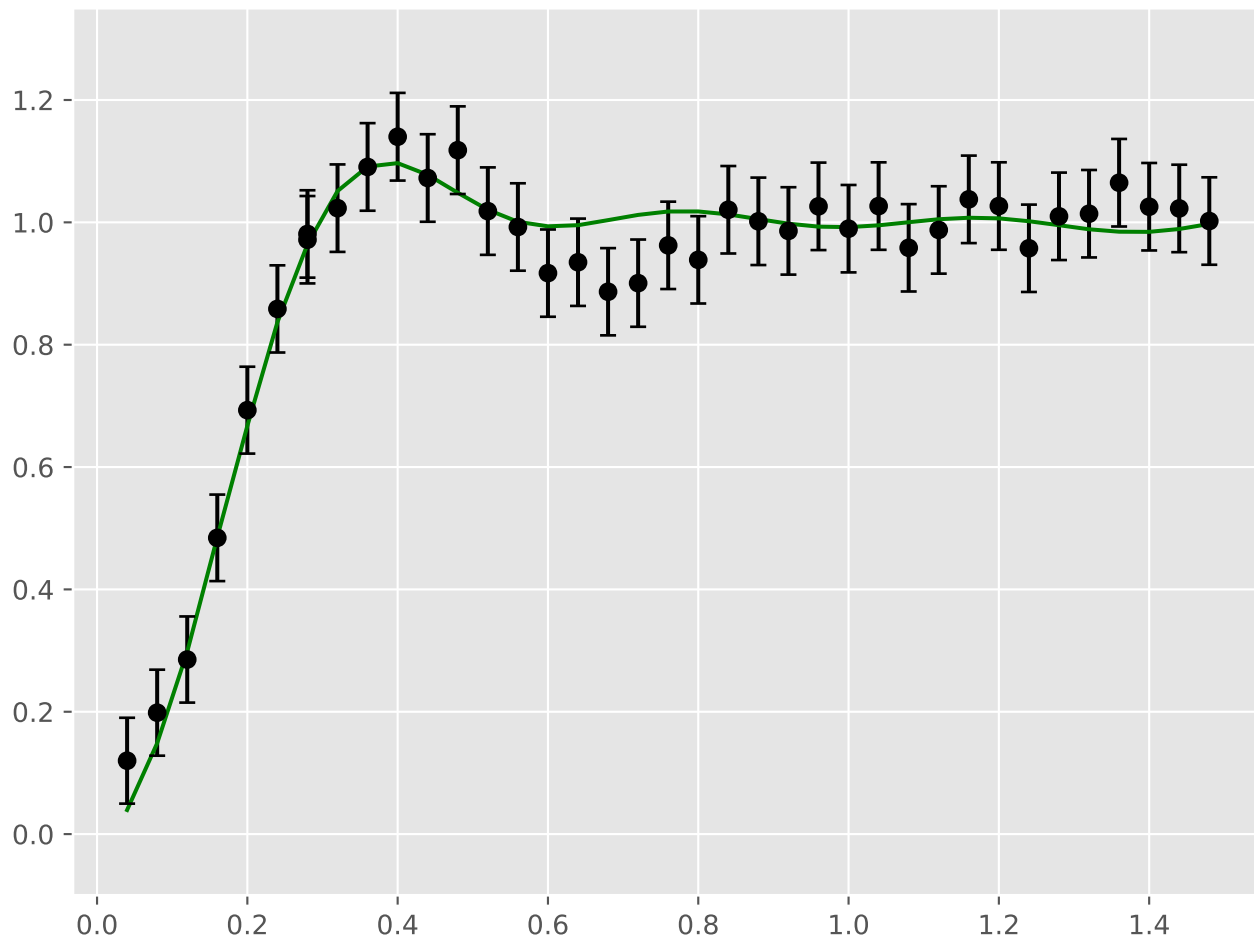

Supplement: Supplementary file 4 — Source data [file 41467_2022_29423_MOESM4_ESM.gz › source_data_2022/Figures_2B-E_SI5_SI6_SI7/Methyl dipolar-coupling measurements. Exptl data, simulations and analysis scripts/fit-figures/322.pdf]

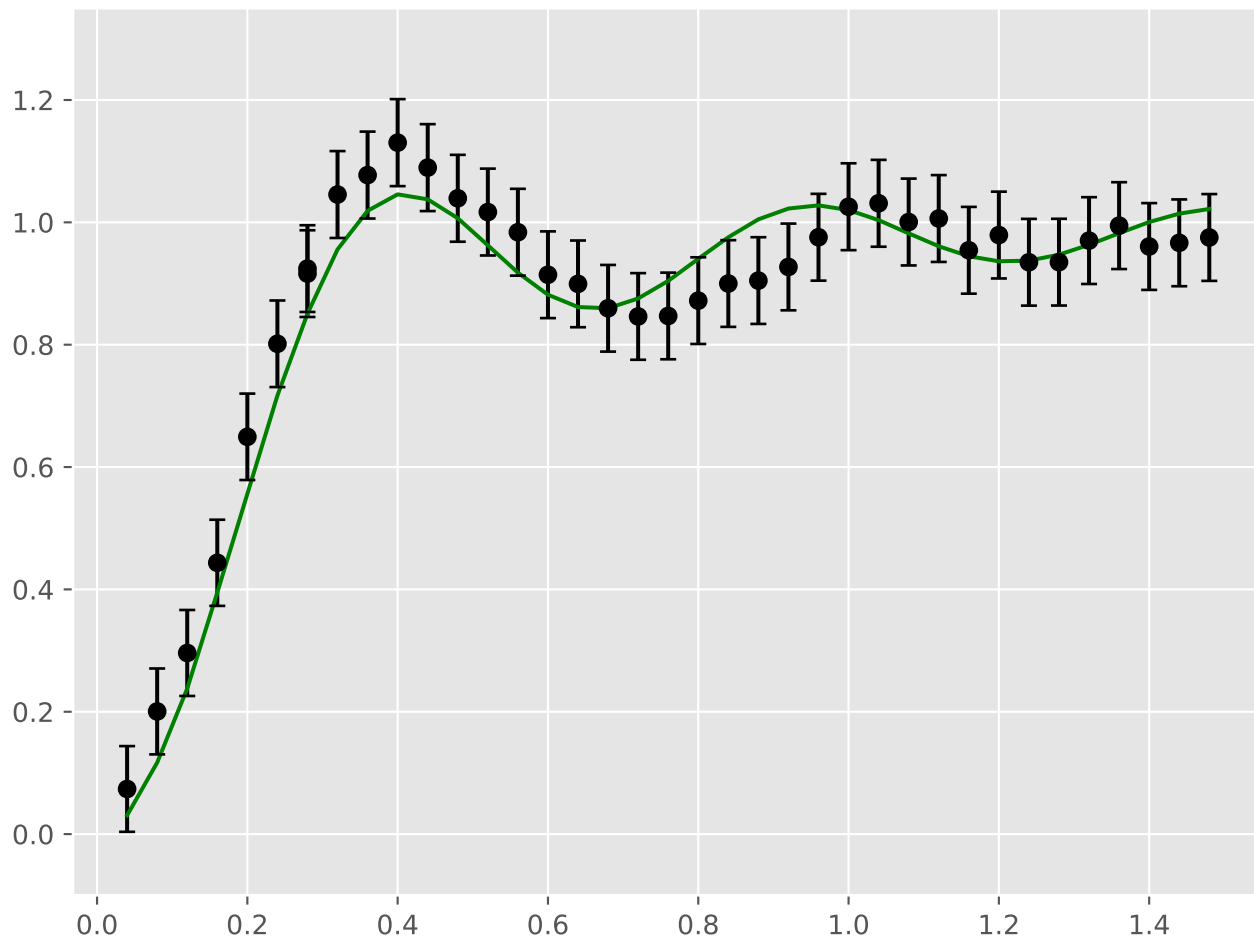

Supplement: Supplementary file 4 — Source data [file 41467_2022_29423_MOESM4_ESM.gz › source_data_2022/Figures_2B-E_SI5_SI6_SI7/Methyl dipolar-coupling measurements. Exptl data, simulations and analysis scripts/fit-figures/334.pdf]

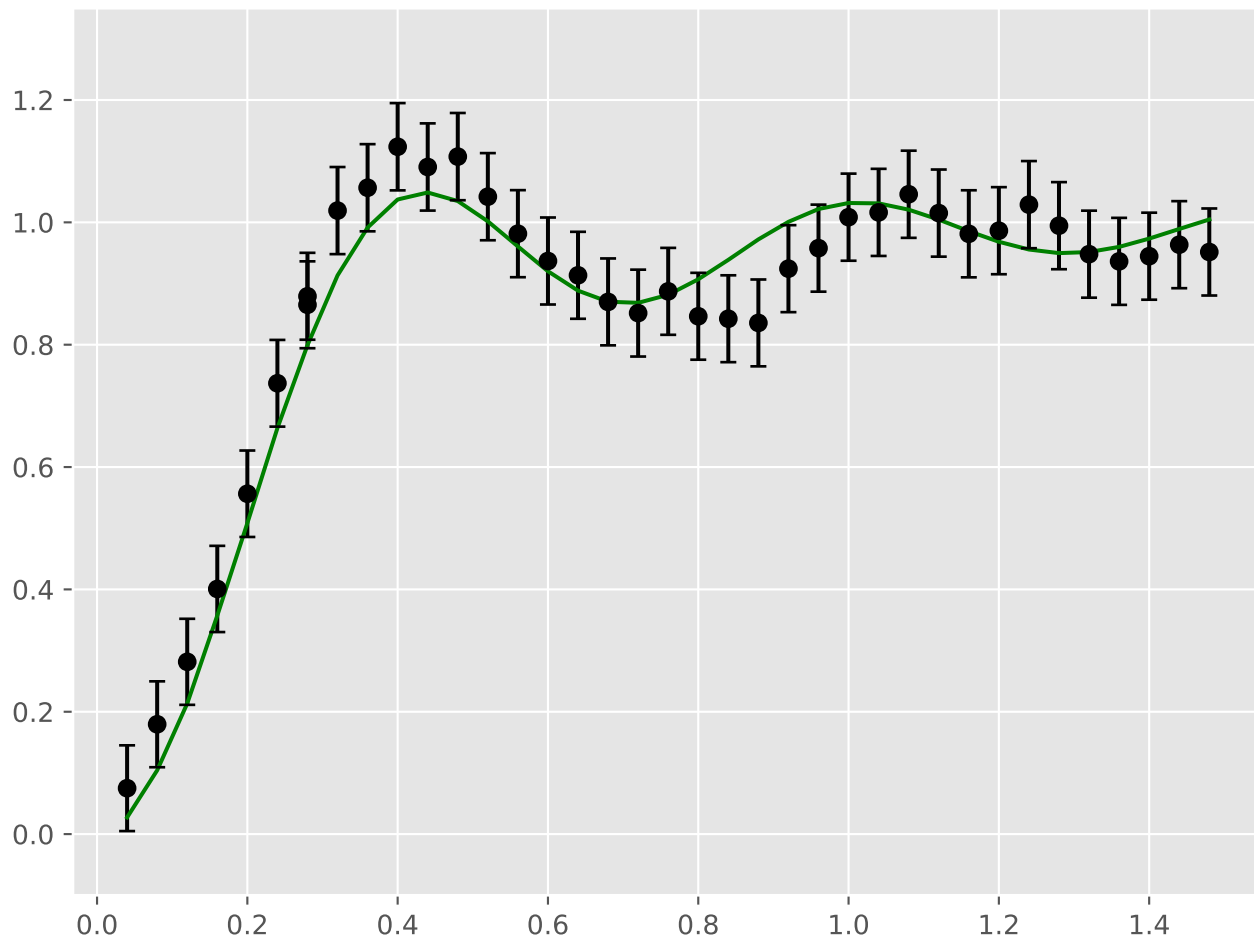

Supplement: Supplementary file 4 — Source data [file 41467_2022_29423_MOESM4_ESM.gz › source_data_2022/Figures_2B-E_SI5_SI6_SI7/Methyl dipolar-coupling measurements. Exptl data, simulations and analysis scripts/fit-figures/329.pdf]

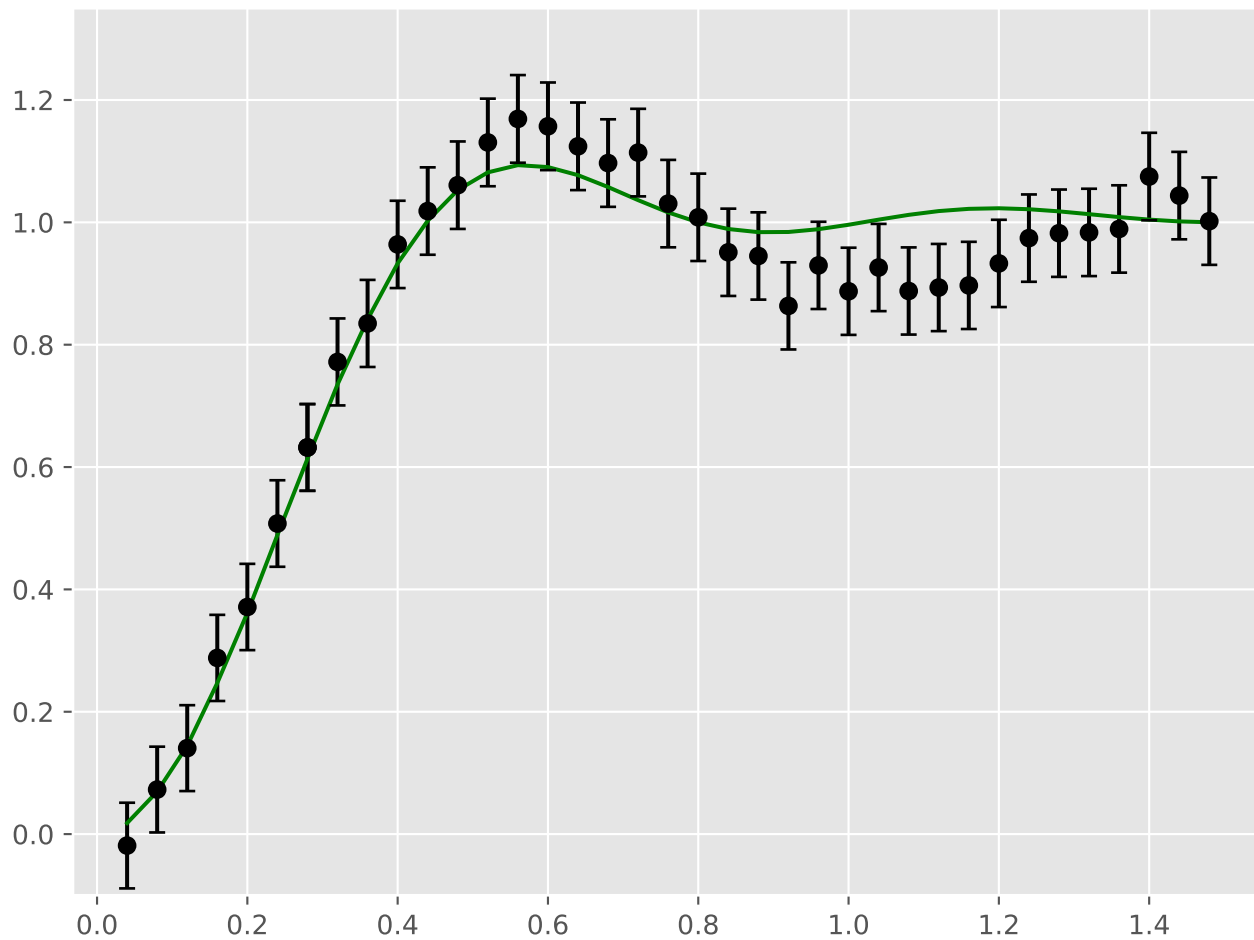

Supplement: Supplementary file 4 — Source data [file 41467_2022_29423_MOESM4_ESM.gz › source_data_2022/Figures_2B-E_SI5_SI6_SI7/Methyl dipolar-coupling measurements. Exptl data, simulations and analysis scripts/fit-figures/351.pdf]

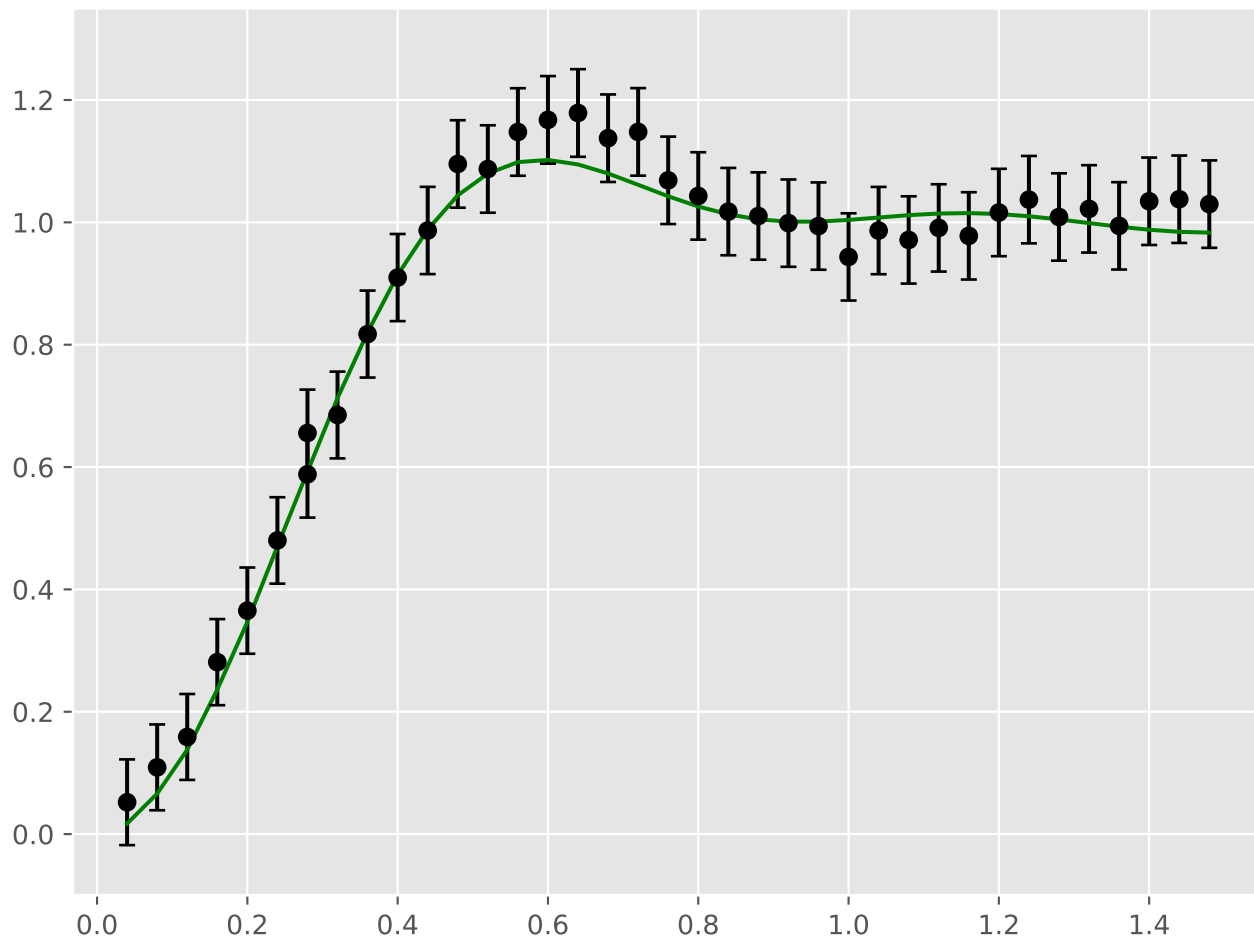

Supplement: Supplementary file 4 — Source data [file 41467_2022_29423_MOESM4_ESM.gz › source_data_2022/Figures_2B-E_SI5_SI6_SI7/Methyl dipolar-coupling measurements. Exptl data, simulations and analysis scripts/fit-figures/348.pdf]

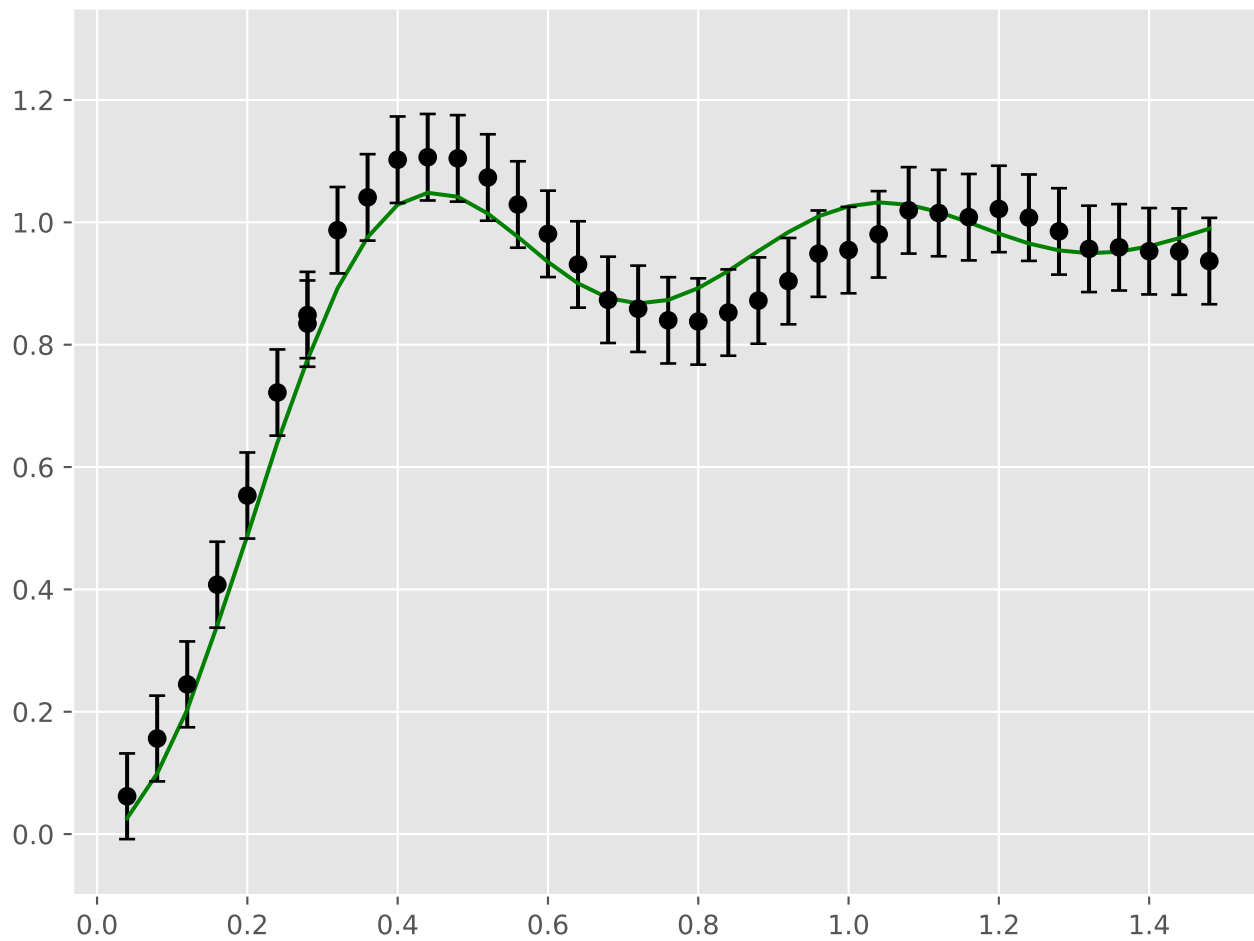

Supplement: Supplementary file 4 — Source data [file 41467_2022_29423_MOESM4_ESM.gz › source_data_2022/Figures_2B-E_SI5_SI6_SI7/Methyl dipolar-coupling measurements. Exptl data, simulations and analysis scripts/fit-figures/353.pdf]

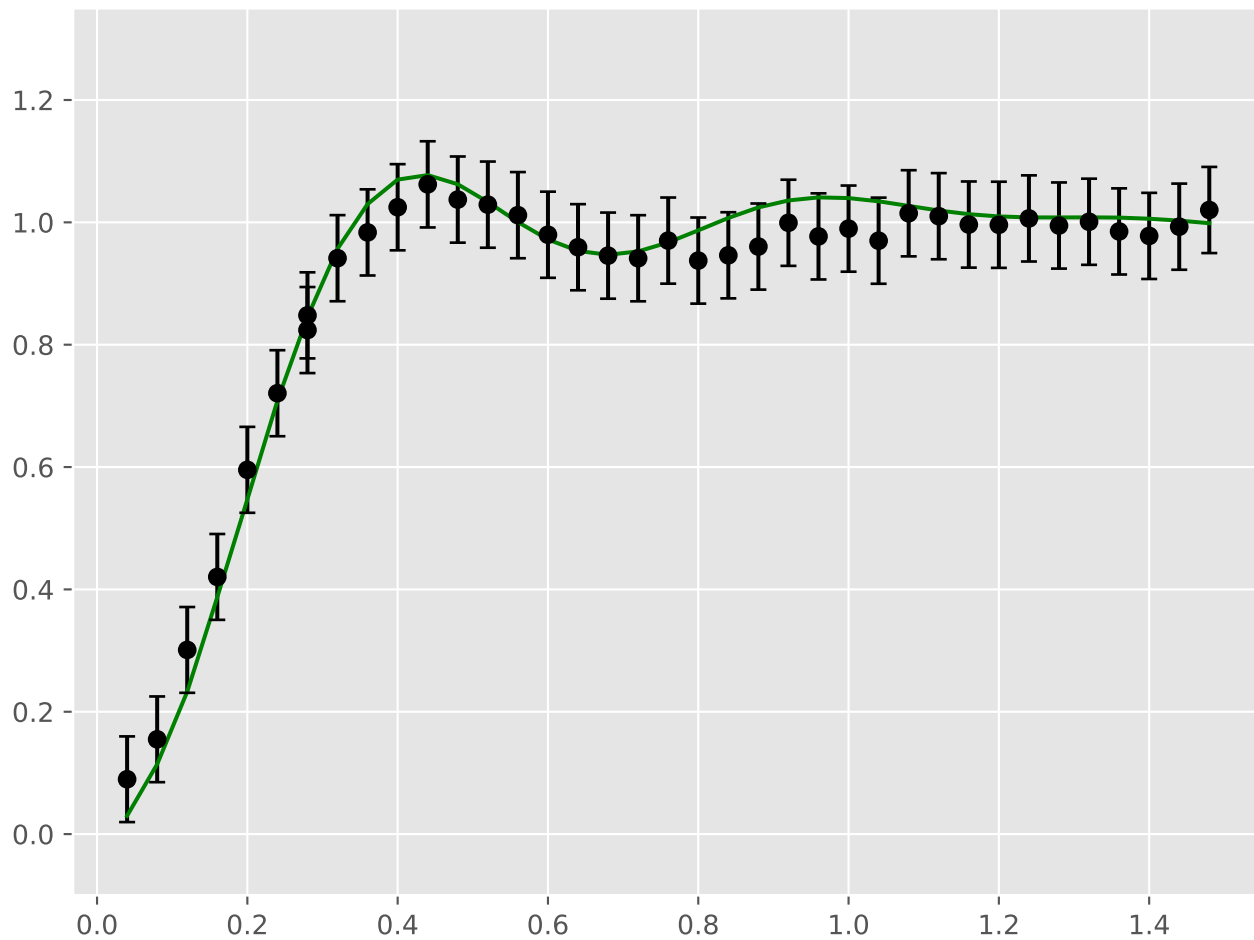

Supplement: Supplementary file 4 — Source data [file 41467_2022_29423_MOESM4_ESM.gz › source_data_2022/Figures_2B-E_SI5_SI6_SI7/Methyl dipolar-coupling measurements. Exptl data, simulations and analysis scripts/fit-figures/52.pdf]

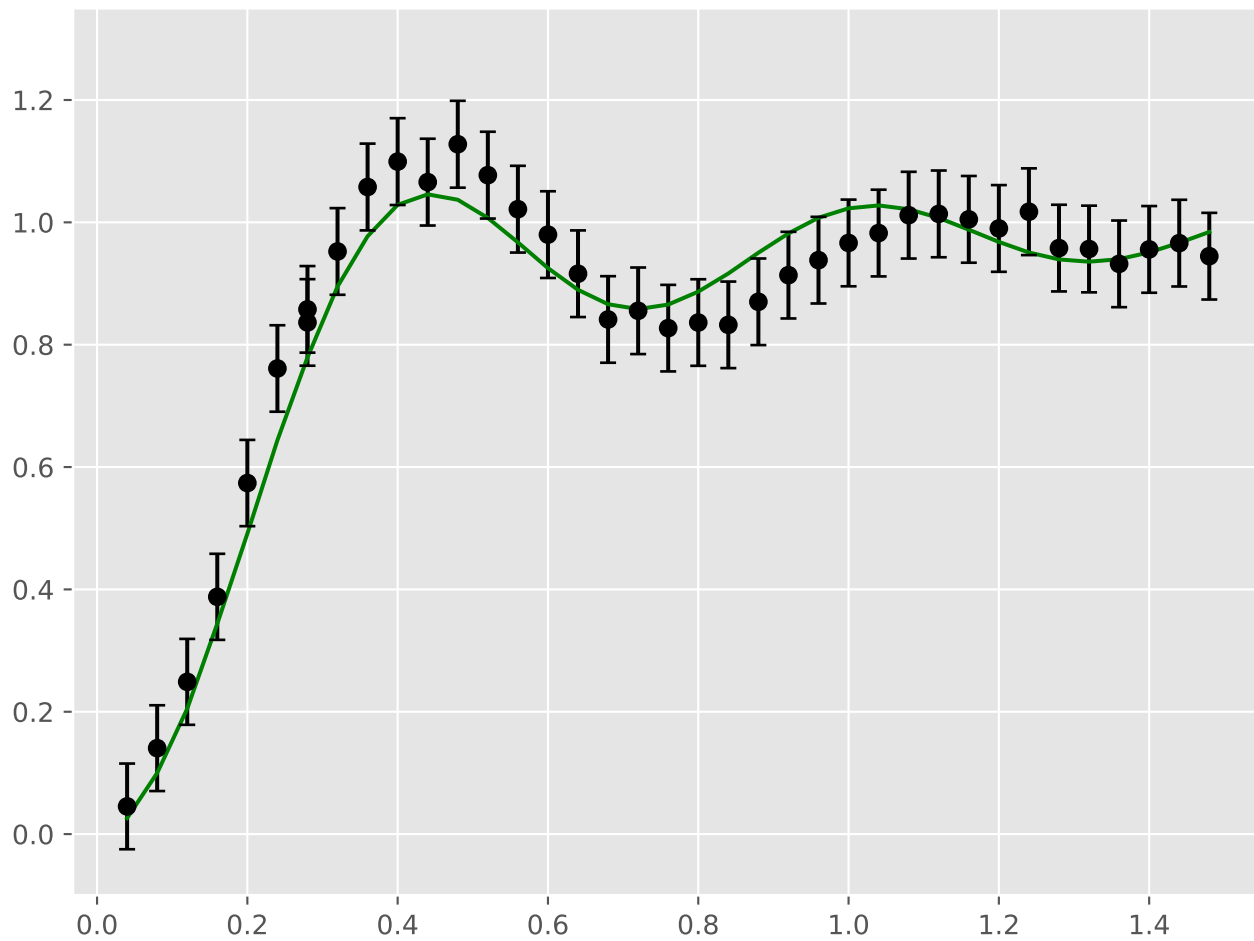

Supplement: Supplementary file 4 — Source data [file 41467_2022_29423_MOESM4_ESM.gz › source_data_2022/Figures_2B-E_SI5_SI6_SI7/Methyl dipolar-coupling measurements. Exptl data, simulations and analysis scripts/fit-figures/63.pdf]

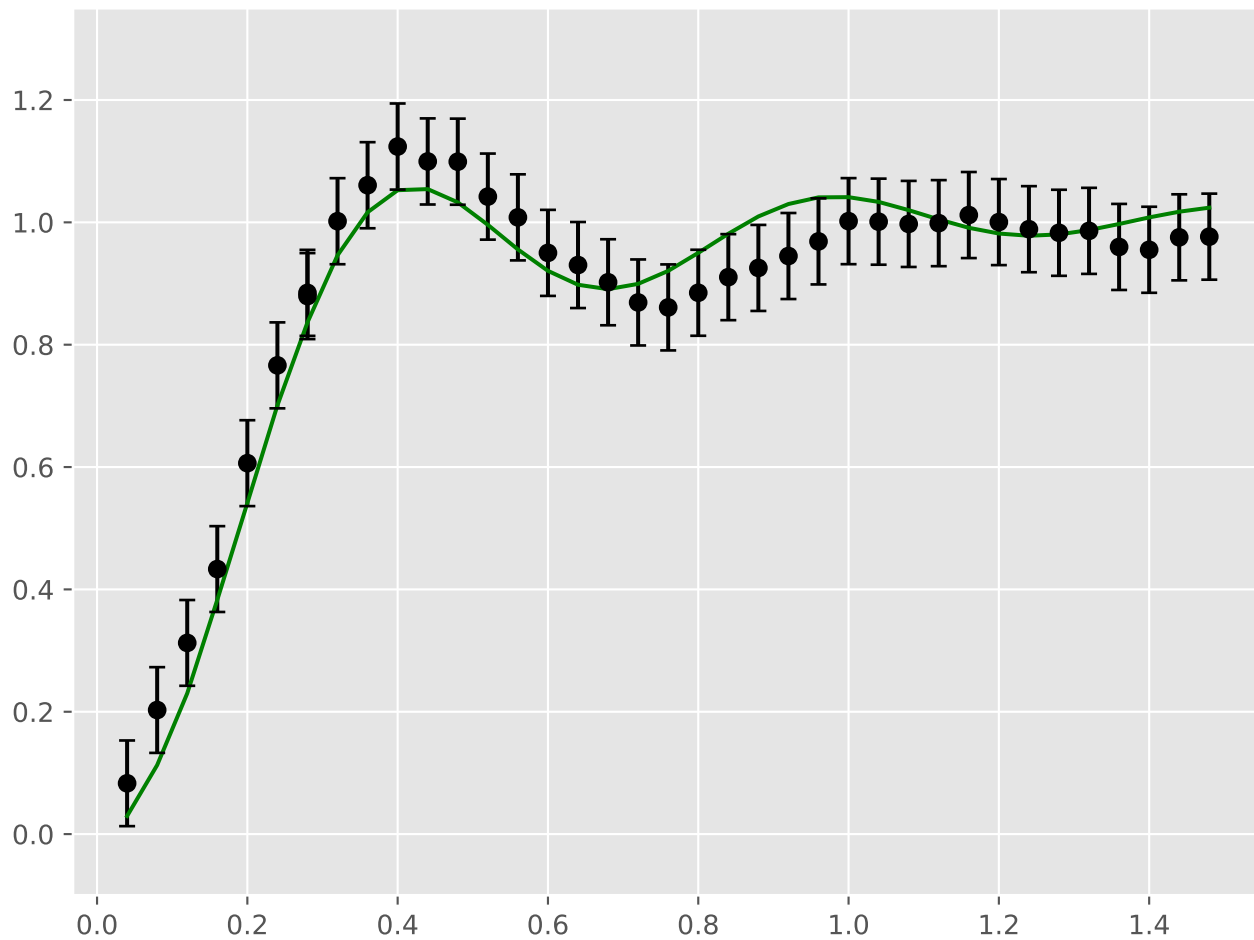

Supplement: Supplementary file 4 — Source data [file 41467_2022_29423_MOESM4_ESM.gz › source_data_2022/Figures_2B-E_SI5_SI6_SI7/Methyl dipolar-coupling measurements. Exptl data, simulations and analysis scripts/fit-figures/85.pdf]

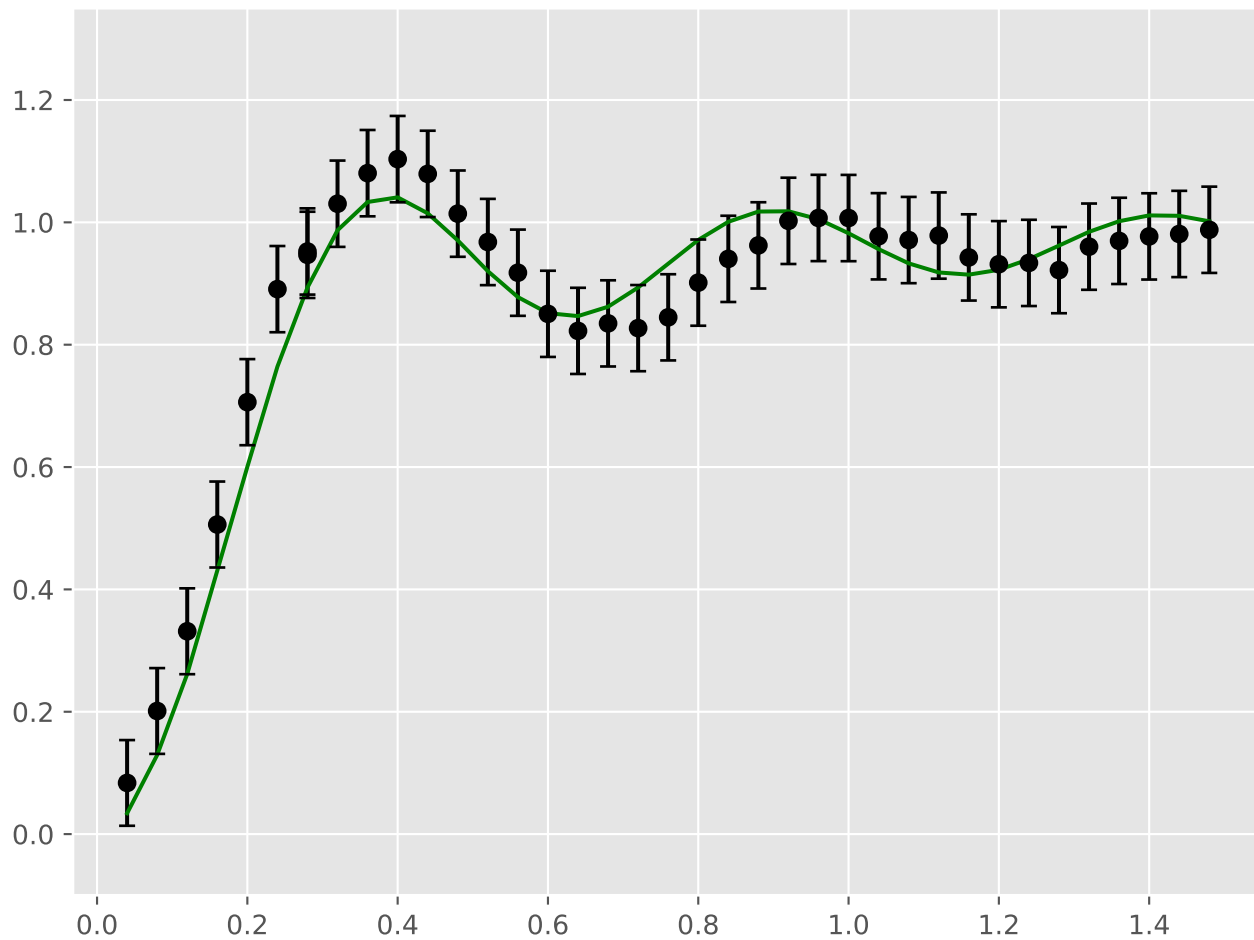

Supplement: Supplementary file 4 — Source data [file 41467_2022_29423_MOESM4_ESM.gz › source_data_2022/Figures_2B-E_SI5_SI6_SI7/Methyl dipolar-coupling measurements. Exptl data, simulations and analysis scripts/fit-figures/338.pdf]

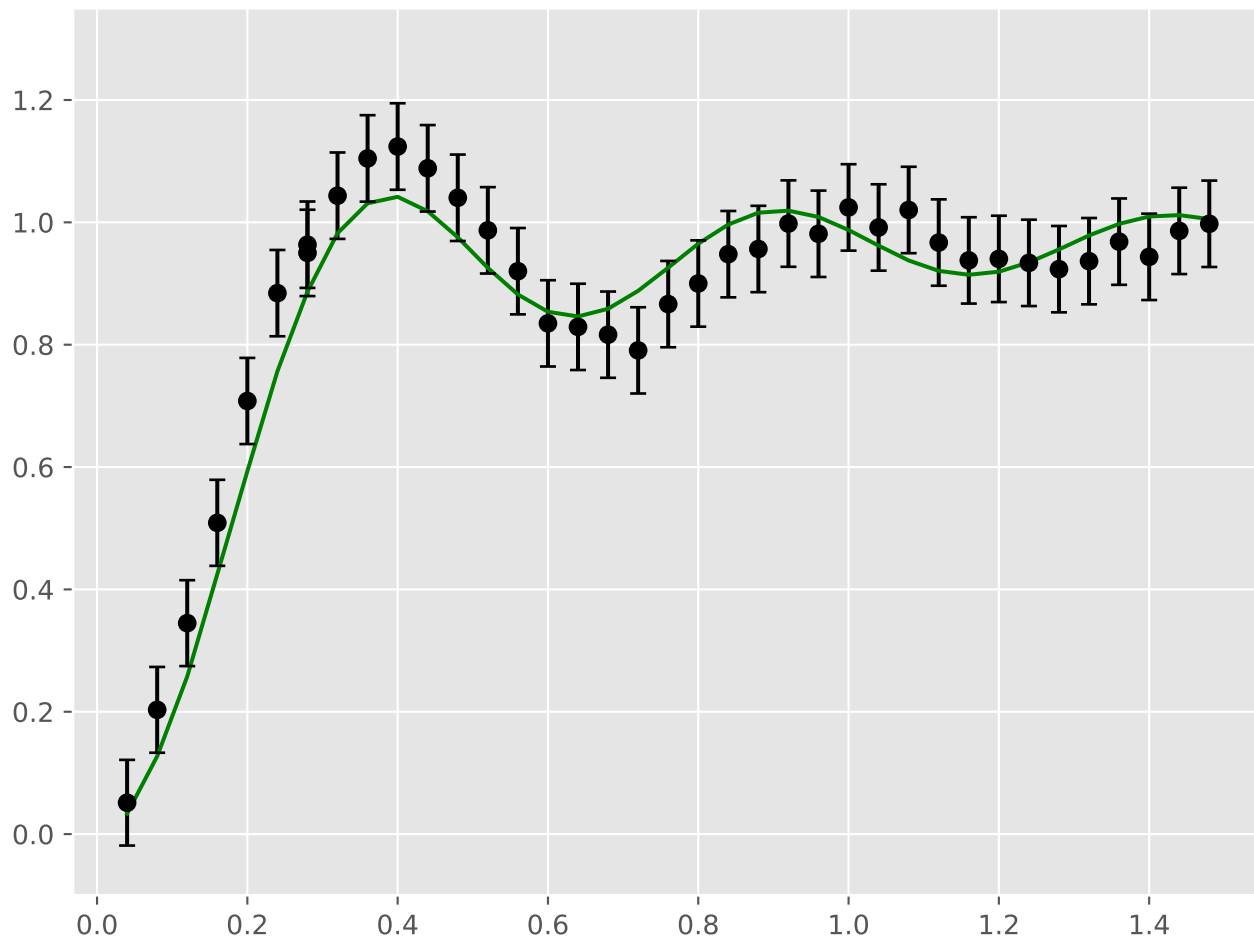

Supplement: Supplementary file 4 — Source data [file 41467_2022_29423_MOESM4_ESM.gz › source_data_2022/Figures_2B-E_SI5_SI6_SI7/Methyl dipolar-coupling measurements. Exptl data, simulations and analysis scripts/fit-figures/105.pdf]

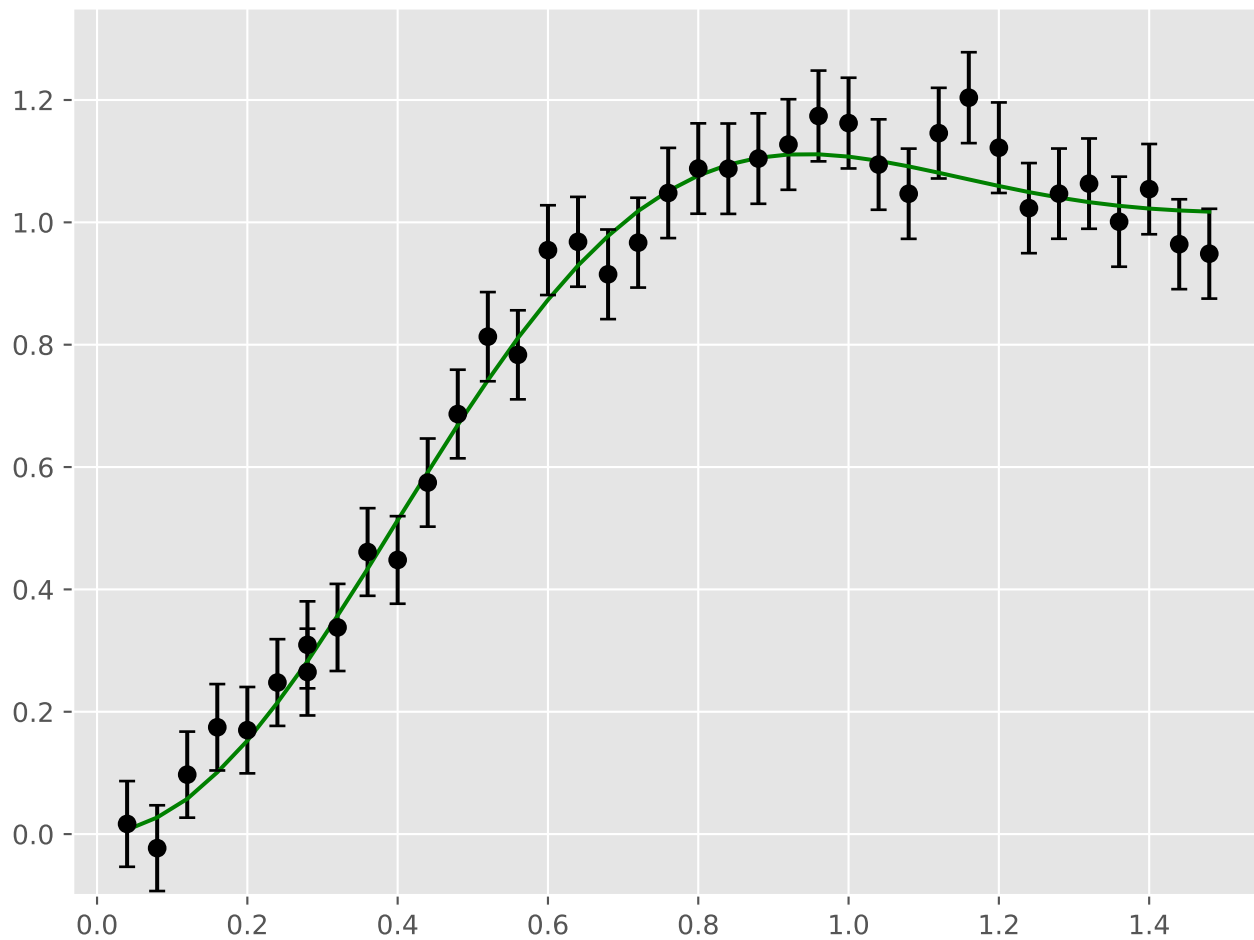

Supplement: Supplementary file 4 — Source data [file 41467_2022_29423_MOESM4_ESM.gz › source_data_2022/Figures_2B-E_SI5_SI6_SI7/Methyl dipolar-coupling measurements. Exptl data, simulations and analysis scripts/fit-figures/113.pdf]

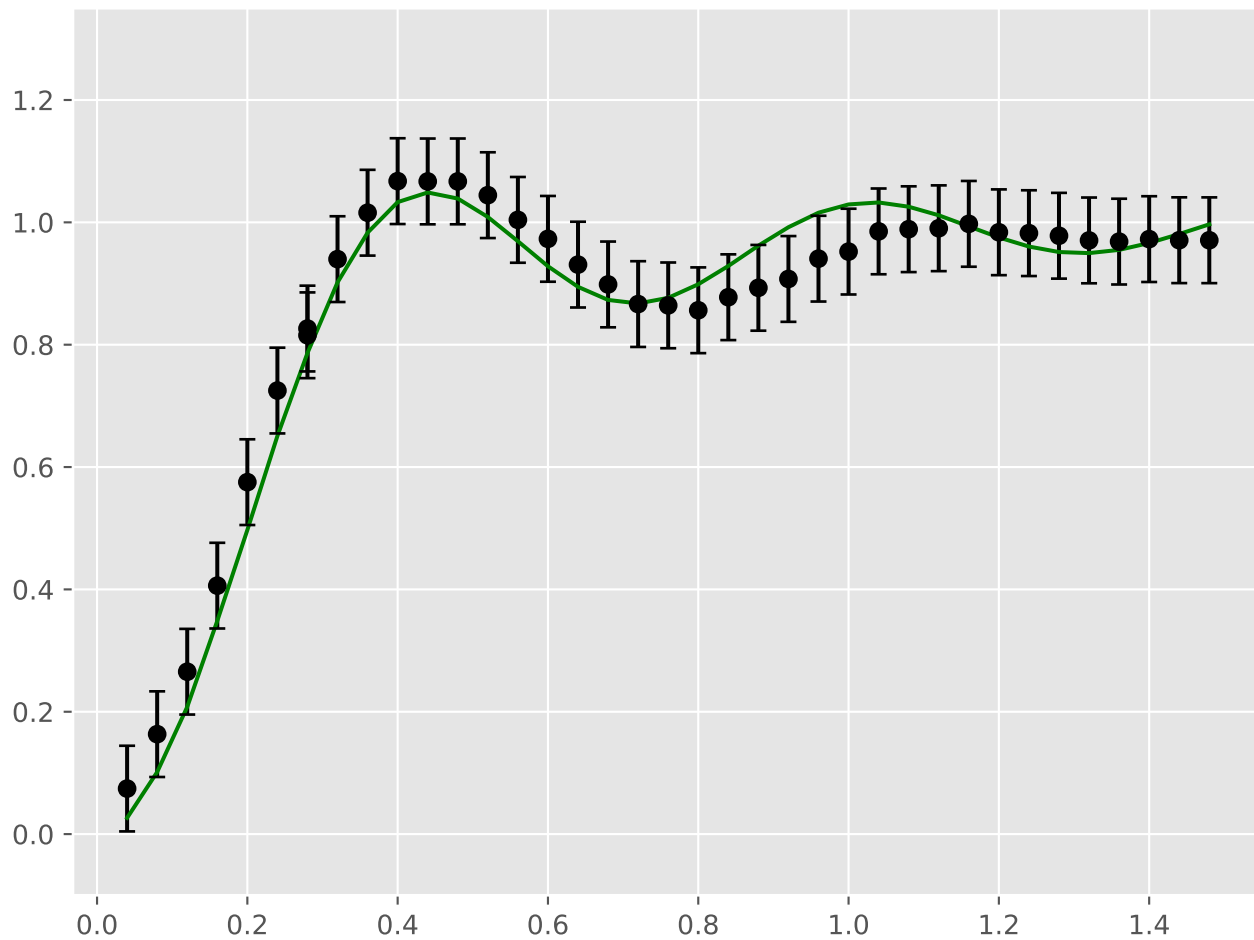

Supplement: Supplementary file 4 — Source data [file 41467_2022_29423_MOESM4_ESM.gz › source_data_2022/Figures_2B-E_SI5_SI6_SI7/Methyl dipolar-coupling measurements. Exptl data, simulations and analysis scripts/fit-figures/107.pdf]

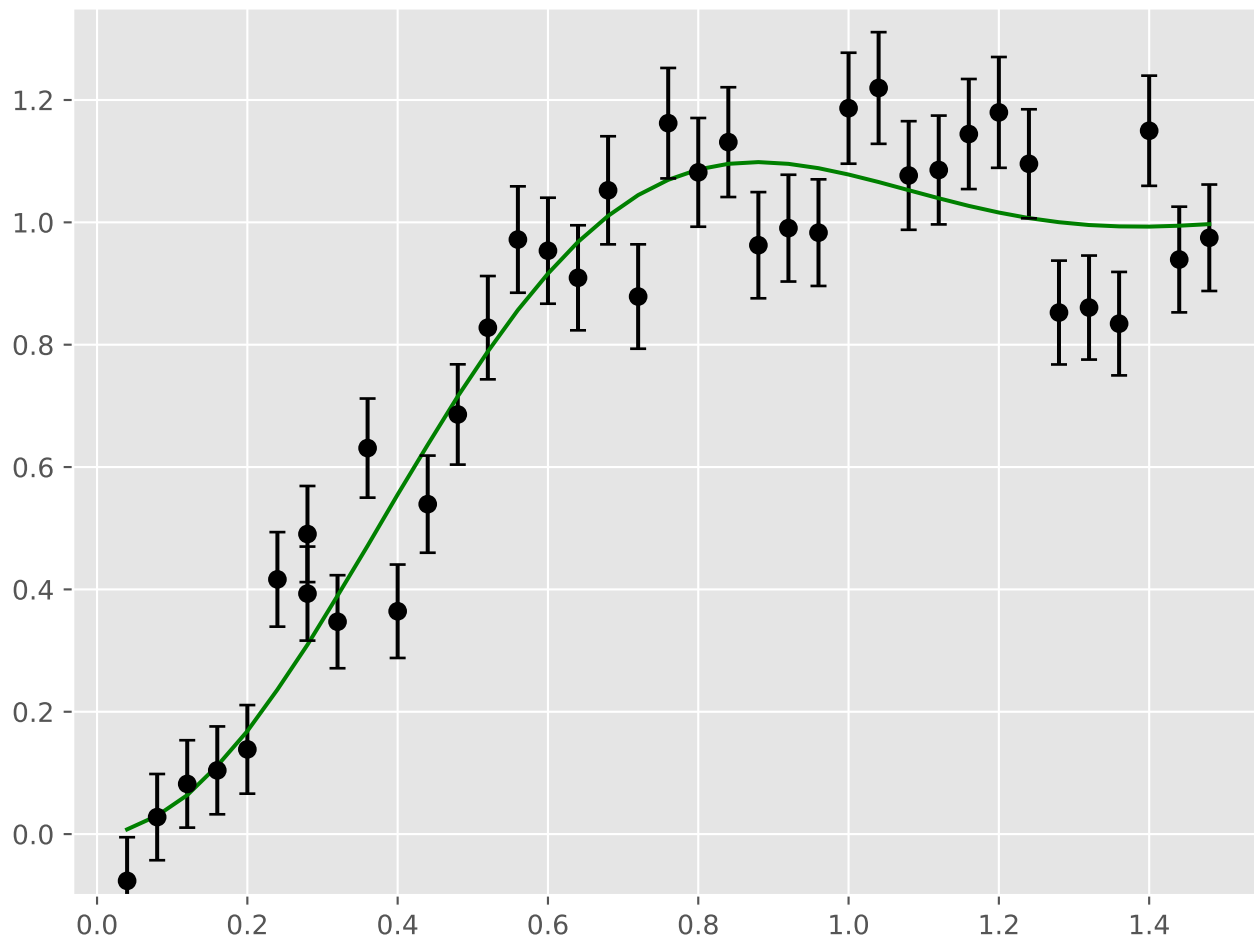

Supplement: Supplementary file 4 — Source data [file 41467_2022_29423_MOESM4_ESM.gz › source_data_2022/Figures_2B-E_SI5_SI6_SI7/Methyl dipolar-coupling measurements. Exptl data, simulations and analysis scripts/fit-figures/139.pdf]

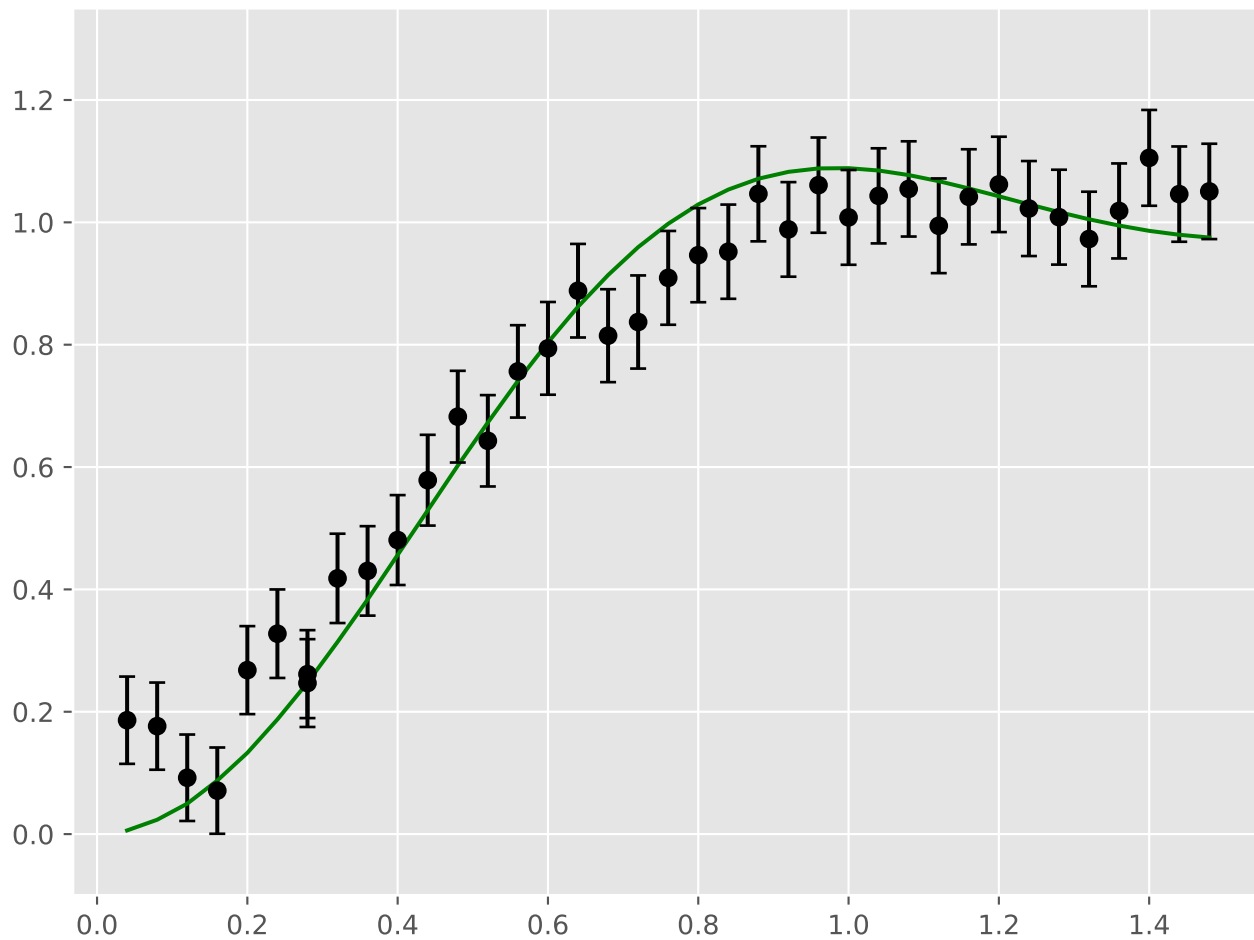

Supplement: Supplementary file 4 — Source data [file 41467_2022_29423_MOESM4_ESM.gz › source_data_2022/Figures_2B-E_SI5_SI6_SI7/Methyl dipolar-coupling measurements. Exptl data, simulations and analysis scripts/fit-figures/120.pdf]

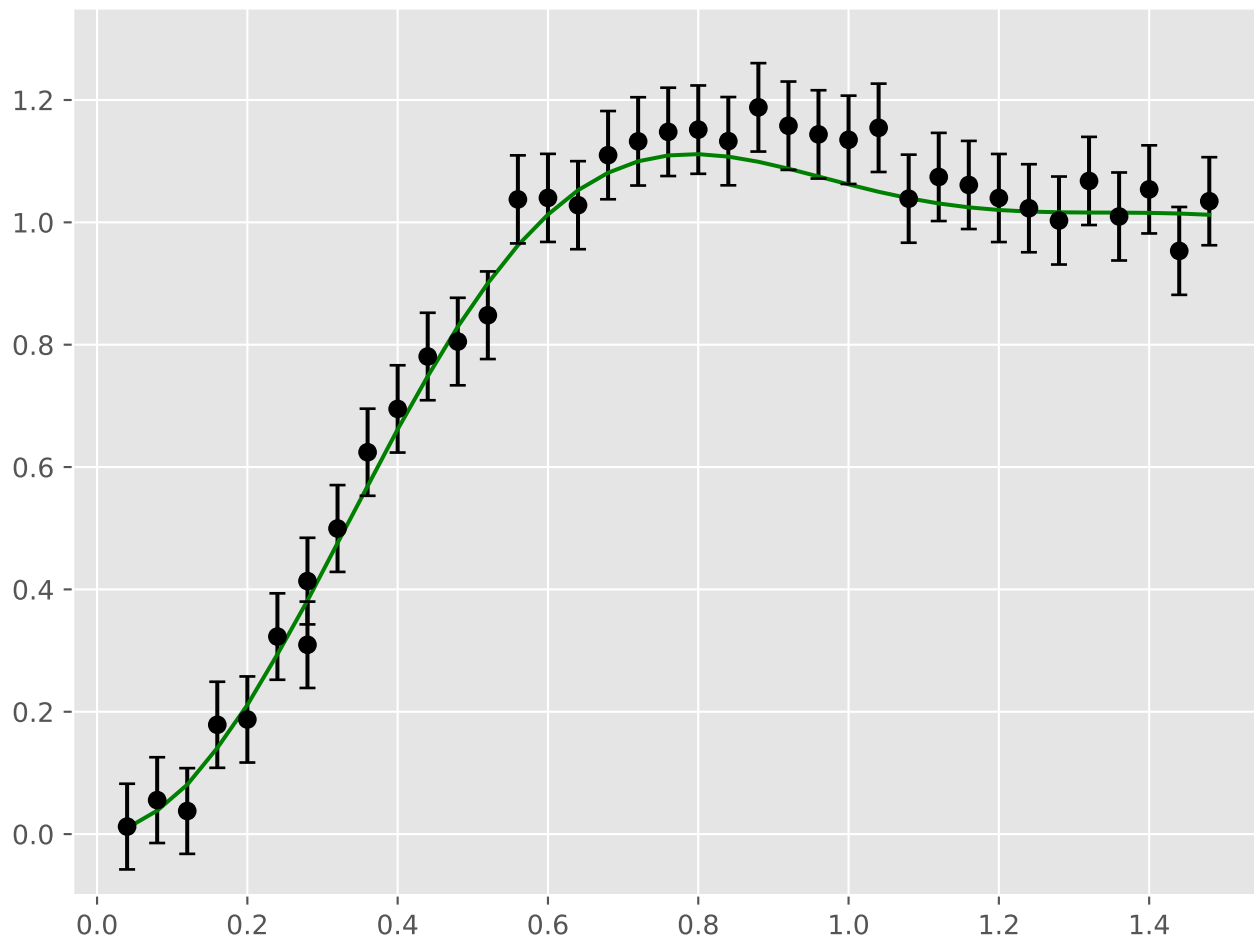

Supplement: Supplementary file 4 — Source data [file 41467_2022_29423_MOESM4_ESM.gz › source_data_2022/Figures_2B-E_SI5_SI6_SI7/Methyl dipolar-coupling measurements. Exptl data, simulations and analysis scripts/fit-figures/141.pdf]

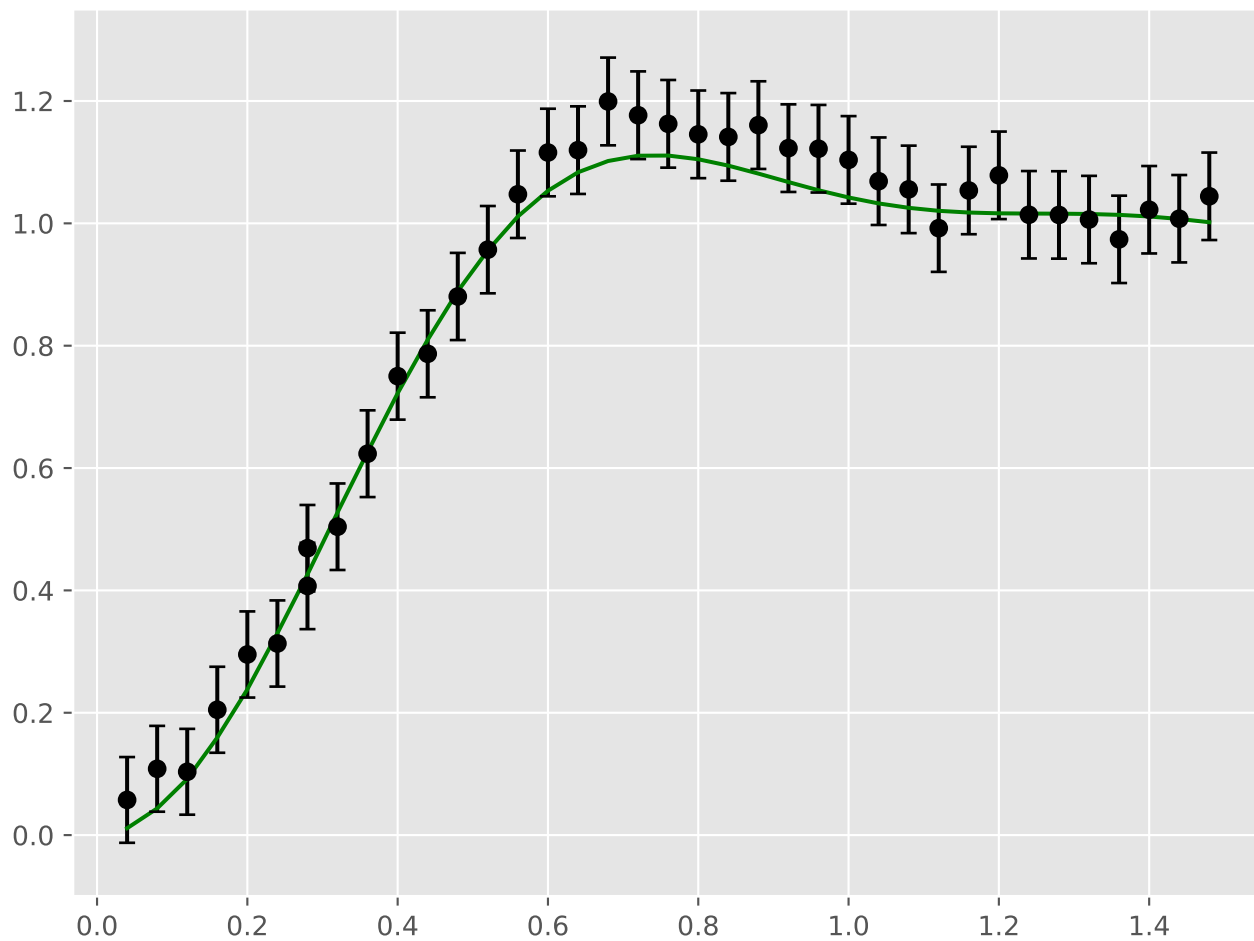

Supplement: Supplementary file 4 — Source data [file 41467_2022_29423_MOESM4_ESM.gz › source_data_2022/Figures_2B-E_SI5_SI6_SI7/Methyl dipolar-coupling measurements. Exptl data, simulations and analysis scripts/fit-figures/158.pdf]

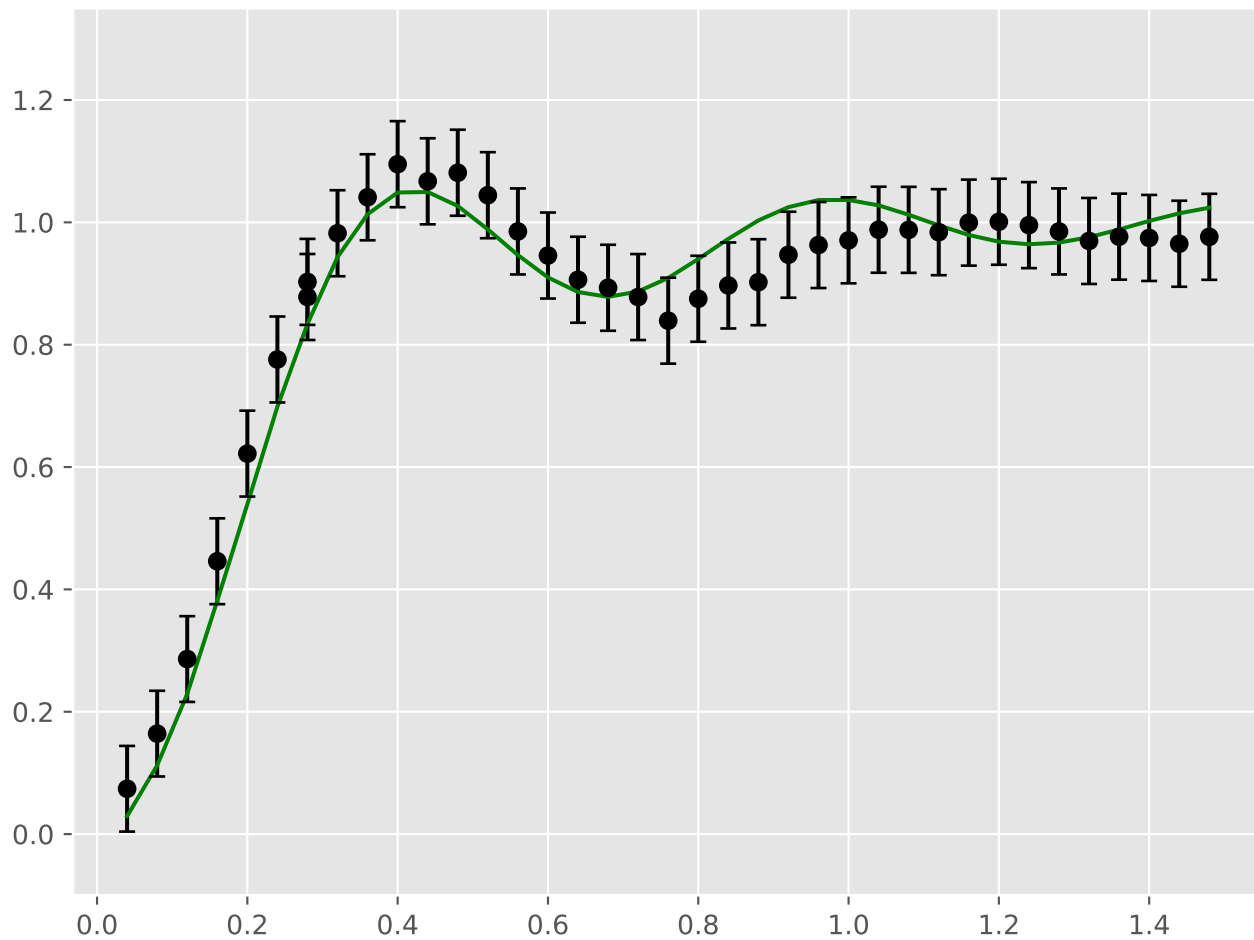

Supplement: Supplementary file 4 — Source data [file 41467_2022_29423_MOESM4_ESM.gz › source_data_2022/Figures_2B-E_SI5_SI6_SI7/Methyl dipolar-coupling measurements. Exptl data, simulations and analysis scripts/fit-figures/143.pdf]

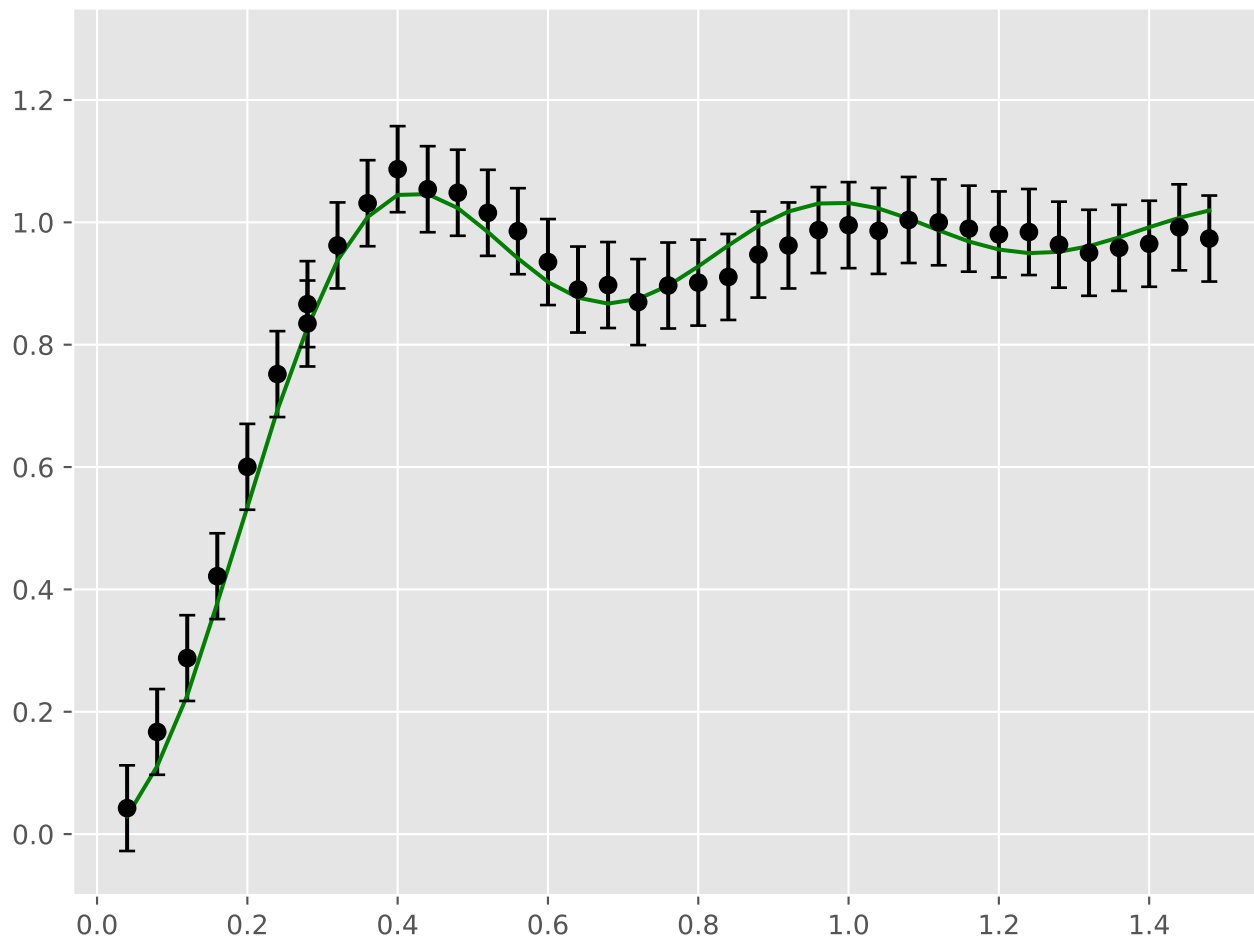

Supplement: Supplementary file 4 — Source data [file 41467_2022_29423_MOESM4_ESM.gz › source_data_2022/Figures_2B-E_SI5_SI6_SI7/Methyl dipolar-coupling measurements. Exptl data, simulations and analysis scripts/fit-figures/15.pdf]

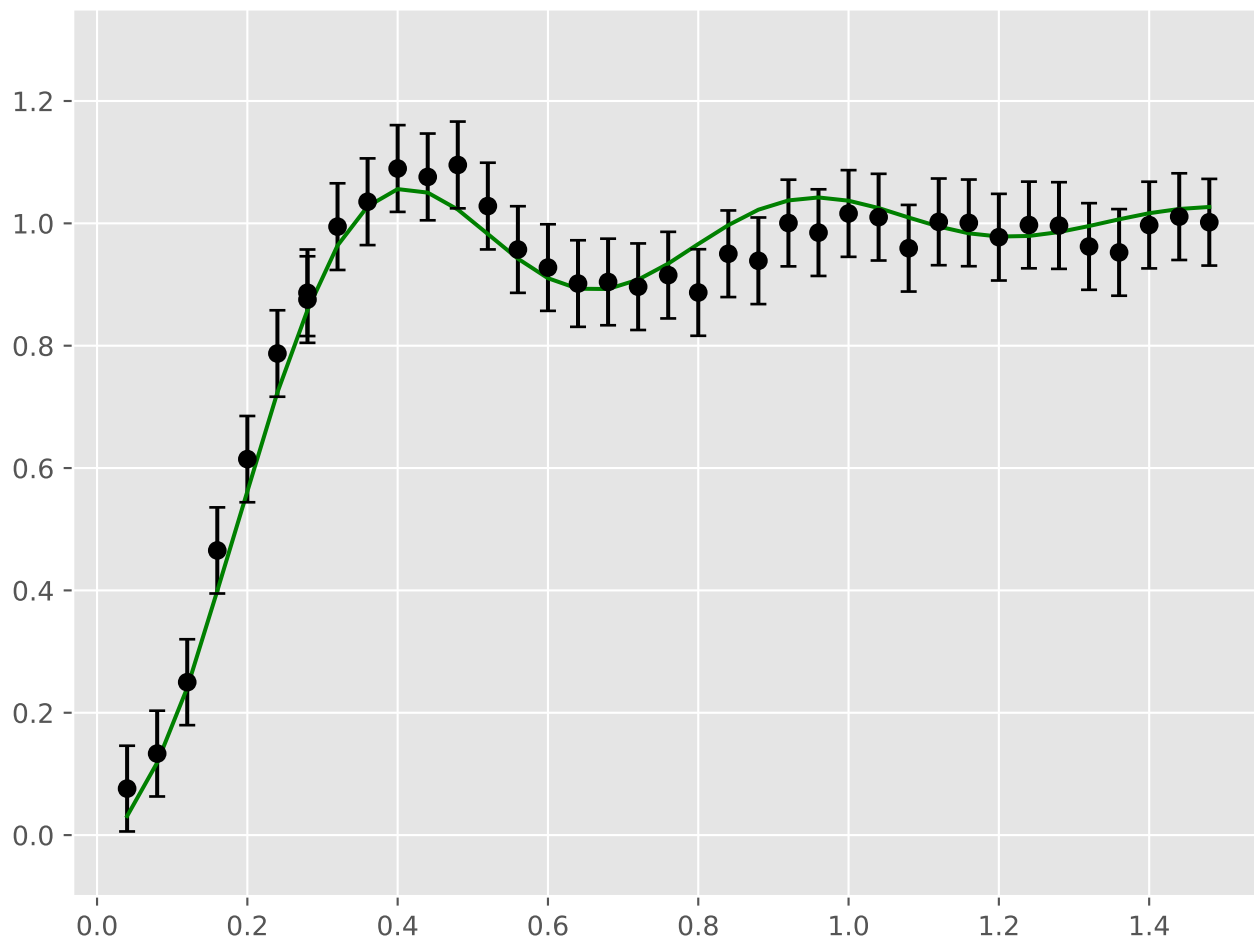

Supplement: Supplementary file 4 — Source data [file 41467_2022_29423_MOESM4_ESM.gz › source_data_2022/Figures_2B-E_SI5_SI6_SI7/Methyl dipolar-coupling measurements. Exptl data, simulations and analysis scripts/fit-figures/16.pdf]

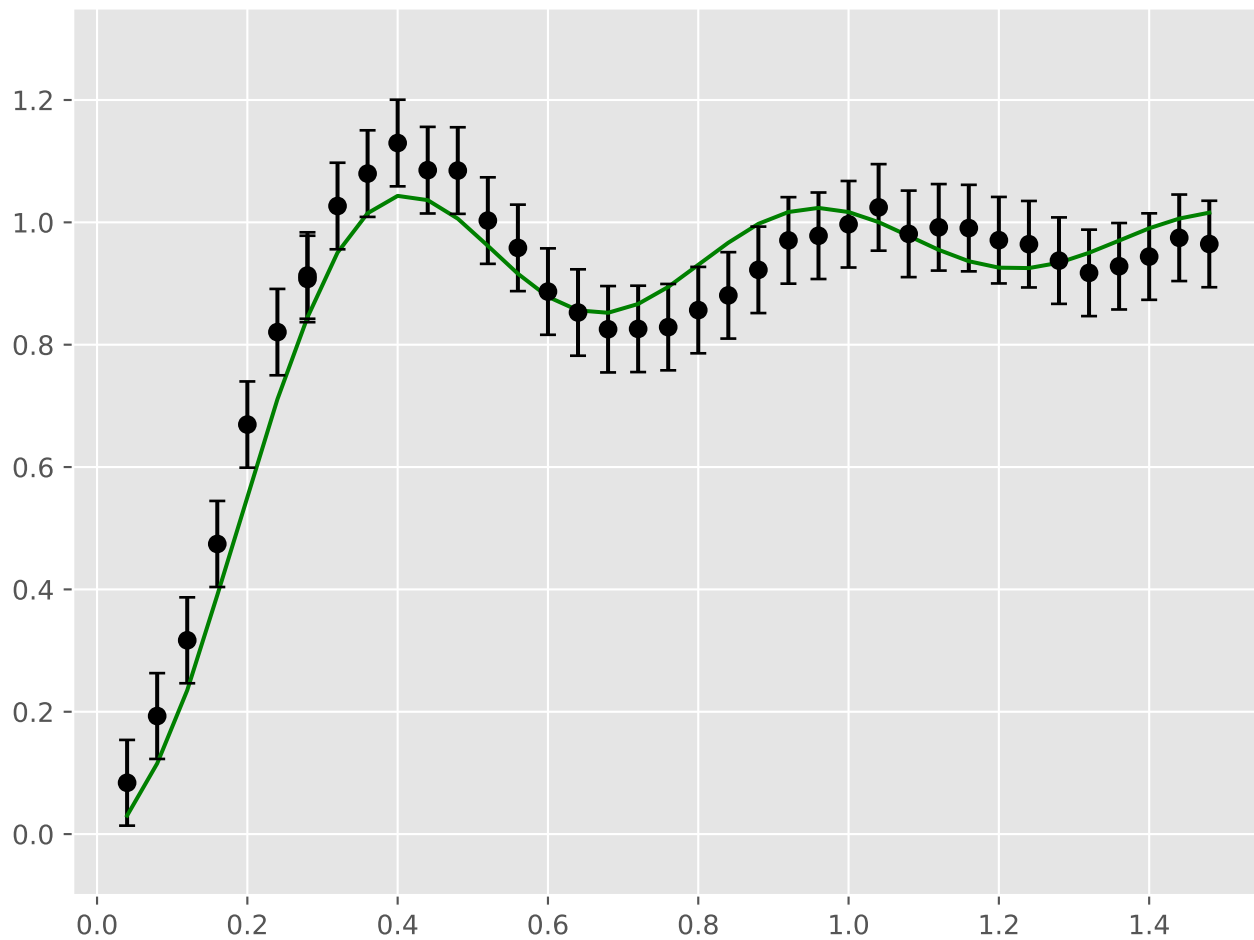

Supplement: Supplementary file 4 — Source data [file 41467_2022_29423_MOESM4_ESM.gz › source_data_2022/Figures_2B-E_SI5_SI6_SI7/Methyl dipolar-coupling measurements. Exptl data, simulations and analysis scripts/fit-figures/161.pdf]

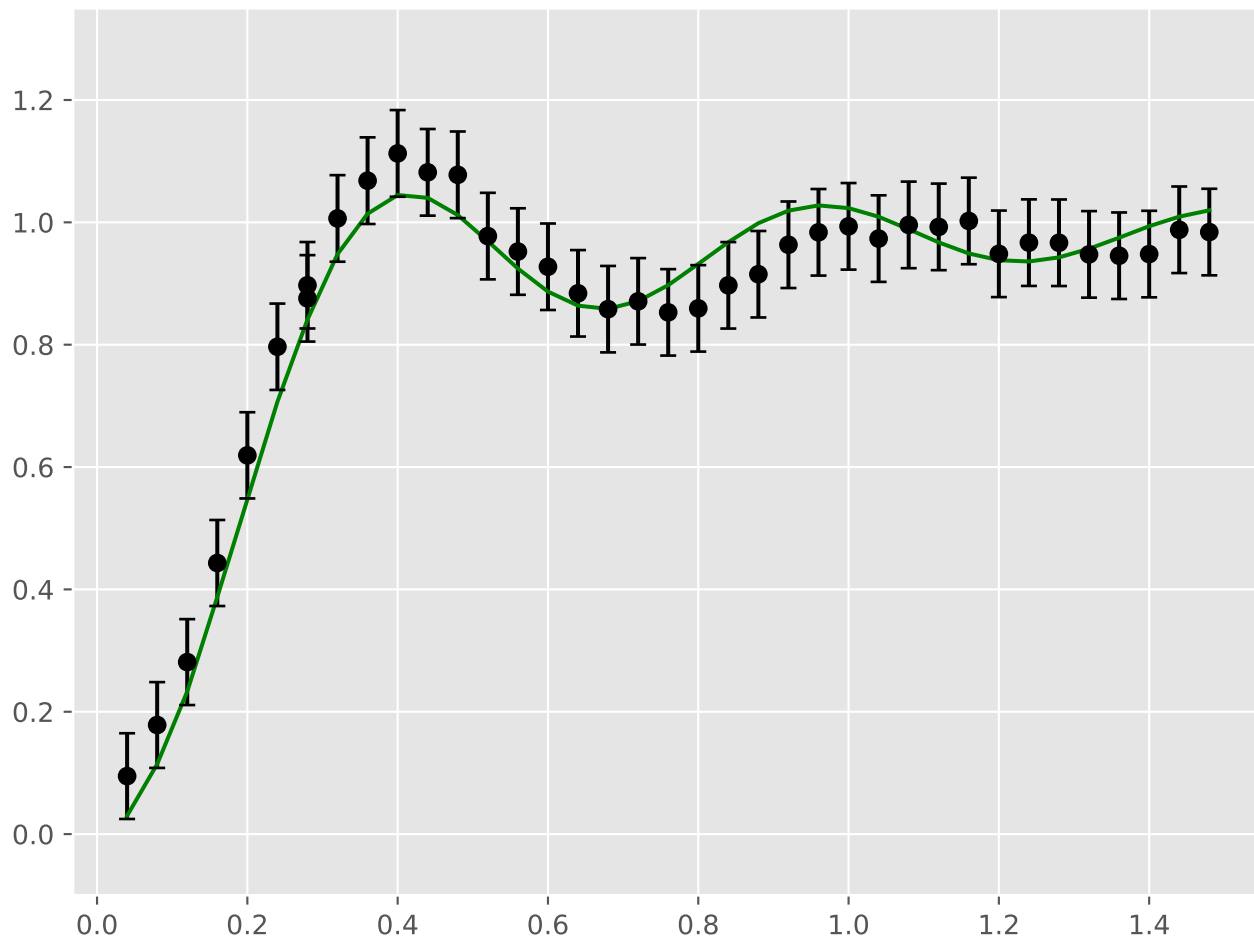

Supplement: Supplementary file 4 — Source data [file 41467_2022_29423_MOESM4_ESM.gz › source_data_2022/Figures_2B-E_SI5_SI6_SI7/Methyl dipolar-coupling measurements. Exptl data, simulations and analysis scripts/fit-figures/12.pdf]

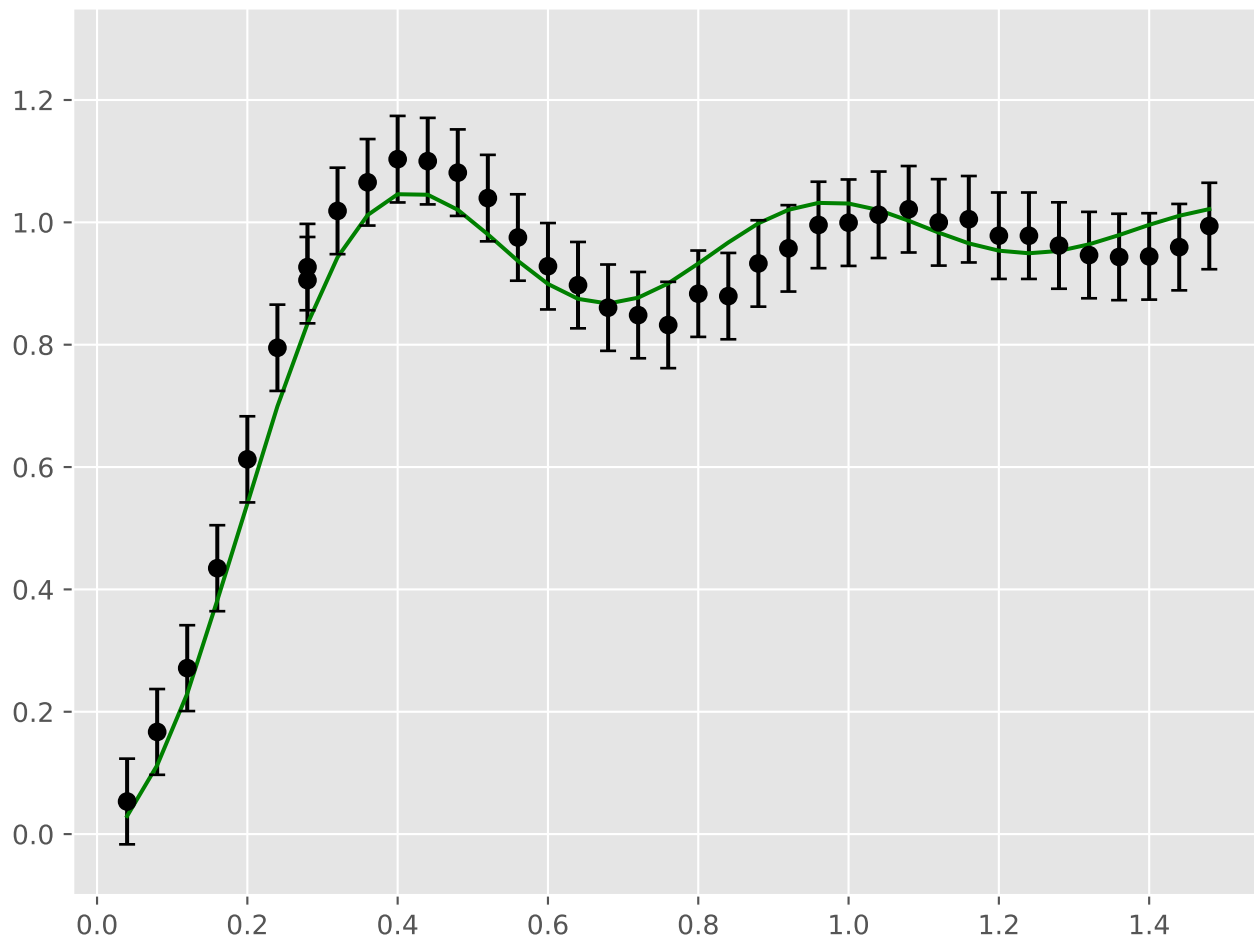

Supplement: Supplementary file 4 — Source data [file 41467_2022_29423_MOESM4_ESM.gz › source_data_2022/Figures_2B-E_SI5_SI6_SI7/Methyl dipolar-coupling measurements. Exptl data, simulations and analysis scripts/fit-figures/162.pdf]

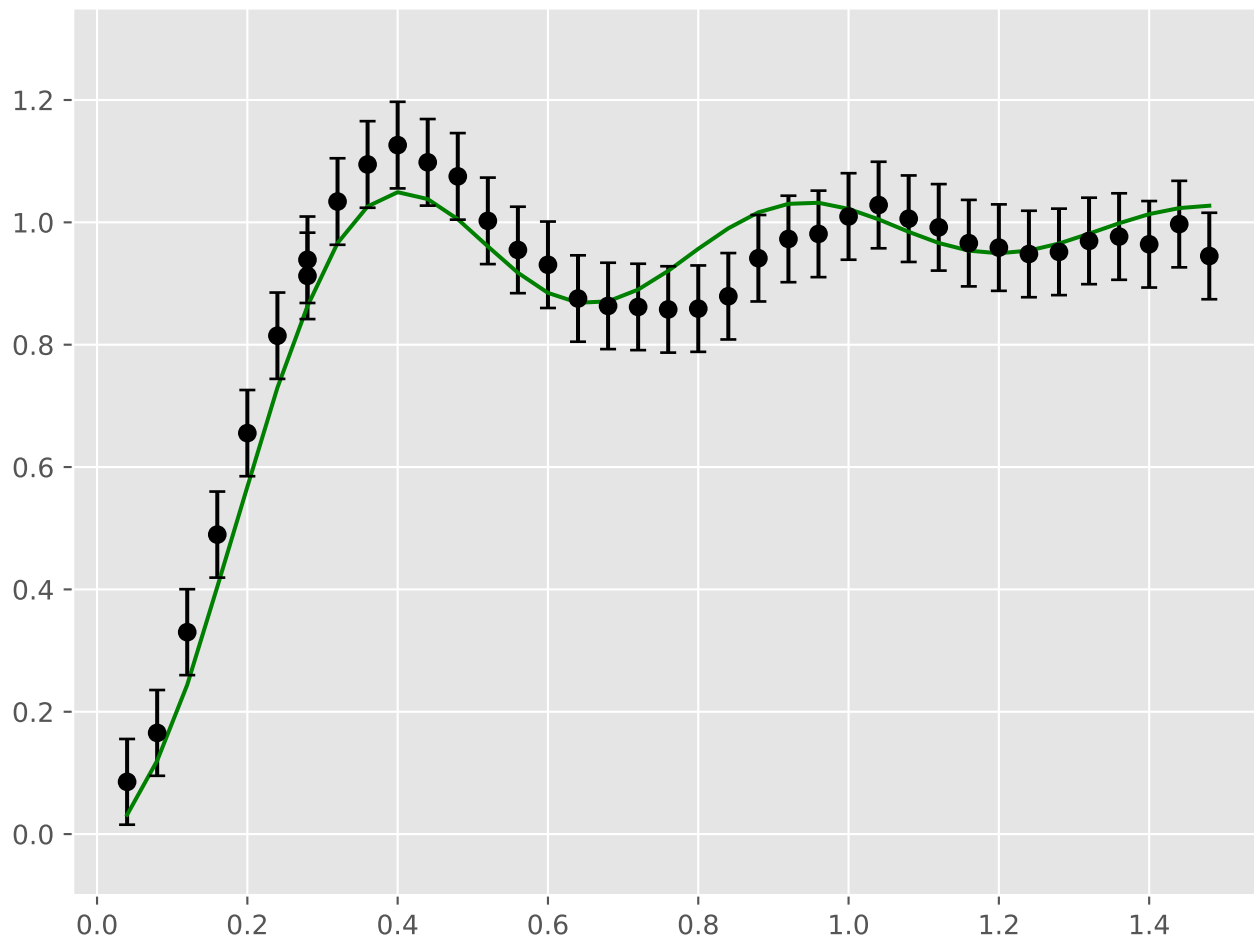

Supplement: Supplementary file 4 — Source data [file 41467_2022_29423_MOESM4_ESM.gz › source_data_2022/Figures_2B-E_SI5_SI6_SI7/Methyl dipolar-coupling measurements. Exptl data, simulations and analysis scripts/fit-figures/185.pdf]

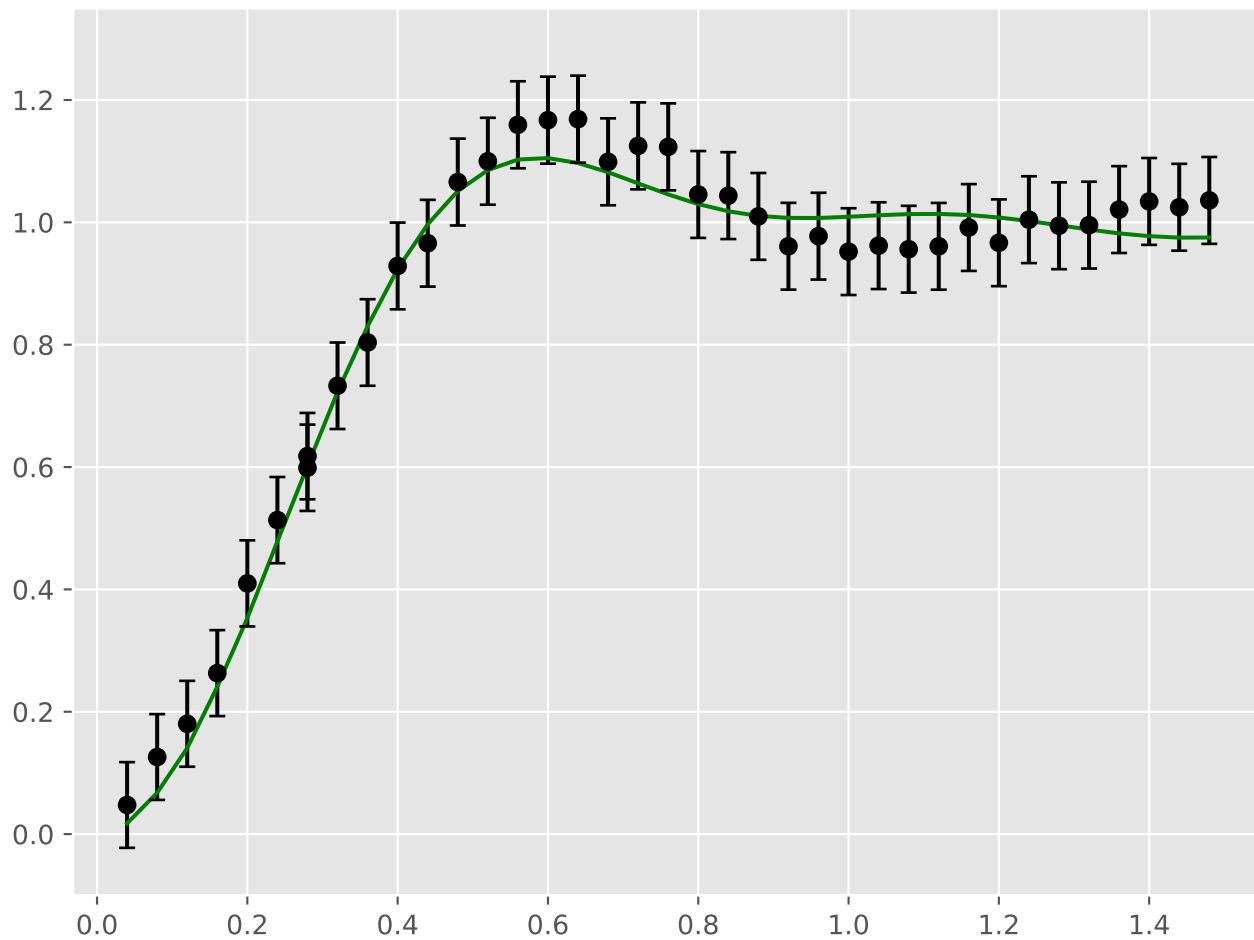

Supplement: Supplementary file 4 — Source data [file 41467_2022_29423_MOESM4_ESM.gz › source_data_2022/Figures_2B-E_SI5_SI6_SI7/Methyl dipolar-coupling measurements. Exptl data, simulations and analysis scripts/fit-figures/171.pdf]

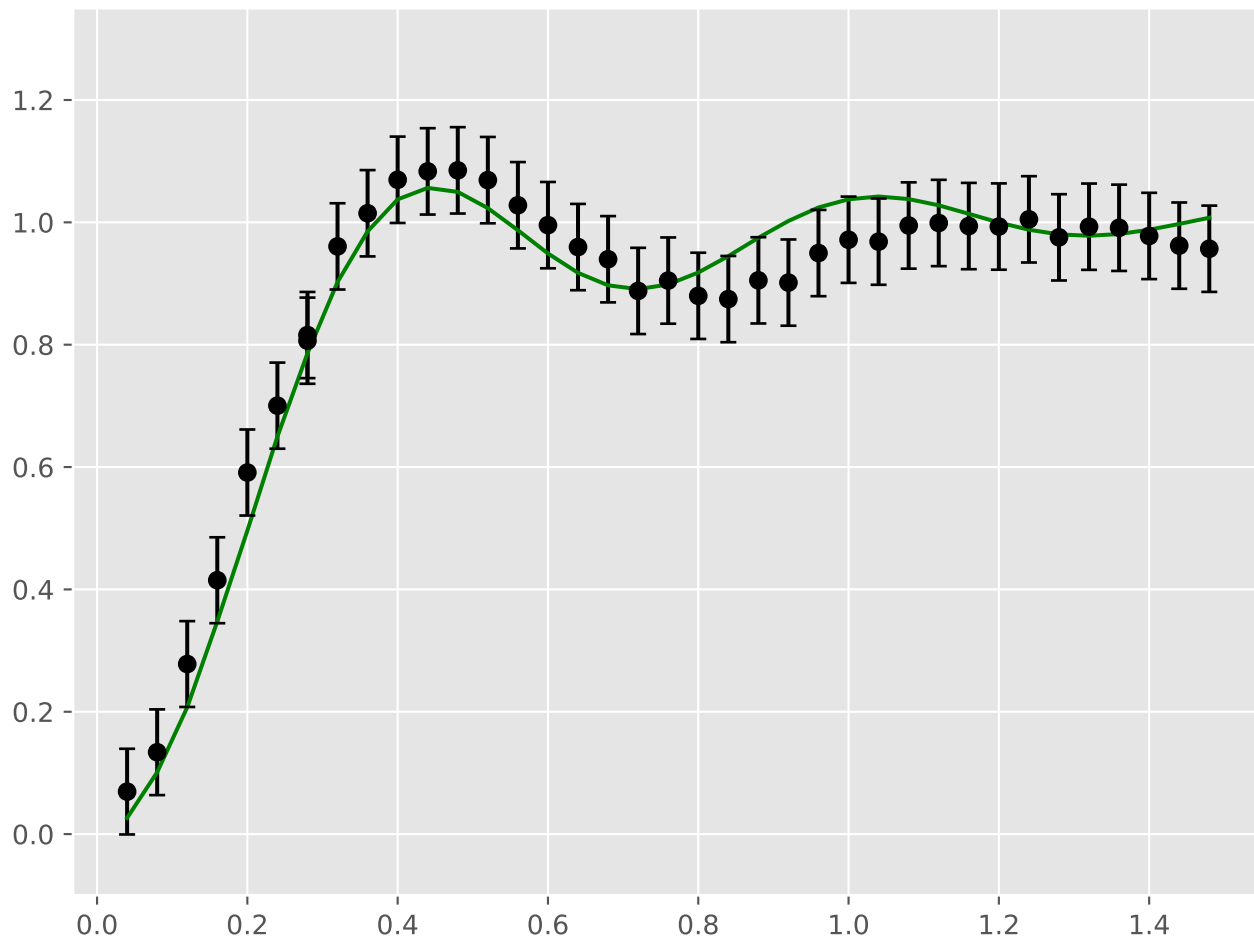

Supplement: Supplementary file 4 — Source data [file 41467_2022_29423_MOESM4_ESM.gz › source_data_2022/Figures_2B-E_SI5_SI6_SI7/Methyl dipolar-coupling measurements. Exptl data, simulations and analysis scripts/fit-figures/177.pdf]

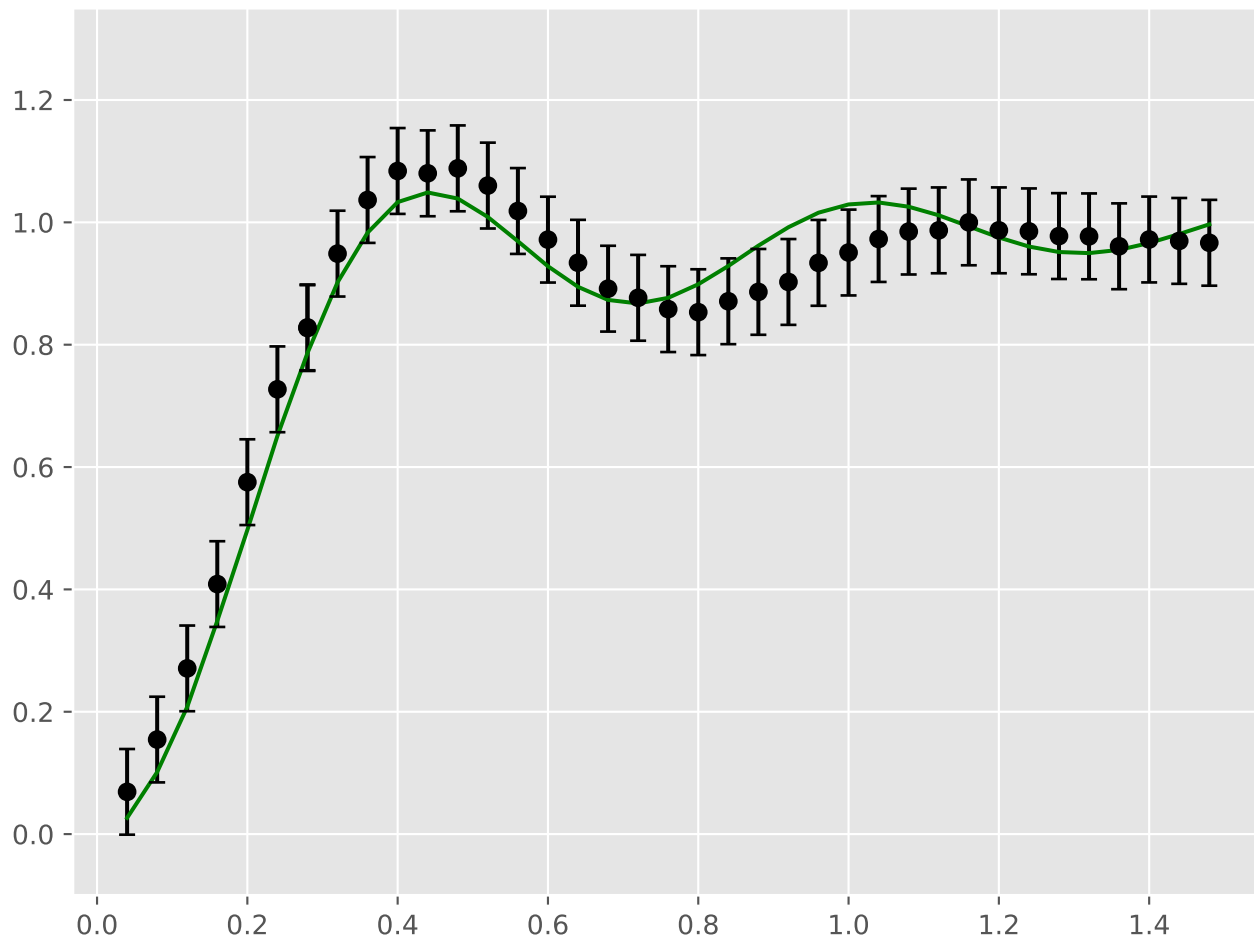

Supplement: Supplementary file 4 — Source data [file 41467_2022_29423_MOESM4_ESM.gz › source_data_2022/Figures_2B-E_SI5_SI6_SI7/Methyl dipolar-coupling measurements. Exptl data, simulations and analysis scripts/fit-figures/190.pdf]

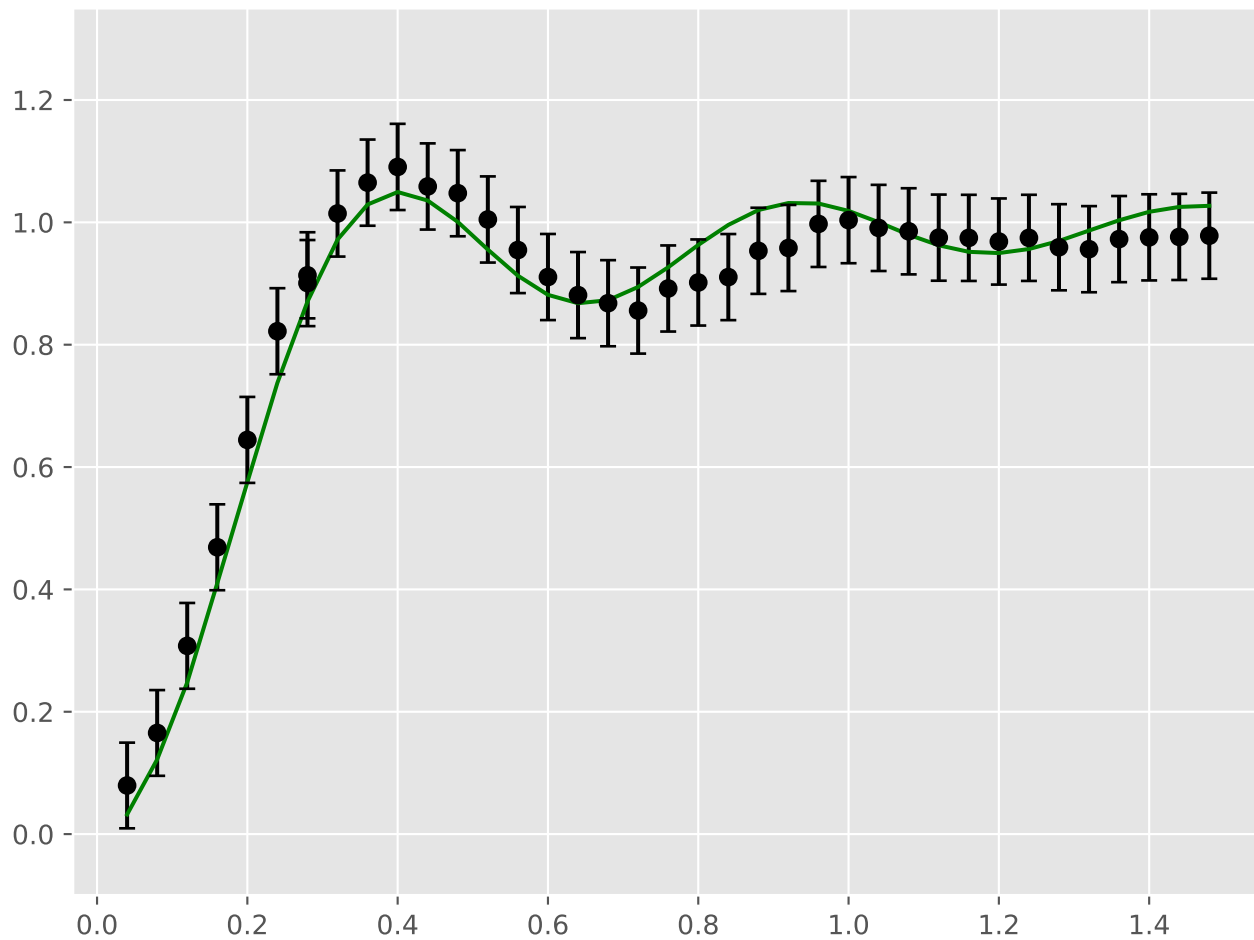

Supplement: Supplementary file 4 — Source data [file 41467_2022_29423_MOESM4_ESM.gz › source_data_2022/Figures_2B-E_SI5_SI6_SI7/Methyl dipolar-coupling measurements. Exptl data, simulations and analysis scripts/fit-figures/193.pdf]

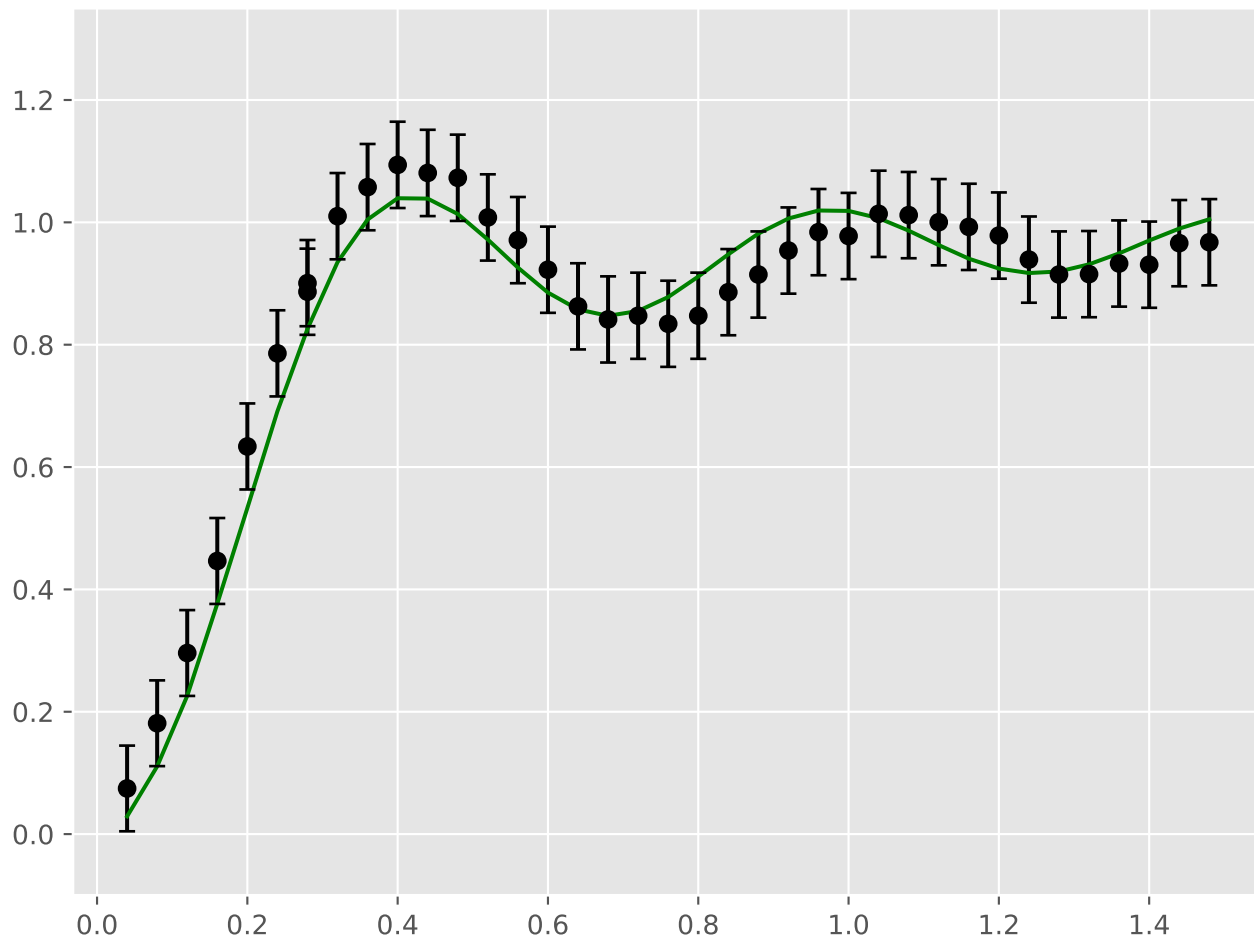

Supplement: Supplementary file 4 — Source data [file 41467_2022_29423_MOESM4_ESM.gz › source_data_2022/Figures_2B-E_SI5_SI6_SI7/Methyl dipolar-coupling measurements. Exptl data, simulations and analysis scripts/fit-figures/197.pdf]

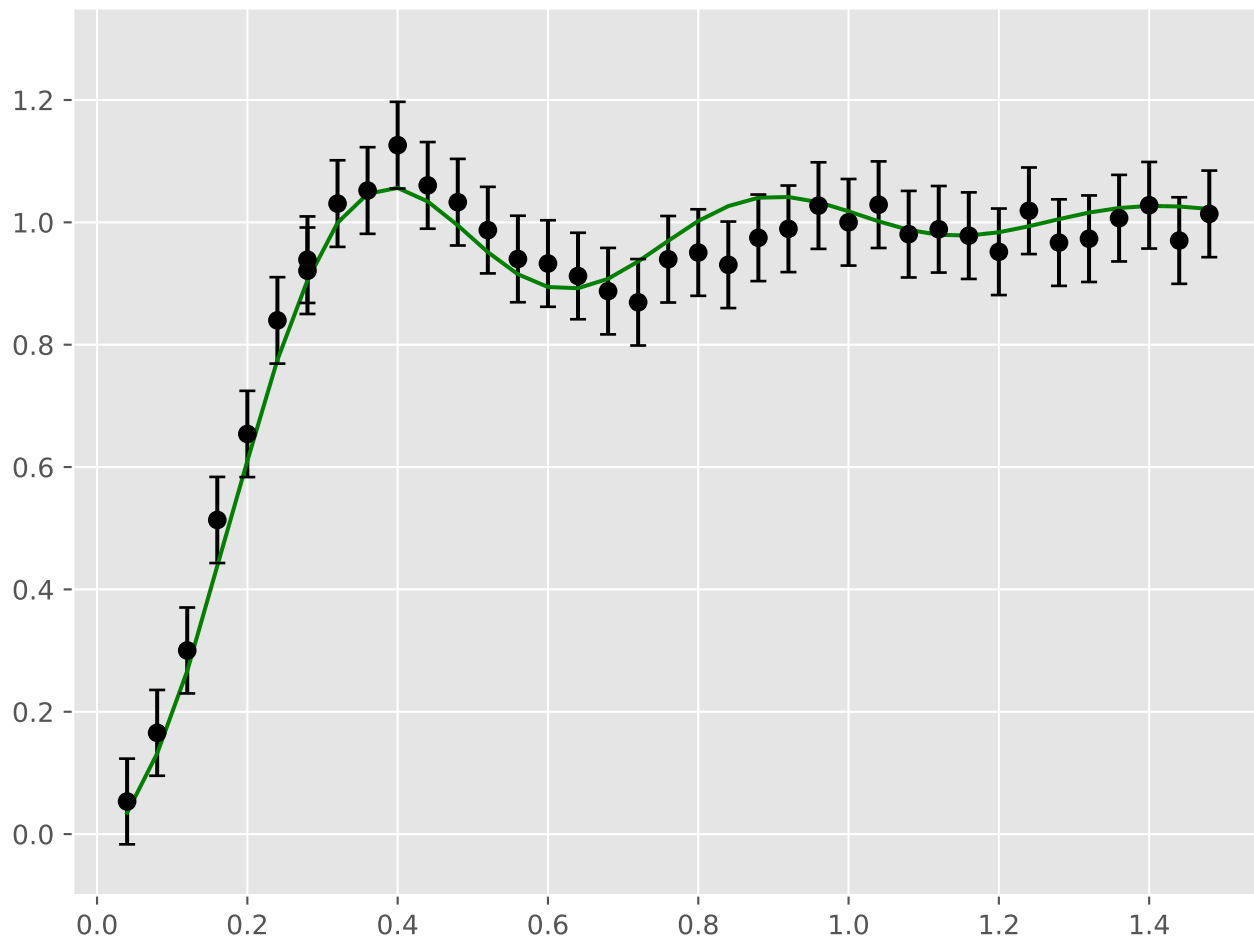

Supplement: Supplementary file 4 — Source data [file 41467_2022_29423_MOESM4_ESM.gz › source_data_2022/Figures_2B-E_SI5_SI6_SI7/Methyl dipolar-coupling measurements. Exptl data, simulations and analysis scripts/fit-figures/179.pdf]

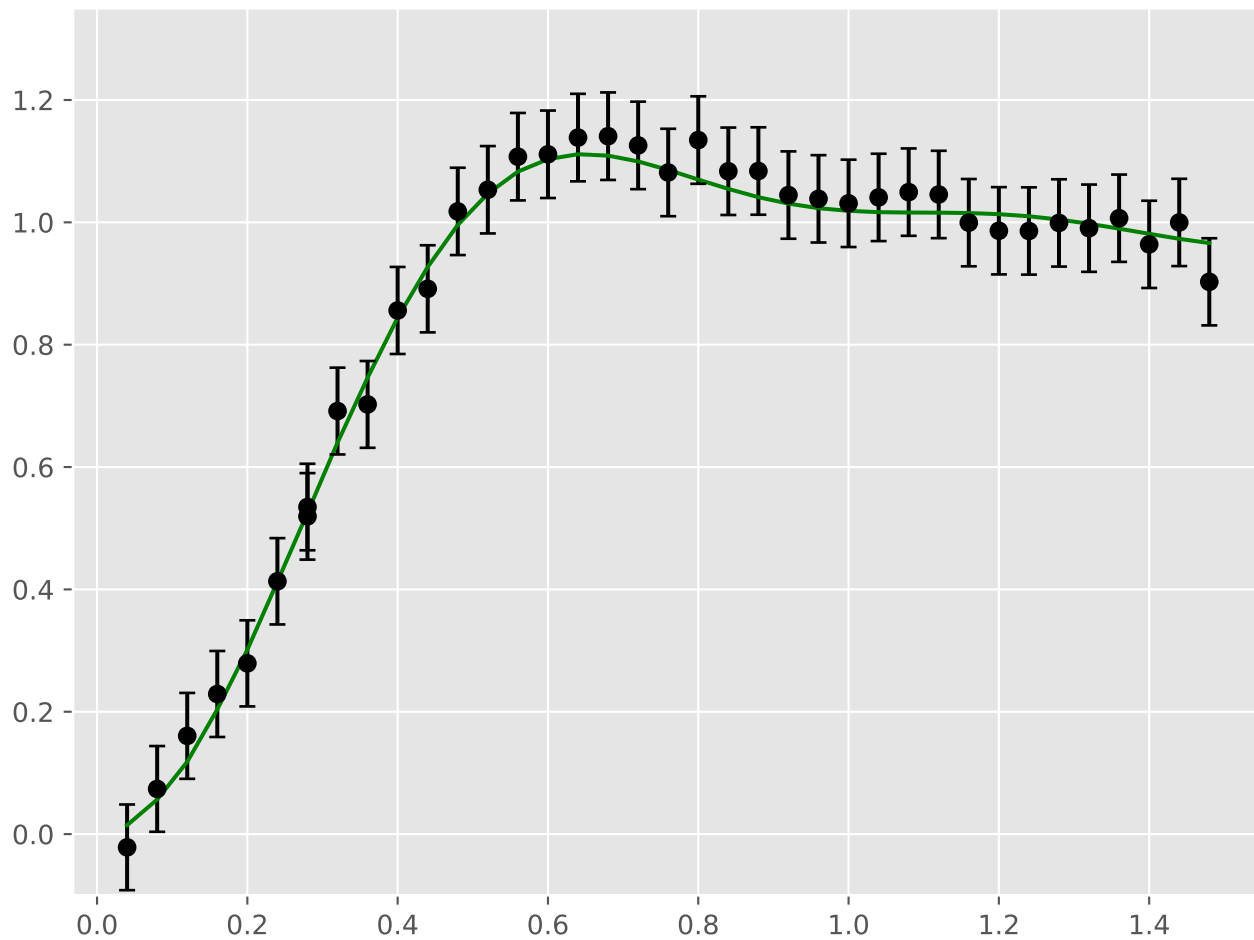

Supplement: Supplementary file 4 — Source data [file 41467_2022_29423_MOESM4_ESM.gz › source_data_2022/Figures_2B-E_SI5_SI6_SI7/Methyl dipolar-coupling measurements. Exptl data, simulations and analysis scripts/fit-figures/214.pdf]

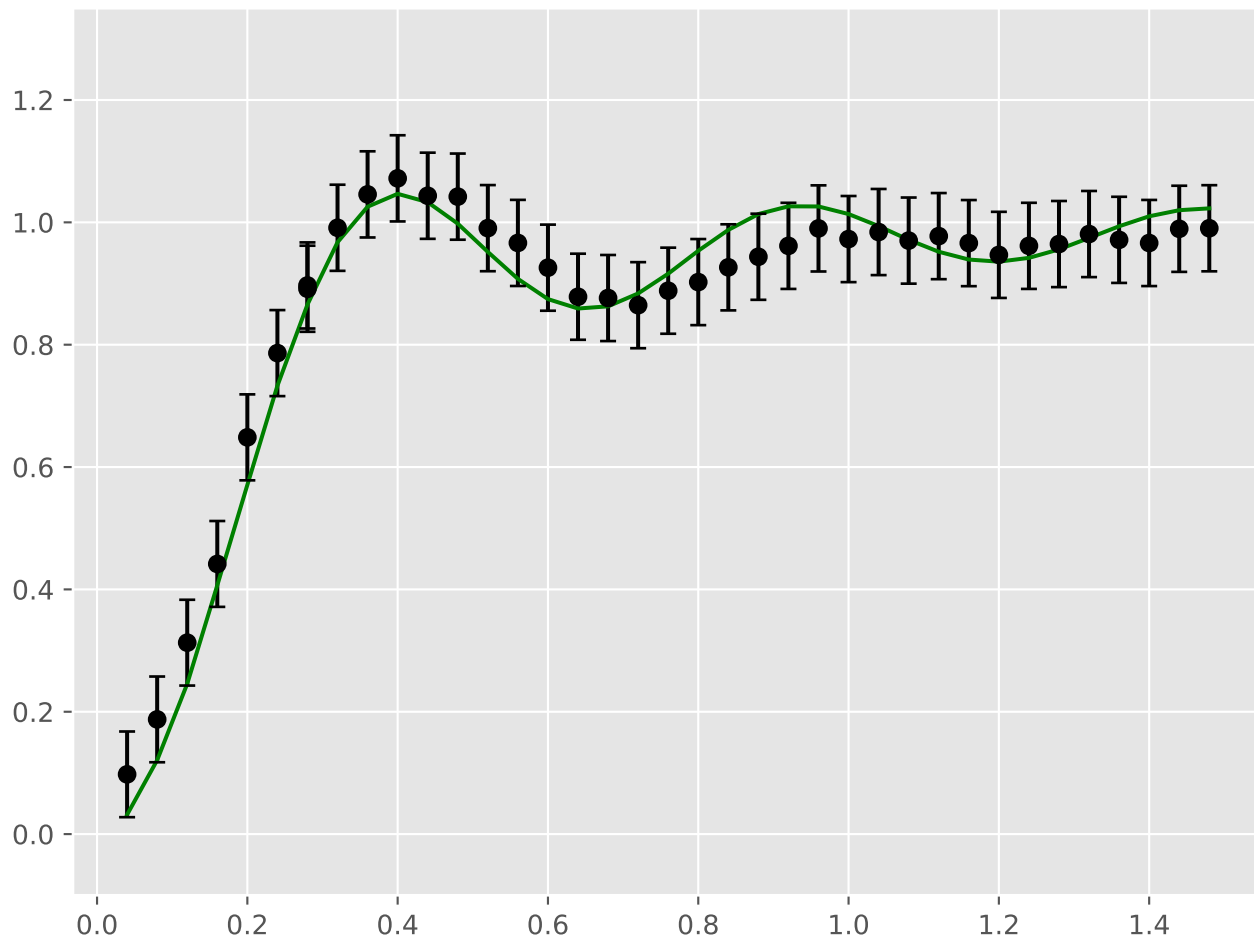

Supplement: Supplementary file 4 — Source data [file 41467_2022_29423_MOESM4_ESM.gz › source_data_2022/Figures_2B-E_SI5_SI6_SI7/Methyl dipolar-coupling measurements. Exptl data, simulations and analysis scripts/fit-figures/207.pdf]

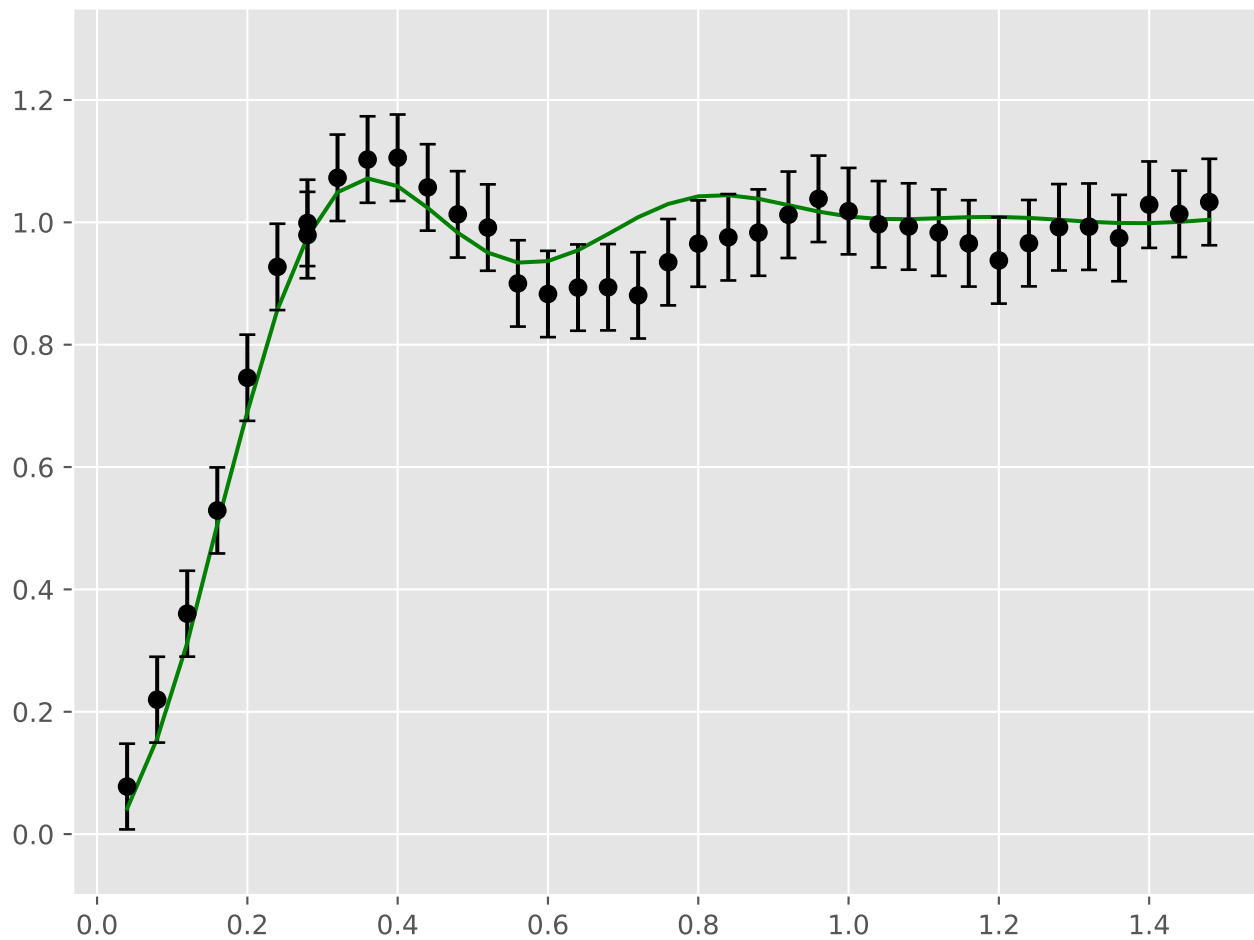

Supplement: Supplementary file 4 — Source data [file 41467_2022_29423_MOESM4_ESM.gz › source_data_2022/Figures_2B-E_SI5_SI6_SI7/Methyl dipolar-coupling measurements. Exptl data, simulations and analysis scripts/fit-figures/210.pdf]

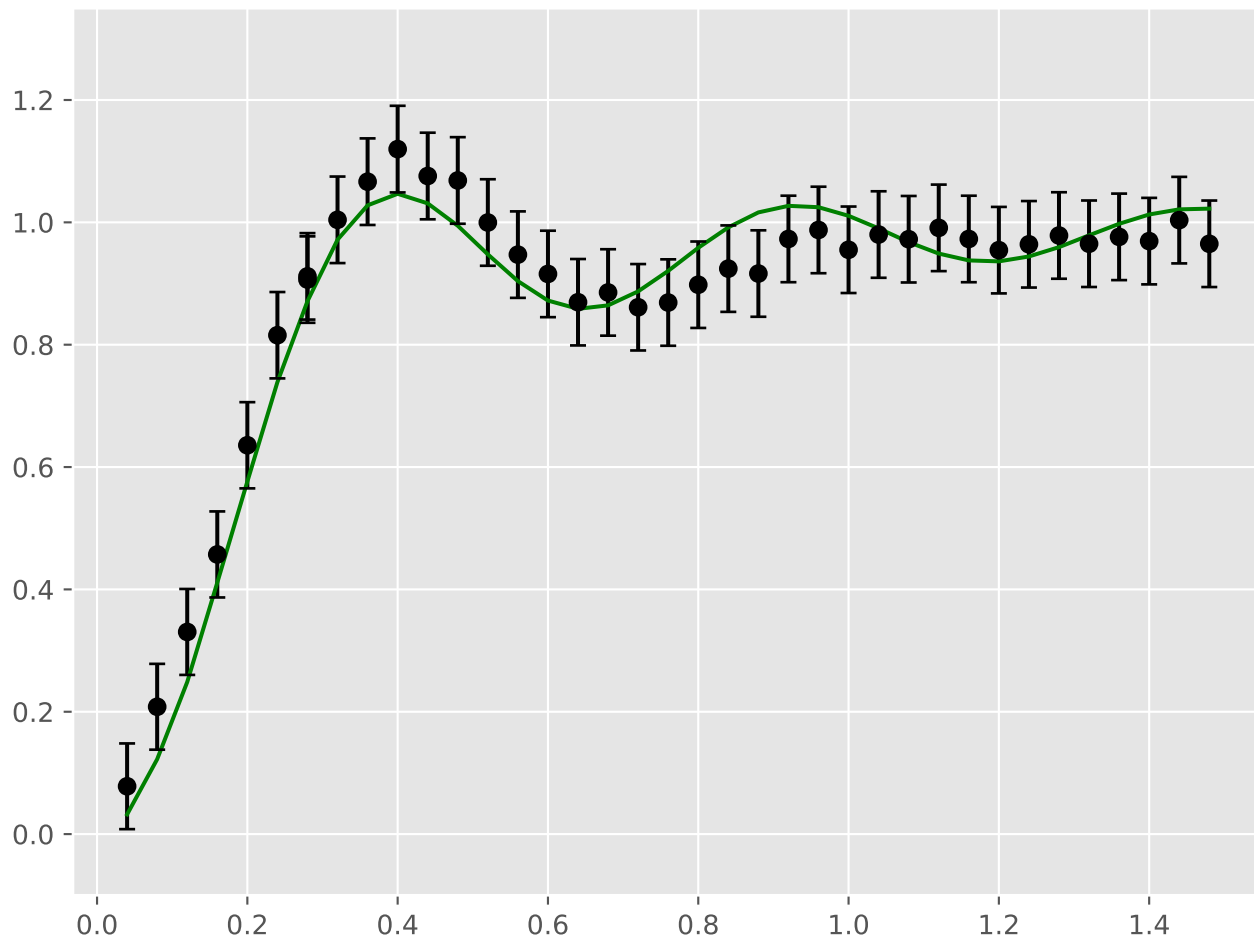

Supplement: Supplementary file 4 — Source data [file 41467_2022_29423_MOESM4_ESM.gz › source_data_2022/Figures_2B-E_SI5_SI6_SI7/Methyl dipolar-coupling measurements. Exptl data, simulations and analysis scripts/fit-figures/226.pdf]

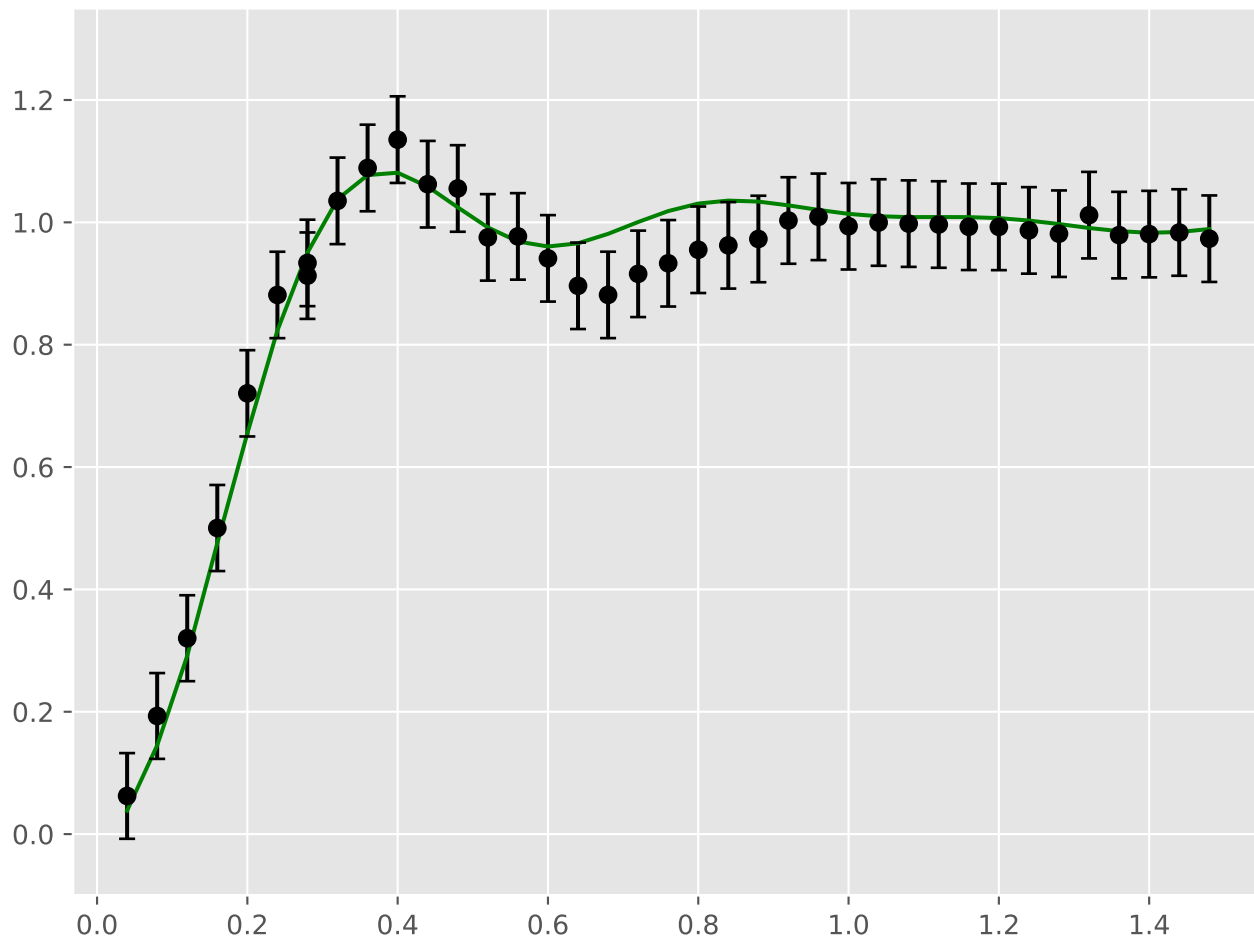

Supplement: Supplementary file 4 — Source data [file 41467_2022_29423_MOESM4_ESM.gz › source_data_2022/Figures_2B-E_SI5_SI6_SI7/Methyl dipolar-coupling measurements. Exptl data, simulations and analysis scripts/fit-figures/267.pdf]

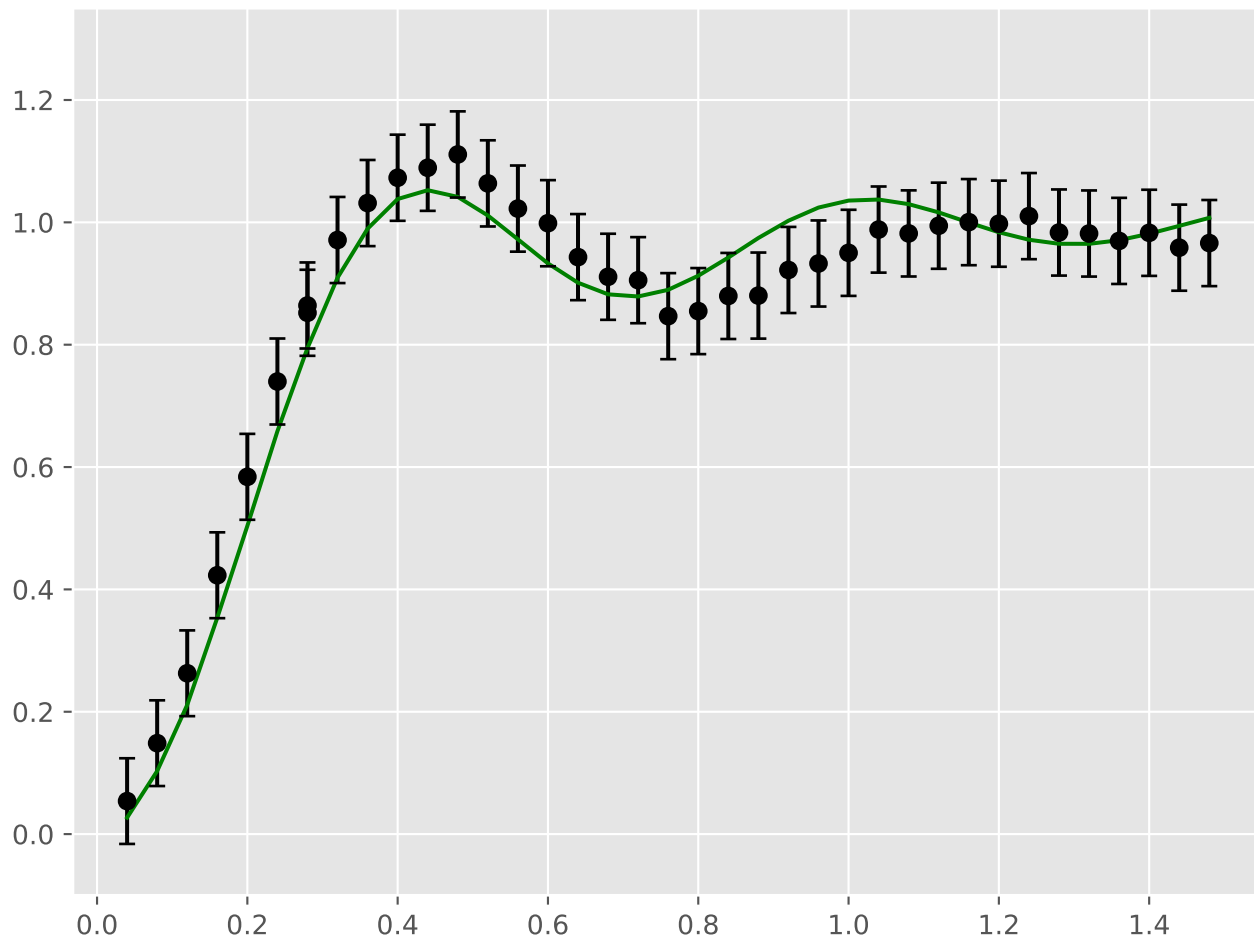

Supplement: Supplementary file 4 — Source data [file 41467_2022_29423_MOESM4_ESM.gz › source_data_2022/Figures_2B-E_SI5_SI6_SI7/Methyl dipolar-coupling measurements. Exptl data, simulations and analysis scripts/fit-figures/262.pdf]

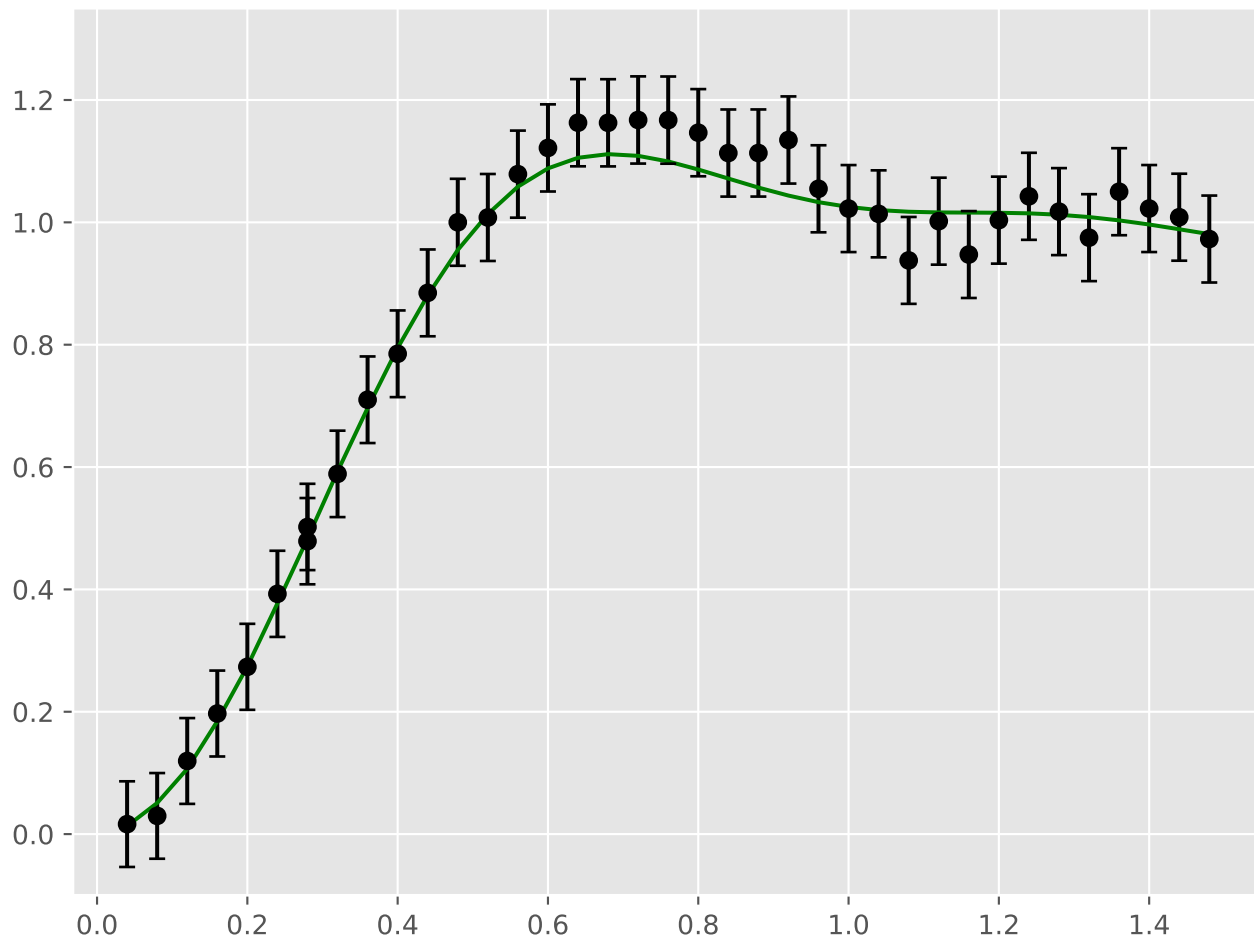

Supplement: Supplementary file 4 — Source data [file 41467_2022_29423_MOESM4_ESM.gz › source_data_2022/Figures_2B-E_SI5_SI6_SI7/Methyl dipolar-coupling measurements. Exptl data, simulations and analysis scripts/fit-figures/27.pdf]

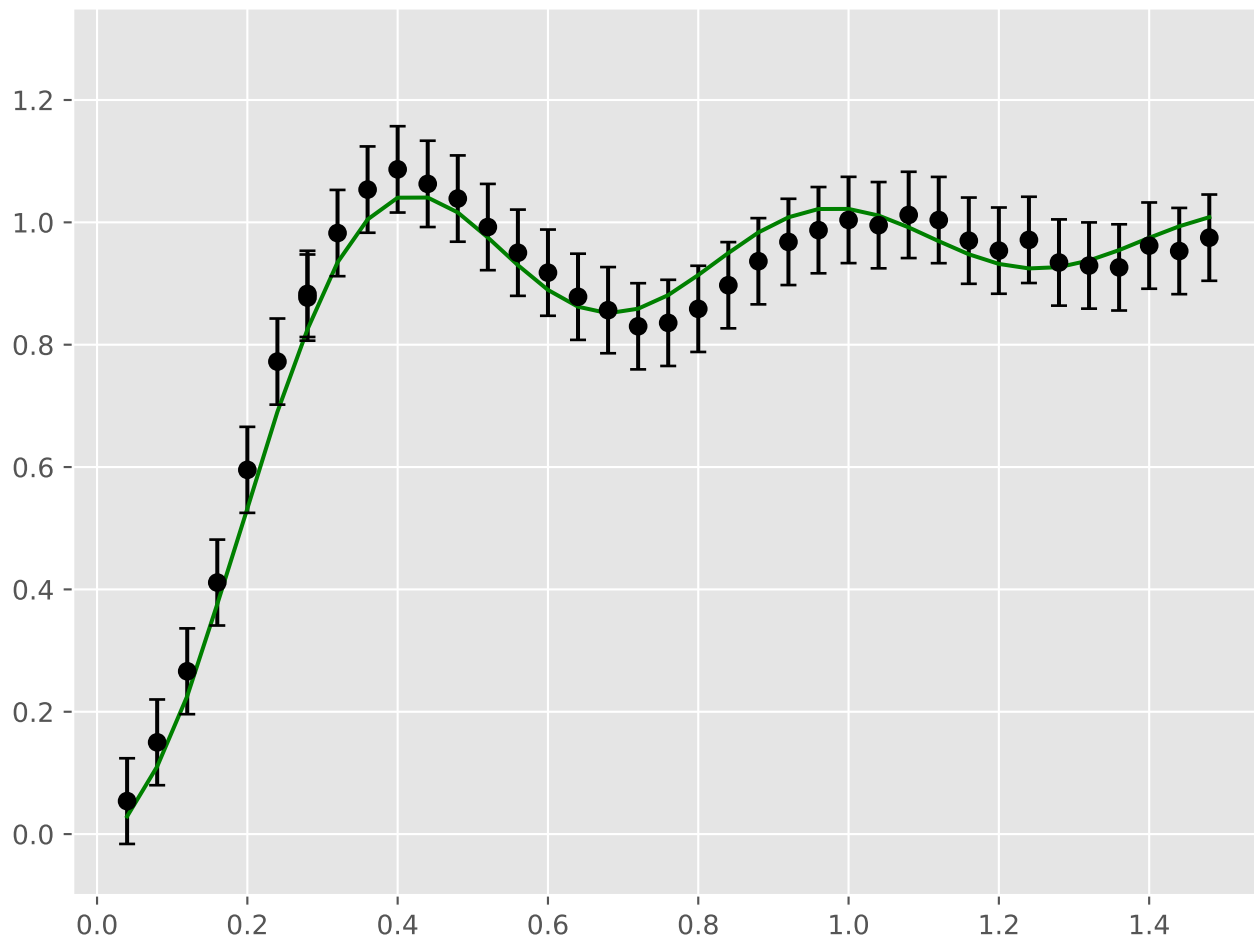

Supplement: Supplementary file 4 — Source data [file 41467_2022_29423_MOESM4_ESM.gz › source_data_2022/Figures_2B-E_SI5_SI6_SI7/Methyl dipolar-coupling measurements. Exptl data, simulations and analysis scripts/fit-figures/260.pdf]

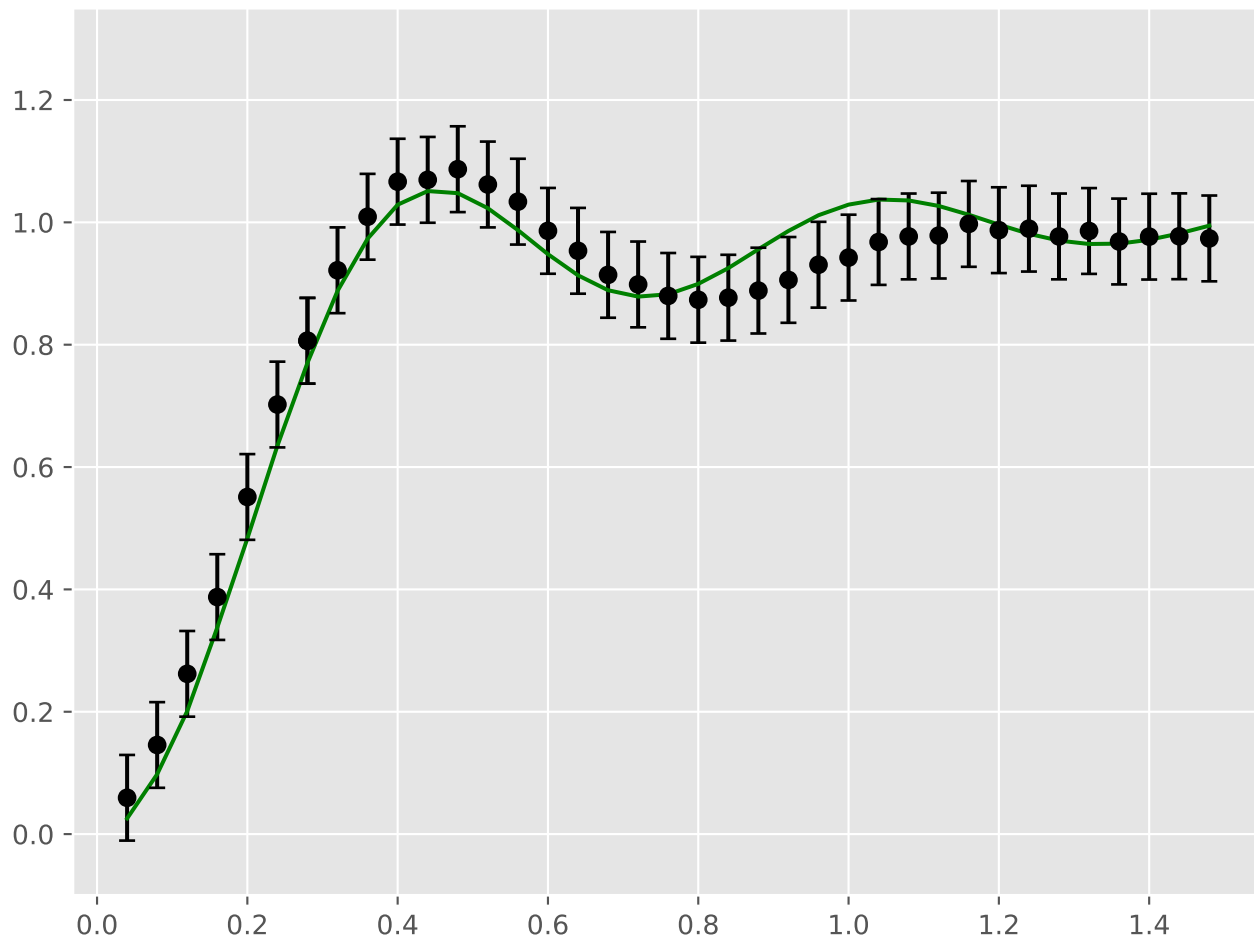

Supplement: Supplementary file 4 — Source data [file 41467_2022_29423_MOESM4_ESM.gz › source_data_2022/Figures_2B-E_SI5_SI6_SI7/Methyl dipolar-coupling measurements. Exptl data, simulations and analysis scripts/fit-figures/303.pdf]

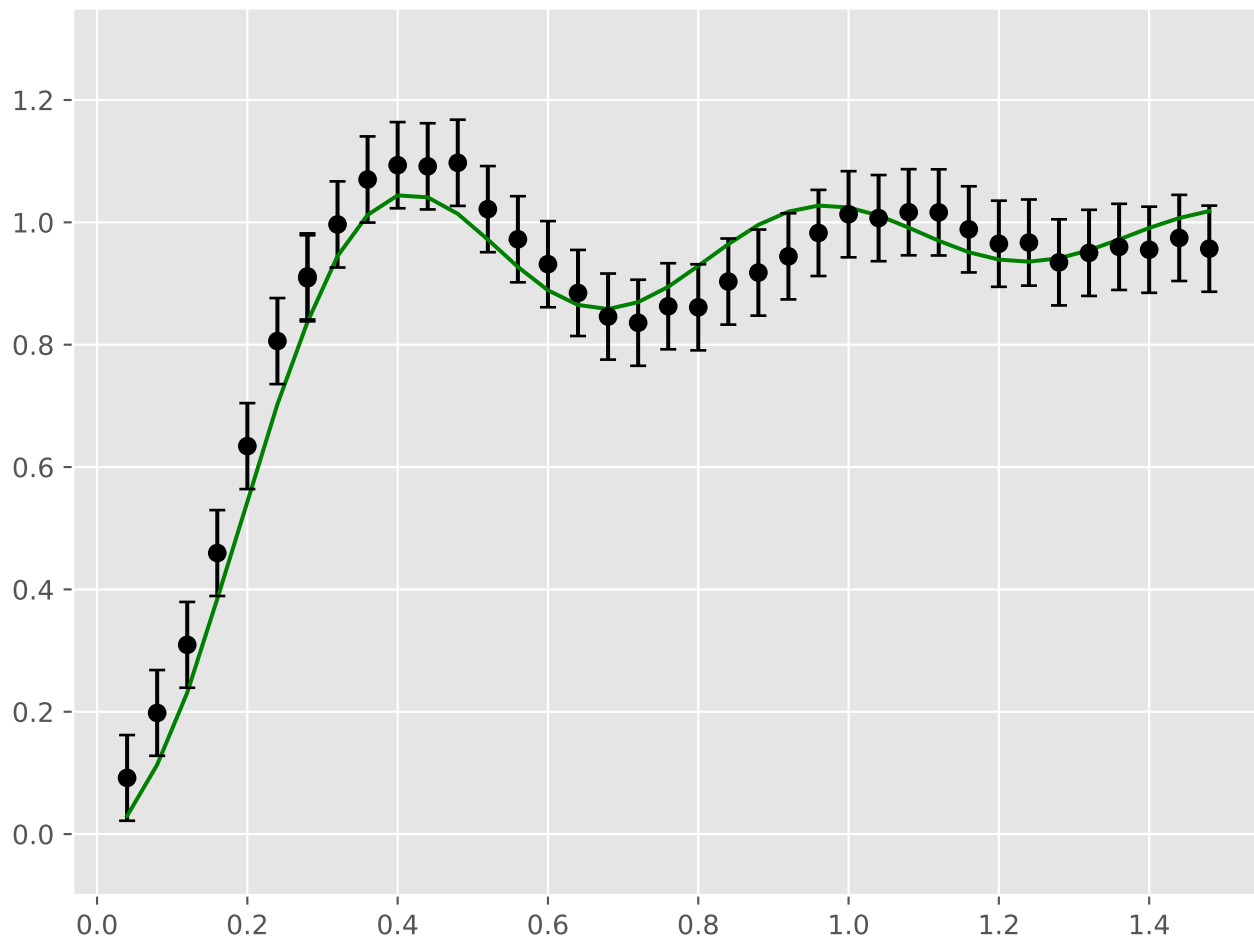

Supplement: Supplementary file 4 — Source data [file 41467_2022_29423_MOESM4_ESM.gz › source_data_2022/Figures_2B-E_SI5_SI6_SI7/Methyl dipolar-coupling measurements. Exptl data, simulations and analysis scripts/fit-figures/33.pdf]

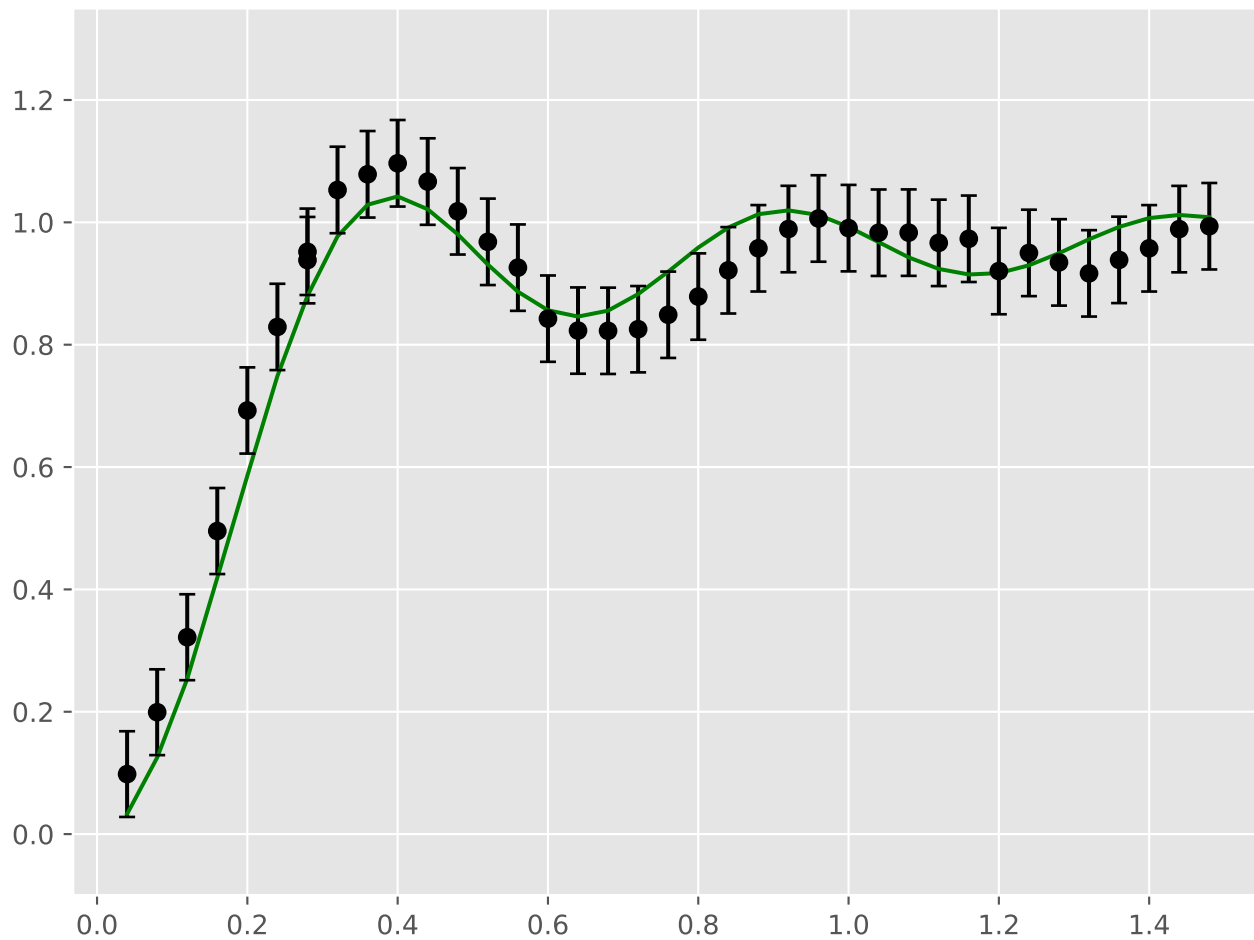

Supplement: Supplementary file 4 — Source data [file 41467_2022_29423_MOESM4_ESM.gz › source_data_2022/Figures_2B-E_SI5_SI6_SI7/Methyl dipolar-coupling measurements. Exptl data, simulations and analysis scripts/fit-figures/317.pdf]

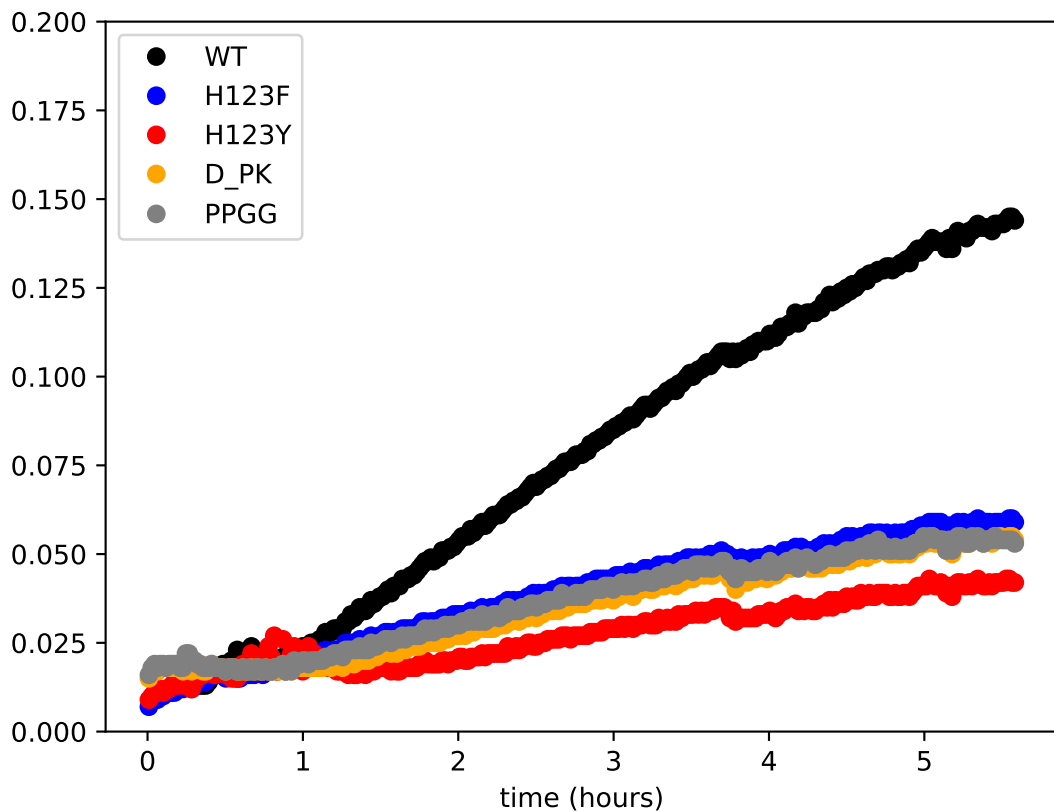

Supplement: Supplementary file 4 — Source data [file 41467_2022_29423_MOESM4_ESM.gz › source_data_2022/figure_4B-C/4C/1mM_H-LEU-VAL-LEU-ALA-pNA.pdf]

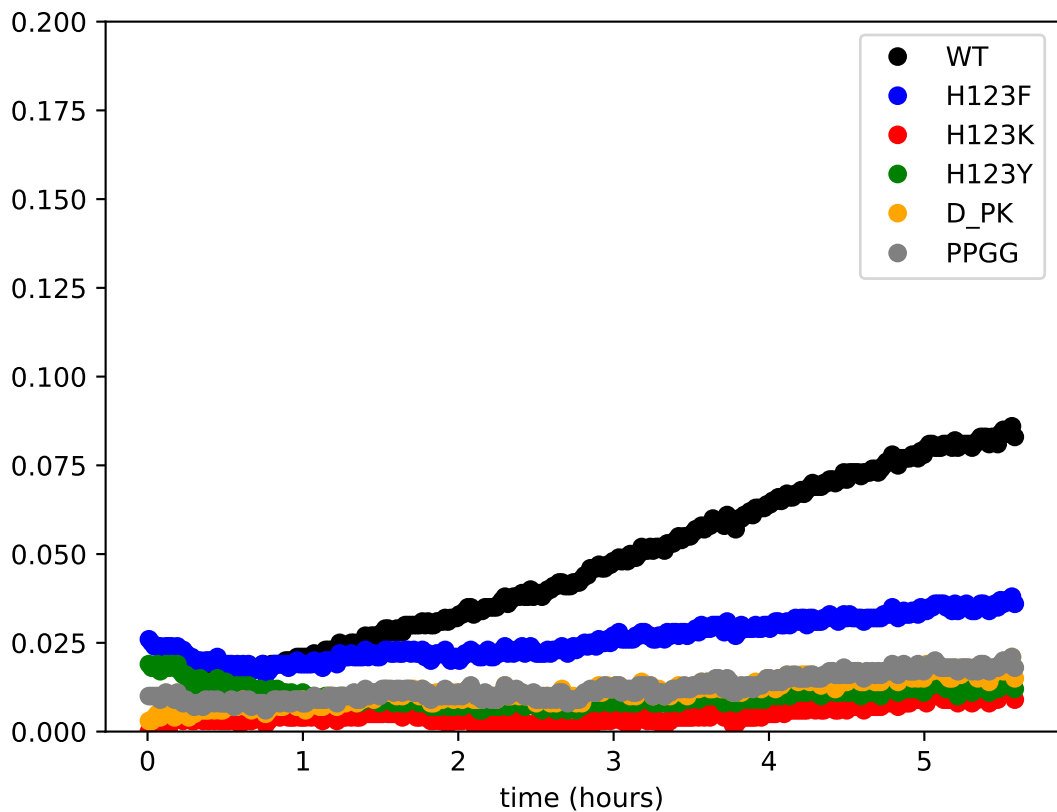

Supplement: Supplementary file 4 — Source data [file 41467_2022_29423_MOESM4_ESM.gz › source_data_2022/figure_4B-C/4C/0.5mM_H-LEU-VAL-LEU-ALA-pNA.pdf]
