# Supplementary material for: Functional control of a 0.5 MDa TET aminopeptidase by a flexible loop revealed by MAS NMR
Source: Nat Commun. 2022 Apr 8;13:1927. doi: 10.1038/s41467-022-29423-0 (PMC8993905; doi:10.1038/s41467-022-29423-0)
Supplement: Supplementary file 4 — Source data [file 41467_2022_29423_MOESM4_ESM.gz › source_data_2022/figures_SI17_SI18/S17-18/activity_freeHis_Iva.zip!/activity_freeHis_Iva/TET2_wt_mutants_pH_graphs.pdf]

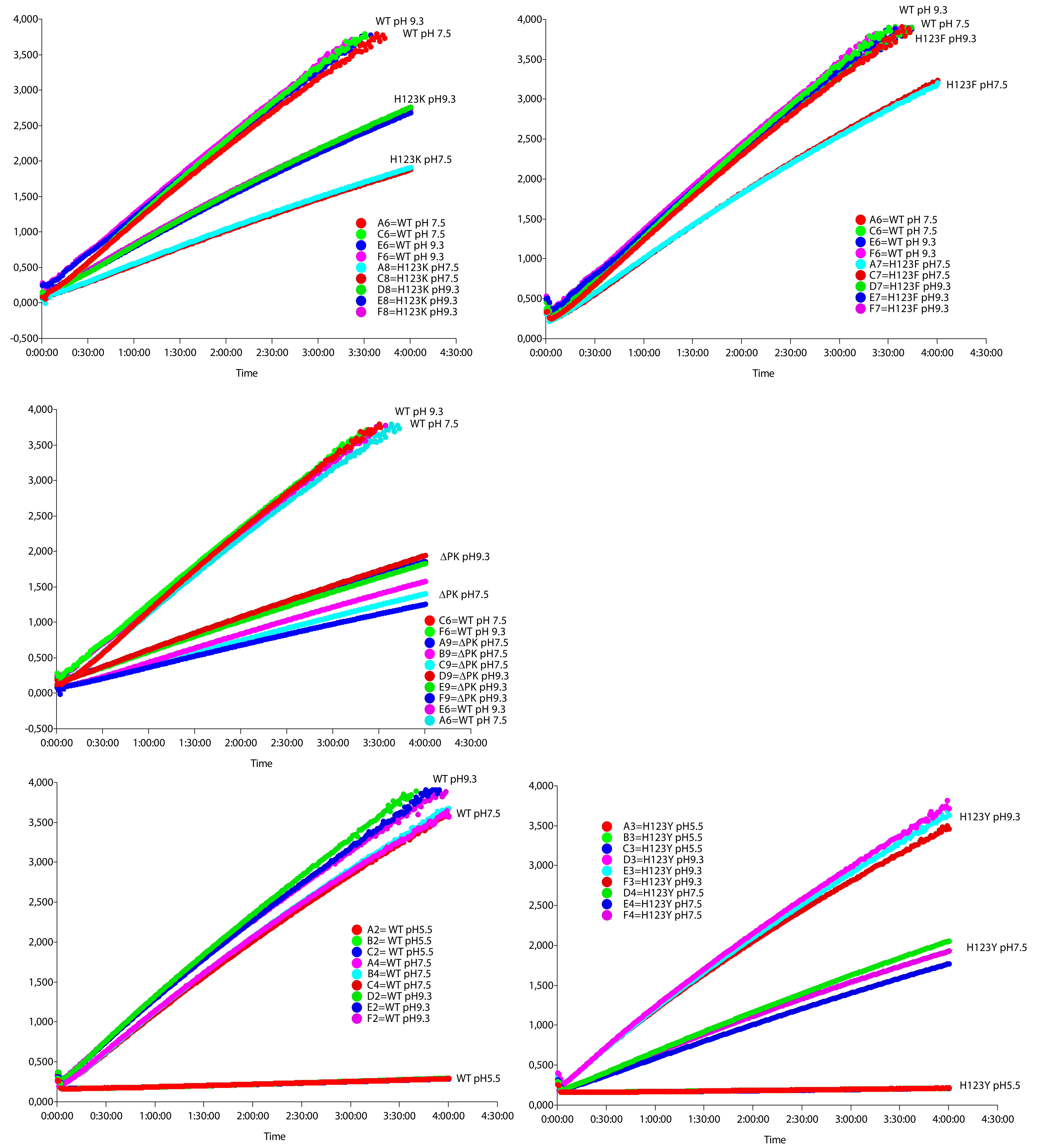
WT pH 9.3
WT pH 7.5
H123K pH9.3
H123K pH7.5
A6=WT pH 7.5
C6=WT pH 7.5
E6=WT pH 9.3
F6=WT pH 9.3
A8=H123K pH7.5
C8=H123K pH7.5
D8=H123K pH9.3
E8=H123K pH9.3
F8=H123K pH9.3
Time
WT pH 9.3
WT pH 7.5
H123F pH9.3
H123F pH7.5
A6=WT pH 7.5
C6=WT pH 7.5
E6=WT pH 9.3
F6=WT pH 9.3
A7=H123F pH7.5
C7=H123F pH7.5
D7=H123F pH9.3
E7=H123F pH9.3
F7=H123F pH9.3
Time
WT pH 9.3
WT pH 7.5
ΔPK pH9.3
ΔPK pH7.5
C6=WT pH 7.5
F6=WT pH 9.3
A9=ΔPK pH7.5
B9=ΔPK pH7.5
C9=ΔPK pH7.5
D9=ΔPK pH9.3
E9=ΔPK pH9.3
F9=ΔPK pH9.3
E6=WT pH 9.3
A6=WT pH 7.5
Time
WT pH9.3
WT pH7.5
WT pH5.5
A2= WT pH5.5
B2= WT pH5.5
C2= WT pH5.5
A4=WT pH7.5
B4=WT pH7.5
C4=WT pH7.5
D2=WT pH9.3
E2=WT pH9.3
F2=WT pH9.3
Time
A3=H123Y pH5.5
B3=H123Y pH5.5
C3=H123Y pH5.5
D3=H123Y pH9.3
E3=H123Y pH9.3
F3=H123Y pH9.3
D4=H123Y pH7.5
E4=H123Y pH7.5
F4=H123Y pH7.5
H123Y pH9.3
H123Y pH7.5
H123Y pH5.5
Time
