# Supplementary material for: Functional control of a 0.5 MDa TET aminopeptidase by a flexible loop revealed by MAS NMR
Source: Nat Commun. 2022 Apr 8;13:1927. doi: 10.1038/s41467-022-29423-0 (PMC8993905; doi:10.1038/s41467-022-29423-0)
Supplement: Supplementary file 4 — Source data [file 41467_2022_29423_MOESM4_ESM.gz › source_data_2022/figures_SI17_SI18/S17-18/activity_freeHis_Iva.zip!/activity_freeHis_Iva/iva2309/20210923Experiment1 - Report1.pdf]

Software Version	3.11.19

Experiment File Path:	D:\data\2021\iva2309\Experiment1.xpt
Protocol File Path:	D:\data\2021\iva2309\Protocol1.prt

Plate Number	Plate 1
Date	23/09/2021
Time	13:35:13
Reader Type:	Synergy H4
Reader Serial Number:	257169
Reading Type	Reader

Procedure Details

Plate Type	96 WELL PLATE (Use plate lid)
Eject plate on completion	
Set Temperature	Setpoint 50°C
	Preheat before moving to next step
Delay	0:20:00 (HH:MM:SS)
Plate Out/In	add substrate
Shake	Medium, 0:10 (MM:SS)
Start Kinetic	Runtime 4:00:00 (HH:MM:SS), Interval 0:01:00, 241 Reads
Read	Absorbance Endpoint
	Full Plate
	Wavelengths: 410
	Read Speed: Normal, Delay: 100 msec, Measurements/Data Point: 8
End Kinetic	

Layout

	1	2	3	4	5	6	7	8	9	10	11	12	
A	BLK	SPL1											Well ID
B		SPL1											Well ID
C		SPL1											Well ID
D		SPL2											Well ID
E		SPL2											Well ID
F		SPL2											Well ID
G													Well ID
H													Well ID

410

Time	T° 410	A1	A2	B2	C2	D2	E2	F2
0:00:12	49,9	0,135	0,158	0,163	0,170	0,167	0,173	0,173
0:01:12	49,9	0,106	0,157	0,156	0,176	0,172	0,180	0,180
0:02:12	49,9	0,106	0,157	0,156	0,187	0,177	0,185	0,187
0:03:12	49,9	0,106	0,157	0,163	0,199	0,182	0,191	0,193

Time	T° 410	A1	A2	B2	C2	D2	E2	F2
0:04:12	49,9	0,106	0,157	0,167	0,211	0,185	0,196	0,199
0:05:12	49,9	0,106	0,158	0,175	0,227	0,189	0,201	0,202
0:06:12	50,0	0,106	0,158	0,185	0,243	0,191	0,204	0,206
0:07:12	50,0	0,106	0,158	0,197	0,259	0,194	0,208	0,209
0:08:12	49,9	0,106	0,158	0,211	0,274	0,196	0,211	0,212
0:09:12	49,9	0,106	0,158	0,229	0,290	0,199	0,213	0,215
0:10:12	49,9	0,106	0,158	0,246	0,305	0,200	0,215	0,217
0:11:12	49,9	0,106	0,158	0,267	0,320	0,202	0,218	0,219
0:12:12	49,9	0,106	0,158	0,284	0,334	0,203	0,220	0,221
0:13:12	49,9	0,109	0,158	0,298	0,349	0,205	0,221	0,223
0:14:12	50,0	0,110	0,159	0,314	0,365	0,206	0,223	0,225
0:15:12	50,0	0,112	0,158	0,328	0,380	0,207	0,224	0,227
0:16:12	50,0	0,112	0,159	0,344	0,396	0,208	0,226	0,229
0:17:12	49,9	0,111	0,159	0,359	0,411	0,209	0,227	0,229
0:18:12	49,9	0,113	0,159	0,374	0,426	0,210	0,229	0,230
0:19:12	49,9	0,113	0,159	0,389	0,442	0,211	0,230	0,232
0:20:12	49,9	0,111	0,159	0,404	0,457	0,212	0,231	0,232
0:21:12	50,0	0,113	0,159	0,419	0,472	0,213	0,232	0,233
0:22:12	49,9	0,112	0,159	0,434	0,488	0,213	0,233	0,234
0:23:12	49,9	0,110	0,159	0,450	0,503	0,214	0,234	0,235
0:24:12	50,0	0,113	0,159	0,465	0,519	0,215	0,235	0,236
0:25:12	50,0	0,113	0,159	0,480	0,534	0,215	0,236	0,237
0:26:12	50,0	0,112	0,159	0,495	0,550	0,216	0,237	0,237
0:27:12	50,0	0,110	0,159	0,511	0,566	0,216	0,237	0,238
0:28:12	50,0	0,109	0,159	0,526	0,581	0,217	0,238	0,239
0:29:12	50,0	0,109	0,159	0,541	0,596	0,218	0,239	0,240
0:30:12	50,0	0,108	0,159	0,556	0,612	0,218	0,239	0,240
0:31:12	50,0	0,108	0,159	0,571	0,627	0,219	0,240	0,241
0:32:12	50,0	0,108	0,160	0,587	0,643	0,219	0,241	0,242
0:33:12	50,0	0,110	0,160	0,602	0,658	0,220	0,242	0,243
0:34:12	49,9	0,111	0,160	0,617	0,673	0,220	0,242	0,244
0:35:12	49,9	0,112	0,160	0,632	0,689	0,221	0,242	0,244
0:36:12	50,0	0,112	0,160	0,648	0,704	0,221	0,243	0,245
0:37:12	49,9	0,112	0,160	0,663	0,720	0,222	0,243	0,245
0:38:12	49,9	0,112	0,160	0,678	0,735	0,222	0,244	0,246
0:39:12	49,9	0,112	0,160	0,693	0,750	0,222	0,244	0,246
0:40:12	49,9	0,113	0,160	0,709	0,765	0,223	0,245	0,246
0:41:12	49,9	0,113	0,160	0,723	0,780	0,223	0,245	0,247
0:42:12	49,9	0,113	0,160	0,739	0,795	0,223	0,246	0,247
0:43:12	49,9	0,113	0,160	0,753	0,811	0,224	0,246	0,248
0:44:12	49,9	0,113	0,160	0,769	0,826	0,224	0,247	0,248
0:45:12	49,9	0,113	0,160	0,784	0,841	0,224	0,247	0,249
0:46:12	50,0	0,113	0,160	0,799	0,856	0,225	0,248	0,249
0:47:12	50,0	0,113	0,160	0,814	0,871	0,225	0,248	0,250
0:48:12	50,0	0,113	0,160	0,829	0,887	0,225	0,248	0,250
0:49:12	50,0	0,113	0,160	0,844	0,902	0,226	0,249	0,250
0:50:12	50,0	0,113	0,160	0,859	0,917	0,226	0,249	0,251
0:51:12	50,0	0,112	0,160	0,874	0,932	0,226	0,250	0,251

Time	T° 410	A1	A2	B2	C2	D2	E2	F2
0:52:12	50,0	0,112	0,160	0,889	0,947	0,226	0,250	0,251
0:53:12	50,0	0,112	0,160	0,904	0,962	0,227	0,251	0,252
0:54:12	50,0	0,112	0,160	0,919	0,977	0,227	0,251	0,252
0:55:12	49,9	0,112	0,160	0,934	0,992	0,227	0,251	0,252
0:56:12	50,0	0,111	0,160	0,949	1,007	0,228	0,251	0,253
0:57:12	49,9	0,111	0,160	0,964	1,022	0,228	0,252	0,253
0:58:12	49,9	0,111	0,160	0,979	1,037	0,228	0,252	0,253
0:59:12	49,9	0,111	0,160	0,993	1,052	0,228	0,253	0,254
1:00:12	49,9	0,111	0,161	1,008	1,067	0,229	0,253	0,254
1:01:12	49,9	0,111	0,160	1,023	1,082	0,229	0,253	0,255
1:02:12	49,9	0,111	0,161	1,038	1,097	0,229	0,254	0,255
1:03:12	50,0	0,111	0,160	1,053	1,112	0,229	0,254	0,255
1:04:12	50,0	0,111	0,161	1,067	1,127	0,229	0,254	0,256
1:05:12	50,0	0,111	0,161	1,082	1,141	0,230	0,258	0,256
1:06:12	50,0	0,111	0,161	1,097	1,156	0,230	0,260	0,256
1:07:12	49,9	0,111	0,161	1,111	1,171	0,230	0,259	0,256
1:08:12	49,9	0,111	0,161	1,126	1,185	0,230	0,259	0,256
1:09:12	49,9	0,111	0,161	1,140	1,200	0,230	0,260	0,257
1:10:12	49,9	0,111	0,161	1,155	1,215	0,230	0,259	0,257
1:11:12	50,0	0,111	0,161	1,169	1,230	0,231	0,261	0,257
1:12:12	50,0	0,111	0,161	1,184	1,245	0,231	0,260	0,257
1:13:12	50,0	0,111	0,161	1,199	1,259	0,231	0,260	0,257
1:14:12	50,0	0,111	0,161	1,213	1,274	0,231	0,260	0,257
1:15:12	50,0	0,110	0,161	1,228	1,288	0,231	0,261	0,257
1:16:12	50,0	0,111	0,161	1,242	1,303	0,232	0,261	0,258
1:17:12	50,0	0,110	0,161	1,256	1,317	0,232	0,260	0,258
1:18:12	50,0	0,110	0,161	1,271	1,332	0,232	0,261	0,258
1:19:12	50,0	0,110	0,161	1,286	1,346	0,232	0,262	0,258
1:20:12	50,0	0,110	0,161	1,300	1,361	0,232	0,261	0,258
1:21:12	50,0	0,110	0,161	1,314	1,375	0,232	0,261	0,259
1:22:12	50,0	0,109	0,161	1,328	1,389	0,232	0,261	0,259
1:23:12	50,0	0,109	0,161	1,343	1,404	0,232	0,261	0,259
1:24:12	50,0	0,109	0,161	1,357	1,418	0,233	0,261	0,259
1:25:12	50,0	0,109	0,161	1,370	1,432	0,233	0,262	0,259
1:26:12	49,9	0,109	0,161	1,385	1,446	0,233	0,262	0,259
1:27:12	50,0	0,109	0,161	1,399	1,461	0,233	0,263	0,260
1:28:12	50,0	0,109	0,161	1,413	1,475	0,233	0,263	0,260
1:29:12	49,9	0,109	0,161	1,428	1,489	0,233	0,263	0,259
1:30:12	50,0	0,108	0,161	1,441	1,504	0,233	0,263	0,260
1:31:12	50,0	0,108	0,161	1,456	1,518	0,233	0,263	0,259
1:32:12	50,0	0,108	0,161	1,470	1,532	0,233	0,264	0,260
1:33:12	50,0	0,108	0,161	1,484	1,546	0,234	0,264	0,260
1:34:12	50,0	0,108	0,162	1,497	1,560	0,234	0,264	0,260
1:35:12	50,0	0,107	0,161	1,511	1,574	0,234	0,263	0,260
1:36:12	49,9	0,108	0,162	1,526	1,589	0,234	0,264	0,260
1:37:12	49,9	0,107	0,162	1,539	1,602	0,234	0,264	0,260
1:38:12	49,9	0,107	0,162	1,553	1,617	0,234	0,264	0,260
1:39:12	49,9	0,107	0,161	1,567	1,630	0,234	0,264	0,260

Time	T° 410	A1	A2	B2	C2	D2	E2	F2
1:40:12	49,9	0,107	0,162	1,581	1,644	0,234	0,264	0,260
1:41:12	49,9	0,107	0,162	1,595	1,659	0,234	0,264	0,262
1:42:12	49,9	0,107	0,162	1,608	1,671	0,234	0,264	0,260
1:43:12	50,0	0,107	0,162	1,622	1,686	0,234	0,264	0,260
1:44:12	49,9	0,107	0,162	1,636	1,700	0,235	0,264	0,260
1:45:12	49,9	0,107	0,162	1,650	1,714	0,234	0,264	0,261
1:46:12	49,9	0,109	0,162	1,664	1,728	0,235	0,264	0,261
1:47:12	49,9	0,113	0,162	1,677	1,741	0,235	0,264	0,261
1:48:12	49,9	0,113	0,162	1,690	1,755	0,235	0,264	0,261
1:49:12	49,9	0,113	0,162	1,704	1,768	0,235	0,264	0,261
1:50:12	50,0	0,113	0,162	1,718	1,782	0,235	0,264	0,261
1:51:12	50,0	0,113	0,162	1,731	1,796	0,235	0,264	0,261
1:52:12	50,0	0,111	0,162	1,745	1,810	0,235	0,264	0,261
1:53:12	50,0	0,113	0,162	1,759	1,823	0,235	0,264	0,261
1:54:12	50,0	0,109	0,162	1,772	1,836	0,235	0,264	0,261
1:55:12	49,9	0,110	0,162	1,786	1,851	0,235	0,264	0,261
1:56:12	49,9	0,110	0,162	1,798	1,863	0,235	0,264	0,261
1:57:12	49,9	0,111	0,162	1,813	1,876	0,235	0,265	0,261
1:58:12	50,0	0,113	0,162	1,825	1,891	0,235	0,264	0,262
1:59:12	50,0	0,110	0,162	1,838	1,905	0,236	0,265	0,261
2:00:12	50,0	0,110	0,162	1,852	1,916	0,236	0,263	0,263
2:01:12	49,9	0,109	0,162	1,866	1,931	0,236	0,263	0,261
2:02:12	49,9	0,109	0,162	1,879	1,943	0,236	0,264	0,262
2:03:12	49,9	0,109	0,162	1,892	1,958	0,236	0,264	0,262
2:04:12	49,9	0,109	0,162	1,906	1,971	0,236	0,264	0,261
2:05:12	50,0	0,109	0,162	1,919	1,984	0,236	0,264	0,262
2:06:12	50,0	0,109	0,162	1,932	1,997	0,236	0,264	0,262
2:07:12	50,0	0,110	0,162	1,946	2,010	0,236	0,264	0,262
2:08:12	50,0	0,113	0,162	1,958	2,023	0,236	0,264	0,262
2:09:12	50,0	0,109	0,162	1,972	2,037	0,236	0,264	0,262
2:10:12	49,9	0,109	0,162	1,985	2,050	0,236	0,264	0,262
2:11:12	49,9	0,108	0,162	1,998	2,063	0,236	0,264	0,262
2:12:12	49,9	0,111	0,162	2,012	2,076	0,236	0,265	0,262
2:13:12	50,0	0,112	0,162	2,024	2,088	0,236	0,264	0,262
2:14:12	50,0	0,108	0,162	2,036	2,102	0,236	0,264	0,262
2:15:12	49,9	0,113	0,162	2,050	2,116	0,237	0,264	0,262
2:16:12	49,9	0,110	0,162	2,063	2,129	0,237	0,264	0,262
2:17:12	49,9	0,107	0,162	2,076	2,141	0,236	0,265	0,263
2:18:12	49,9	0,107	0,162	2,088	2,154	0,236	0,265	0,262
2:19:12	50,0	0,107	0,162	2,102	2,167	0,237	0,264	0,262
2:20:12	50,0	0,107	0,163	2,115	2,180	0,237	0,264	0,262
2:21:12	50,0	0,107	0,163	2,128	2,193	0,237	0,264	0,262
2:22:12	49,9	0,107	0,163	2,139	2,207	0,237	0,264	0,262
2:23:12	49,9	0,107	0,162	2,154	2,217	0,237	0,264	0,262
2:24:12	49,9	0,107	0,163	2,165	2,229	0,237	0,265	0,262
2:25:12	49,9	0,107	0,163	2,177	2,244	0,237	0,264	0,262
2:26:12	49,9	0,107	0,163	2,191	2,256	0,237	0,264	0,262
2:27:12	49,9	0,107	0,163	2,204	2,269	0,237	0,265	0,262

Time	T° 410	A1	A2	B2	C2	D2	E2	F2
2:28:12	49,9	0,107	0,163	2,217	2,280	0,237	0,264	0,262
2:29:12	49,9	0,107	0,163	2,230	2,296	0,237	0,264	0,262
2:30:12	50,0	0,107	0,163	2,242	2,307	0,237	0,265	0,262
2:31:12	50,0	0,107	0,163	2,253	2,320	0,237	0,264	0,262
2:32:12	50,0	0,107	0,163	2,264	2,330	0,237	0,265	0,262
2:33:12	50,0	0,107	0,163	2,279	2,345	0,237	0,264	0,263
2:34:12	50,0	0,107	0,163	2,290	2,357	0,237	0,264	0,262
2:35:12	50,0	0,107	0,163	2,306	2,369	0,237	0,264	0,262
2:36:12	50,0	0,107	0,163	2,317	2,382	0,237	0,264	0,262
2:37:12	49,9	0,107	0,163	2,330	2,394	0,237	0,264	0,262
2:38:12	49,9	0,107	0,163	2,340	2,409	0,237	0,265	0,262
2:39:12	49,9	0,107	0,163	2,353	2,420	0,237	0,264	0,262
2:40:12	49,9	0,107	0,163	2,364	2,430	0,237	0,264	0,262
2:41:12	50,0	0,107	0,163	2,380	2,442	0,237	0,264	0,262
2:42:12	50,0	0,107	0,163	2,388	2,456	0,237	0,264	0,262
2:43:12	49,9	0,107	0,163	2,402	2,469	0,237	0,264	0,262
2:44:12	50,0	0,107	0,163	2,414	2,479	0,237	0,264	0,262
2:45:12	49,9	0,107	0,163	2,427	2,492	0,237	0,264	0,262
2:46:12	49,9	0,107	0,163	2,437	2,503	0,237	0,264	0,262
2:47:12	49,9	0,107	0,163	2,452	2,513	0,237	0,264	0,262
2:48:12	49,9	0,107	0,163	2,463	2,527	0,237	0,264	0,262
2:49:12	49,9	0,107	0,163	2,478	2,540	0,237	0,264	0,262
2:50:12	50,0	0,107	0,163	2,489	2,556	0,238	0,264	0,262
2:51:12	50,0	0,107	0,163	2,499	2,566	0,237	0,264	0,262
2:52:12	50,0	0,107	0,163	2,510	2,579	0,237	0,264	0,262
2:53:12	50,0	0,107	0,163	2,521	2,589	0,238	0,264	0,262
2:54:12	50,0	0,107	0,163	2,535	2,600	0,237	0,264	0,262
2:55:12	49,9	0,112	0,163	2,547	2,619	0,238	0,264	0,262
2:56:12	49,9	0,107	0,163	2,558	2,621	0,238	0,268	0,262
2:57:12	50,0	0,107	0,163	2,572	2,639	0,237	0,264	0,262
2:58:12	49,9	0,107	0,163	2,585	2,647	0,237	0,265	0,262
2:59:12	49,9	0,107	0,163	2,596	2,661	0,238	0,264	0,262
3:00:12	50,0	0,107	0,163	2,608	2,672	0,238	0,263	0,261
3:01:12	49,9	0,107	0,163	2,619	2,684	0,237	0,263	0,261
3:02:12	49,9	0,107	0,163	2,632	2,697	0,238	0,263	0,262
3:03:12	49,9	0,107	0,163	2,643	2,708	0,237	0,263	0,261
3:04:12	49,9	0,107	0,164	2,658	2,720	0,238	0,263	0,262
3:05:12	49,9	0,107	0,163	2,663	2,734	0,238	0,264	0,261
3:06:12	49,9	0,107	0,163	2,678	2,748	0,237	0,263	0,261
3:07:12	50,0	0,107	0,163	2,688	2,749	0,238	0,263	0,261
3:08:12	50,0	0,107	0,163	2,704	2,769	0,238	0,263	0,261
3:09:12	50,0	0,107	0,164	2,713	2,771	0,238	0,263	0,261
3:10:12	50,0	0,107	0,164	2,719	2,787	0,238	0,263	0,261
3:11:12	50,0	0,107	0,164	2,741	2,803	0,238	0,263	0,261
3:12:12	50,0	0,107	0,164	2,753	2,807	0,238	0,264	0,261
3:13:12	50,0	0,107	0,164	2,761	2,825	0,238	0,263	0,261
3:14:12	50,0	0,107	0,164	2,770	2,835	0,238	0,264	0,261
3:15:12	50,0	0,107	0,164	2,780	2,849	0,238	0,263	0,261

Time	T° 410	A1	A2	B2	C2	D2	E2	F2
3:16:12	50,0	0,107	0,164	2,792	2,856	0,238	0,266	0,260
3:17:12	50,0	0,107	0,164	2,804	2,870	0,238	0,264	0,261
3:18:12	50,0	0,107	0,164	2,812	2,880	0,238	0,264	0,261
3:19:12	49,9	0,107	0,163	2,823	2,901	0,237	0,263	0,261
3:20:12	50,0	0,107	0,164	2,848	2,910	0,238	0,263	0,261
3:21:12	50,0	0,107	0,164	2,848	2,911	0,238	0,266	0,261
3:22:12	50,0	0,107	0,164	2,853	2,923	0,238	0,263	0,261
3:23:12	49,9	0,107	0,164	2,876	2,943	0,238	0,263	0,260
3:24:12	49,9	0,107	0,164	2,880	2,946	0,238	0,263	0,260
3:25:12	50,0	0,107	0,164	2,897	2,961	0,237	0,264	0,260
3:26:12	50,0	0,107	0,164	2,908	2,979	0,238	0,263	0,260
3:27:12	50,0	0,107	0,164	2,913	2,980	0,238	0,263	0,260
3:28:12	50,0	0,107	0,164	2,927	2,997	0,238	0,263	0,260
3:29:12	50,0	0,107	0,164	2,938	3,007	0,238	0,263	0,260
3:30:12	50,0	0,107	0,164	2,945	3,000	0,237	0,263	0,260
3:31:12	50,0	0,107	0,164	2,965	3,025	0,238	0,264	0,260
3:32:12	50,0	0,107	0,164	2,973	3,036	0,238	0,263	0,260
3:33:12	49,9	0,107	0,164	2,979	3,053	0,238	0,263	0,260
3:34:12	49,9	0,107	0,164	2,994	3,062	0,238	0,263	0,260
3:35:12	49,9	0,107	0,164	3,002	3,065	0,238	0,263	0,260
3:36:12	49,9	0,107	0,164	3,010	3,079	0,238	0,263	0,260
3:37:12	50,0	0,107	0,164	3,022	3,098	0,238	0,268	0,260
3:38:12	50,0	0,107	0,164	3,029	3,113	0,238	0,267	0,260
3:39:12	50,0	0,107	0,164	3,044	3,112	0,238	0,268	0,260
3:40:12	50,0	0,107	0,164	3,052	3,123	0,238	0,267	0,259
3:41:12	49,9	0,107	0,164	3,058	3,120	0,238	0,267	0,259
3:42:12	50,0	0,107	0,164	3,081	3,150	0,238	0,263	0,259
3:43:12	50,0	0,107	0,164	3,088	3,146	0,238	0,263	0,259
3:44:12	50,0	0,107	0,164	3,106	3,163	0,238	0,263	0,259
3:45:12	50,0	0,107	0,164	3,108	3,186	0,238	0,263	0,260
3:46:12	50,0	0,108	0,164	3,130	3,179	0,238	0,263	0,259
3:47:12	49,9	0,107	0,164	3,142	3,199	0,238	0,263	0,259
3:48:12	49,9	0,107	0,164	3,138	3,203	0,238	0,263	0,259
3:49:12	49,9	0,108	0,164	3,154	3,213	0,238	0,263	0,259
3:50:12	49,9	0,107	0,164	3,173	3,232	0,238	0,263	0,259
3:51:12	49,9	0,109	0,164	3,162	3,244	0,238	0,263	0,258
3:52:12	49,9	0,107	0,164	3,190	3,246	0,238	0,263	0,259
3:53:12	50,0	0,112	0,164	3,186	3,245	0,237	0,263	0,258
3:54:12	49,9	0,107	0,164	3,194	3,256	0,237	0,263	0,258
3:55:12	50,0	0,107	0,164	3,222	3,282	0,238	0,263	0,258
3:56:12	50,0	0,107	0,164	3,216	3,286	0,238	0,263	0,258
3:57:12	50,0	0,107	0,164	3,223	3,314	0,237	0,263	0,258
3:58:12	50,0	0,107	0,164	3,243	3,335	0,237	0,263	0,258
3:59:12	49,9	0,108	0,164	3,228	3,298	0,238	0,263	0,258
4:00:12	49,9	0,107	0,164	3,262	3,329	0,237	0,262	0,258

Time	A1	A2	B2	C2	D2	E2	F2
0:00:12	0,000	0,024	0,028	0,036	0,032	0,038	0,039
0:01:12	0,000	0,051	0,050	0,070	0,065	0,074	0,074
0:02:12	0,000	0,051	0,050	0,081	0,071	0,079	0,081
0:03:12	0,000	0,051	0,056	0,092	0,075	0,084	0,086
0:04:12	0,000	0,051	0,060	0,105	0,079	0,090	0,092
0:05:12	0,000	0,051	0,069	0,120	0,082	0,094	0,096
0:06:12	0,000	0,051	0,079	0,137	0,085	0,098	0,100
0:07:12	0,000	0,052	0,091	0,153	0,088	0,101	0,103
0:08:12	0,000	0,052	0,105	0,168	0,090	0,104	0,106
0:09:12	0,000	0,052	0,122	0,183	0,092	0,107	0,108
0:10:12	0,000	0,052	0,140	0,199	0,094	0,109	0,111
0:11:12	0,000	0,052	0,160	0,213	0,095	0,112	0,113
0:12:12	0,000	0,052	0,178	0,228	0,097	0,113	0,115
0:13:12	0,000	0,049	0,189	0,240	0,095	0,112	0,113
0:14:12	0,000	0,049	0,204	0,255	0,097	0,113	0,115
0:15:12	0,000	0,046	0,216	0,268	0,095	0,112	0,114
0:16:12	0,000	0,047	0,232	0,284	0,096	0,114	0,117
0:17:12	0,000	0,048	0,248	0,301	0,099	0,117	0,119
0:18:12	0,000	0,046	0,261	0,313	0,097	0,116	0,117
0:19:12	0,000	0,046	0,276	0,329	0,098	0,117	0,119
0:20:12	0,000	0,048	0,293	0,346	0,101	0,120	0,121
0:21:12	0,000	0,046	0,306	0,359	0,100	0,119	0,120
0:22:12	0,000	0,047	0,322	0,376	0,101	0,121	0,122
0:23:12	0,000	0,049	0,339	0,393	0,104	0,124	0,125
0:24:12	0,000	0,046	0,352	0,406	0,102	0,122	0,123
0:25:12	0,000	0,046	0,367	0,421	0,102	0,123	0,124
0:26:12	0,000	0,048	0,384	0,439	0,104	0,125	0,126
0:27:12	0,000	0,049	0,401	0,456	0,107	0,128	0,129
0:28:12	0,000	0,050	0,417	0,472	0,108	0,129	0,130
0:29:12	0,000	0,051	0,433	0,488	0,109	0,131	0,132
0:30:12	0,000	0,051	0,448	0,504	0,110	0,131	0,132
0:31:12	0,000	0,051	0,463	0,519	0,111	0,132	0,133
0:32:12	0,000	0,051	0,479	0,535	0,111	0,133	0,134
0:33:12	0,000	0,050	0,492	0,548	0,110	0,132	0,133
0:34:12	0,000	0,048	0,506	0,562	0,109	0,131	0,132
0:35:12	0,000	0,048	0,521	0,577	0,109	0,130	0,132
0:36:12	0,000	0,048	0,535	0,592	0,109	0,131	0,132
0:37:12	0,000	0,048	0,551	0,607	0,109	0,131	0,133
0:38:12	0,000	0,048	0,566	0,623	0,110	0,132	0,133
0:39:12	0,000	0,048	0,581	0,638	0,110	0,132	0,134
0:40:12	0,000	0,047	0,596	0,653	0,110	0,132	0,134
0:41:12	0,000	0,047	0,611	0,668	0,110	0,133	0,134
0:42:12	0,000	0,047	0,626	0,683	0,111	0,133	0,135
0:43:12	0,000	0,047	0,641	0,698	0,111	0,133	0,135
0:44:12	0,000	0,047	0,656	0,713	0,111	0,134	0,136
0:45:12	0,000	0,047	0,671	0,728	0,112	0,134	0,136
0:46:12	0,000	0,047	0,686	0,744	0,112	0,135	0,137
0:47:12	0,000	0,047	0,701	0,758	0,112	0,135	0,137

Time	A1	A2	B2	C2	D2	E2	F2
0:48:12	0,000	0,047	0,716	0,774	0,113	0,136	0,137
0:49:12	0,000	0,047	0,732	0,789	0,113	0,136	0,138
0:50:12	0,000	0,048	0,747	0,804	0,113	0,137	0,138
0:51:12	0,000	0,048	0,762	0,820	0,114	0,138	0,139
0:52:12	0,000	0,048	0,777	0,835	0,114	0,138	0,139
0:53:12	0,000	0,048	0,792	0,850	0,115	0,138	0,140
0:54:12	0,000	0,049	0,808	0,865	0,115	0,139	0,140
0:55:12	0,000	0,049	0,823	0,881	0,116	0,140	0,141
0:56:12	0,000	0,049	0,838	0,896	0,116	0,140	0,141
0:57:12	0,000	0,049	0,853	0,911	0,117	0,141	0,142
0:58:12	0,000	0,049	0,867	0,926	0,117	0,141	0,142
0:59:12	0,000	0,049	0,882	0,941	0,117	0,141	0,142
1:00:12	0,000	0,049	0,897	0,956	0,117	0,142	0,143
1:01:12	0,000	0,049	0,912	0,971	0,118	0,142	0,144
1:02:12	0,000	0,050	0,927	0,986	0,118	0,142	0,144
1:03:12	0,000	0,049	0,942	1,001	0,118	0,143	0,144
1:04:12	0,000	0,050	0,956	1,016	0,118	0,143	0,145
1:05:12	0,000	0,050	0,971	1,031	0,119	0,147	0,145
1:06:12	0,000	0,050	0,986	1,045	0,119	0,149	0,145
1:07:12	0,000	0,050	1,000	1,060	0,119	0,148	0,145
1:08:12	0,000	0,050	1,015	1,075	0,120	0,149	0,146
1:09:12	0,000	0,050	1,030	1,090	0,120	0,149	0,146
1:10:12	0,000	0,050	1,044	1,104	0,120	0,148	0,146
1:11:12	0,000	0,050	1,058	1,119	0,120	0,150	0,146
1:12:12	0,000	0,050	1,073	1,134	0,120	0,149	0,146
1:13:12	0,000	0,050	1,088	1,148	0,120	0,149	0,147
1:14:12	0,000	0,050	1,103	1,163	0,120	0,149	0,147
1:15:12	0,000	0,050	1,117	1,178	0,121	0,150	0,147
1:16:12	0,000	0,050	1,132	1,193	0,121	0,151	0,147
1:17:12	0,000	0,051	1,146	1,207	0,121	0,150	0,147
1:18:12	0,000	0,051	1,161	1,222	0,122	0,151	0,148
1:19:12	0,000	0,051	1,175	1,236	0,122	0,151	0,148
1:20:12	0,000	0,051	1,190	1,251	0,122	0,151	0,149
1:21:12	0,000	0,052	1,204	1,266	0,123	0,152	0,149
1:22:12	0,000	0,051	1,219	1,280	0,123	0,152	0,149
1:23:12	0,000	0,052	1,234	1,295	0,123	0,152	0,150
1:24:12	0,000	0,052	1,248	1,309	0,123	0,152	0,150
1:25:12	0,000	0,052	1,261	1,323	0,124	0,153	0,150
1:26:12	0,000	0,052	1,276	1,338	0,124	0,153	0,150
1:27:12	0,000	0,053	1,290	1,352	0,124	0,154	0,151
1:28:12	0,000	0,053	1,304	1,367	0,124	0,154	0,151
1:29:12	0,000	0,053	1,319	1,381	0,124	0,155	0,150
1:30:12	0,000	0,053	1,333	1,395	0,125	0,154	0,152
1:31:12	0,000	0,053	1,347	1,410	0,125	0,154	0,151
1:32:12	0,000	0,053	1,361	1,424	0,125	0,156	0,152
1:33:12	0,000	0,053	1,376	1,438	0,126	0,156	0,152
1:34:12	0,000	0,054	1,390	1,453	0,126	0,156	0,152
1:35:12	0,000	0,054	1,404	1,467	0,126	0,156	0,152

Time	A1	A2	B2	C2	D2	E2	F2
1:36:12	0,000	0,054	1,418	1,481	0,126	0,156	0,152
1:37:12	0,000	0,054	1,432	1,495	0,126	0,156	0,152
1:38:12	0,000	0,054	1,446	1,509	0,127	0,156	0,153
1:39:12	0,000	0,054	1,459	1,523	0,127	0,156	0,153
1:40:12	0,000	0,054	1,474	1,537	0,127	0,157	0,153
1:41:12	0,000	0,054	1,488	1,551	0,127	0,156	0,154
1:42:12	0,000	0,055	1,501	1,565	0,127	0,157	0,153
1:43:12	0,000	0,055	1,515	1,579	0,128	0,157	0,153
1:44:12	0,000	0,055	1,529	1,593	0,128	0,157	0,153
1:45:12	0,000	0,054	1,542	1,606	0,127	0,157	0,154
1:46:12	0,000	0,053	1,555	1,619	0,126	0,156	0,152
1:47:12	0,000	0,049	1,564	1,628	0,122	0,151	0,148
1:48:12	0,000	0,049	1,577	1,642	0,122	0,151	0,147
1:49:12	0,000	0,049	1,591	1,655	0,122	0,151	0,148
1:50:12	0,000	0,049	1,605	1,669	0,122	0,151	0,148
1:51:12	0,000	0,049	1,618	1,683	0,122	0,151	0,148
1:52:12	0,000	0,051	1,634	1,698	0,124	0,153	0,150
1:53:12	0,000	0,049	1,646	1,710	0,122	0,151	0,148
1:54:12	0,000	0,052	1,663	1,727	0,126	0,155	0,152
1:55:12	0,000	0,052	1,676	1,741	0,126	0,155	0,151
1:56:12	0,000	0,052	1,689	1,754	0,126	0,154	0,151
1:57:12	0,000	0,051	1,702	1,766	0,125	0,154	0,150
1:58:12	0,000	0,049	1,713	1,778	0,123	0,152	0,150
1:59:12	0,000	0,052	1,728	1,794	0,125	0,154	0,151
2:00:12	0,000	0,052	1,742	1,806	0,126	0,153	0,152
2:01:12	0,000	0,053	1,757	1,822	0,127	0,154	0,152
2:02:12	0,000	0,053	1,770	1,834	0,126	0,154	0,153
2:03:12	0,000	0,053	1,783	1,848	0,127	0,154	0,152
2:04:12	0,000	0,053	1,797	1,862	0,127	0,155	0,152
2:05:12	0,000	0,053	1,810	1,875	0,127	0,155	0,152
2:06:12	0,000	0,053	1,823	1,888	0,127	0,154	0,152
2:07:12	0,000	0,052	1,835	1,899	0,126	0,154	0,152
2:08:12	0,000	0,050	1,845	1,910	0,123	0,151	0,149
2:09:12	0,000	0,053	1,863	1,928	0,128	0,155	0,153
2:10:12	0,000	0,053	1,876	1,941	0,127	0,155	0,153
2:11:12	0,000	0,054	1,890	1,955	0,128	0,156	0,154
2:12:12	0,000	0,051	1,901	1,964	0,125	0,153	0,150
2:13:12	0,000	0,051	1,912	1,977	0,125	0,152	0,150
2:14:12	0,000	0,054	1,928	1,993	0,128	0,156	0,154
2:15:12	0,000	0,050	1,938	2,003	0,124	0,152	0,149
2:16:12	0,000	0,052	1,953	2,019	0,127	0,154	0,152
2:17:12	0,000	0,055	1,969	2,034	0,130	0,158	0,156
2:18:12	0,000	0,055	1,981	2,047	0,130	0,158	0,155
2:19:12	0,000	0,055	1,994	2,060	0,130	0,157	0,155
2:20:12	0,000	0,056	2,008	2,073	0,130	0,157	0,155
2:21:12	0,000	0,056	2,021	2,086	0,130	0,157	0,155
2:22:12	0,000	0,056	2,032	2,100	0,130	0,157	0,155
2:23:12	0,000	0,055	2,047	2,111	0,130	0,157	0,155

Time	A1	A2	B2	C2	D2	E2	F2
2:24:12	0,000	0,056	2,058	2,123	0,130	0,158	0,155
2:25:12	0,000	0,056	2,070	2,137	0,130	0,157	0,155
2:26:12	0,000	0,056	2,084	2,149	0,130	0,157	0,155
2:27:12	0,000	0,056	2,096	2,162	0,130	0,158	0,155
2:28:12	0,000	0,056	2,110	2,173	0,130	0,157	0,155
2:29:12	0,000	0,056	2,123	2,189	0,130	0,157	0,155
2:30:12	0,000	0,056	2,135	2,200	0,130	0,158	0,155
2:31:12	0,000	0,056	2,146	2,213	0,130	0,157	0,155
2:32:12	0,000	0,056	2,157	2,223	0,130	0,158	0,155
2:33:12	0,000	0,056	2,172	2,238	0,130	0,157	0,156
2:34:12	0,000	0,056	2,183	2,250	0,130	0,157	0,156
2:35:12	0,000	0,056	2,199	2,262	0,130	0,157	0,155
2:36:12	0,000	0,056	2,210	2,275	0,130	0,157	0,155
2:37:12	0,000	0,056	2,223	2,287	0,130	0,157	0,155
2:38:12	0,000	0,056	2,233	2,302	0,130	0,158	0,155
2:39:12	0,000	0,056	2,246	2,313	0,131	0,157	0,155
2:40:12	0,000	0,056	2,257	2,324	0,130	0,157	0,155
2:41:12	0,000	0,056	2,273	2,336	0,130	0,157	0,155
2:42:12	0,000	0,056	2,281	2,349	0,130	0,157	0,155
2:43:12	0,000	0,056	2,295	2,362	0,131	0,157	0,155
2:44:12	0,000	0,056	2,308	2,372	0,131	0,157	0,155
2:45:12	0,000	0,056	2,320	2,385	0,131	0,157	0,155
2:46:12	0,000	0,056	2,330	2,397	0,131	0,157	0,155
2:47:12	0,000	0,056	2,345	2,407	0,131	0,158	0,155
2:48:12	0,000	0,056	2,356	2,420	0,130	0,157	0,155
2:49:12	0,000	0,056	2,371	2,433	0,131	0,157	0,155
2:50:12	0,000	0,056	2,382	2,450	0,131	0,157	0,155
2:51:12	0,000	0,056	2,392	2,459	0,131	0,157	0,155
2:52:12	0,000	0,056	2,403	2,472	0,130	0,157	0,155
2:53:12	0,000	0,056	2,414	2,482	0,131	0,157	0,155
2:54:12	0,000	0,056	2,428	2,493	0,131	0,157	0,155
2:55:12	0,000	0,051	2,435	2,507	0,125	0,152	0,149
2:56:12	0,000	0,056	2,451	2,514	0,131	0,161	0,155
2:57:12	0,000	0,056	2,465	2,533	0,131	0,157	0,155
2:58:12	0,000	0,056	2,478	2,541	0,131	0,158	0,155
2:59:12	0,000	0,056	2,489	2,554	0,131	0,157	0,155
3:00:12	0,000	0,057	2,501	2,566	0,131	0,156	0,155
3:01:12	0,000	0,056	2,512	2,577	0,131	0,157	0,155
3:02:12	0,000	0,056	2,526	2,590	0,131	0,156	0,155
3:03:12	0,000	0,056	2,536	2,601	0,131	0,157	0,154
3:04:12	0,000	0,057	2,551	2,613	0,131	0,156	0,155
3:05:12	0,000	0,056	2,556	2,627	0,131	0,157	0,154
3:06:12	0,000	0,056	2,571	2,642	0,131	0,156	0,154
3:07:12	0,000	0,056	2,581	2,642	0,131	0,156	0,154
3:08:12	0,000	0,056	2,597	2,662	0,131	0,156	0,154
3:09:12	0,000	0,057	2,606	2,664	0,131	0,156	0,154
3:10:12	0,000	0,057	2,612	2,680	0,131	0,156	0,154
3:11:12	0,000	0,057	2,634	2,696	0,131	0,156	0,154

Time	A1	A2	B2	C2	D2	E2	F2
3:12:12	0,000	0,057	2,646	2,700	0,131	0,157	0,154
3:13:12	0,000	0,057	2,654	2,717	0,131	0,156	0,154
3:14:12	0,000	0,057	2,663	2,728	0,131	0,157	0,154
3:15:12	0,000	0,057	2,673	2,742	0,131	0,156	0,154
3:16:12	0,000	0,057	2,685	2,749	0,131	0,159	0,154
3:17:12	0,000	0,057	2,697	2,763	0,131	0,157	0,154
3:18:12	0,000	0,057	2,705	2,773	0,131	0,157	0,154
3:19:12	0,000	0,057	2,717	2,794	0,131	0,156	0,154
3:20:12	0,000	0,057	2,741	2,803	0,131	0,156	0,154
3:21:12	0,000	0,057	2,741	2,804	0,131	0,159	0,154
3:22:12	0,000	0,057	2,747	2,816	0,131	0,157	0,154
3:23:12	0,000	0,057	2,769	2,837	0,131	0,157	0,154
3:24:12	0,000	0,057	2,773	2,839	0,131	0,156	0,153
3:25:12	0,000	0,057	2,790	2,854	0,131	0,157	0,153
3:26:12	0,000	0,057	2,801	2,872	0,131	0,156	0,153
3:27:12	0,000	0,057	2,806	2,874	0,131	0,156	0,153
3:28:12	0,000	0,057	2,820	2,890	0,131	0,157	0,153
3:29:12	0,000	0,057	2,831	2,900	0,131	0,156	0,153
3:30:12	0,000	0,057	2,838	2,893	0,131	0,156	0,153
3:31:12	0,000	0,057	2,858	2,918	0,131	0,157	0,153
3:32:12	0,000	0,057	2,866	2,929	0,131	0,156	0,153
3:33:12	0,000	0,057	2,872	2,946	0,131	0,156	0,153
3:34:12	0,000	0,057	2,887	2,955	0,131	0,156	0,153
3:35:12	0,000	0,057	2,895	2,958	0,131	0,156	0,153
3:36:12	0,000	0,057	2,903	2,971	0,131	0,156	0,153
3:37:12	0,000	0,057	2,915	2,991	0,131	0,161	0,153
3:38:12	0,000	0,057	2,921	3,006	0,131	0,160	0,153
3:39:12	0,000	0,057	2,937	3,005	0,131	0,161	0,153
3:40:12	0,000	0,057	2,945	3,016	0,131	0,160	0,152
3:41:12	0,000	0,057	2,951	3,013	0,131	0,160	0,152
3:42:12	0,000	0,057	2,974	3,043	0,131	0,156	0,152
3:43:12	0,000	0,057	2,981	3,039	0,131	0,156	0,152
3:44:12	0,000	0,057	2,999	3,057	0,131	0,156	0,152
3:45:12	0,000	0,057	3,001	3,078	0,131	0,156	0,153
3:46:12	0,000	0,056	3,023	3,072	0,130	0,155	0,151
3:47:12	0,000	0,057	3,035	3,092	0,131	0,156	0,152
3:48:12	0,000	0,057	3,031	3,096	0,131	0,156	0,152
3:49:12	0,000	0,056	3,047	3,106	0,130	0,155	0,151
3:50:12	0,000	0,057	3,066	3,125	0,131	0,156	0,151
3:51:12	0,000	0,056	3,053	3,135	0,129	0,154	0,150
3:52:12	0,000	0,057	3,083	3,139	0,131	0,156	0,152
3:53:12	0,000	0,052	3,074	3,133	0,125	0,151	0,146
3:54:12	0,000	0,057	3,087	3,149	0,130	0,156	0,151
3:55:12	0,000	0,057	3,115	3,175	0,130	0,156	0,151
3:56:12	0,000	0,057	3,109	3,179	0,131	0,156	0,151
3:57:12	0,000	0,057	3,116	3,207	0,131	0,156	0,151
3:58:12	0,000	0,057	3,136	3,227	0,130	0,156	0,151
3:59:12	0,000	0,057	3,120	3,191	0,130	0,155	0,150

Blank 410 (continued)

Time	A1	A2	B2	C2	D2	E2	F2
4:00:12	0,000	0,057	3,155	3,222	0,130	0,155	0,151

Results

	1	2	3	4	5	6	
A	0,000<br>1,000<br>0:02:12<br>?????	5,450<br>0,511<br>0:02:12<br>?????					Max V [Blank 410]<br>R-Squared [Blank 410]<br>t at Max V [Blank 410]<br>Lagtime [Blank 410]
B		18,350<br>0,999<br>0:10:12<br>0:04:04					Max V [Blank 410]<br>R-Squared [Blank 410]<br>t at Max V [Blank 410]<br>Lagtime [Blank 410]
C		19,000<br>0,970<br>3:56:12<br>1:10:18					Max V [Blank 410]<br>R-Squared [Blank 410]<br>t at Max V [Blank 410]<br>Lagtime [Blank 410]
D		10,380<br>0,760<br>0:02:12<br>?????					Max V [Blank 410]<br>R-Squared [Blank 410]<br>t at Max V [Blank 410]<br>Lagtime [Blank 410]
E		11,340<br>0,769<br>0:02:12<br>?????					Max V [Blank 410]<br>R-Squared [Blank 410]<br>t at Max V [Blank 410]<br>Lagtime [Blank 410]
F		11,950<br>0,802<br>0:02:12<br>?????					Max V [Blank 410]<br>R-Squared [Blank 410]<br>t at Max V [Blank 410]<br>Lagtime [Blank 410]
G							Max V [Blank 410]<br>R-Squared [Blank 410]<br>t at Max V [Blank 410]<br>Lagtime [Blank 410]
H							Max V [Blank 410]<br>R-Squared [Blank 410]<br>t at Max V [Blank 410]<br>Lagtime [Blank 410]

Results (continued)

	7	8	9	10	11	12	
A							Max V [Blank 410]<br>R-Squared [Blank 410]<br>t at Max V [Blank 410]<br>Lagtime [Blank 410]
B							Max V [Blank 410]<br>R-Squared [Blank 410]<br>t at Max V [Blank 410]<br>Lagtime [Blank 410]
C							Max V [Blank 410]<br>R-Squared [Blank 410]<br>t at Max V [Blank 410]<br>Lagtime [Blank 410]
D							Max V [Blank 410]<br>R-Squared [Blank 410]<br>t at Max V [Blank 410]<br>Lagtime [Blank 410]
E							Max V [Blank 410]<br>R-Squared [Blank 410]<br>t at Max V [Blank 410]<br>Lagtime [Blank 410]
F							Max V [Blank 410]<br>R-Squared [Blank 410]<br>t at Max V [Blank 410]<br>Lagtime [Blank 410]
G							Max V [Blank 410]<br>R-Squared [Blank 410]<br>t at Max V [Blank 410]<br>Lagtime [Blank 410]
H							Max V [Blank 410]<br>R-Squared [Blank 410]<br>t at Max V [Blank 410]<br>Lagtime [Blank 410]
