# Supplementary material for: Functional control of a 0.5 MDa TET aminopeptidase by a flexible loop revealed by MAS NMR
Source: Nat Commun. 2022 Apr 8;13:1927. doi: 10.1038/s41467-022-29423-0 (PMC8993905; doi:10.1038/s41467-022-29423-0)
Supplement: Supplementary file 4 — Source data [file 41467_2022_29423_MOESM4_ESM.gz › source_data_2022/figures_SI17_SI18/S17-18/activity_freeHis_Iva.zip!/activity_freeHis_Iva/iva2309/20210923Gen5 - Protocol1 Detail.pdf]

Protocol Definition Report: Protocol1

Procedure

Plate Type	96 WELL PLATE (Use plate lid)
Eject plate on completion	
Set Temperature	Setpoint 50°C
	Preheat before moving to next step
Delay	0:20:00 (HH:MM:SS)
Plate Out/In	add substrate
Shake	Medium, 0:10 (MM:SS)
Start Kinetic	Runtime 4:00:00 (HH:MM:SS), Interval 0:01:00, 241 Reads
Read	Absorbance Endpoint
	Full Plate
	Wavelengths: 410
	Read Speed: Normal, Delay: 100 msec, Measurements/Data Point: 8
End Kinetic	

Plate Layout

	1	2	3	4	5	6	7	8	9	10	11	12	
A	BLK	SPL1											Well ID
B		SPL1											Well ID
C		SPL1											Well ID
D		SPL2											Well ID
E		SPL2											Well ID
F		SPL2											Well ID
G													Well ID
H													Well ID

Data Reduction

Blank Transformation	Data In: 410
	Well ID: BLK
	Data Set Name: Blank 410
	Formula: X-BLK
Kinetic Analysis	Data In: Blank 410
	Max V
